# Integrated effects of pre-harvest high blue light and postharvest sodium nitroprusside on volatile oil composition and quality of cold-stored holy basil

**DOI:** 10.1016/j.fochx.2026.103493

**Published:** 2026-01-05

**Authors:** Thanaboon Plakunmonthon, Panita Chutimanukul, Kenji Matsui, Kanogwan Seraypheap

**Affiliations:** a Biological Sciences Program, Faculty of Science, Chulalongkorn University, Bangkok, Thailand; b Center of Excellence in Environment and Plant Physiology, Department of Botany, Faculty of Science, Chulalongkorn University, Bangkok, Thailand; c National Center for Genetic Engineering and Biotechnology (BIOTEC), National Science and Technology Development Agency, Klong Nueng, Klong Luang, Thailand; d Graduate School of Sciences and Technology for Innovation, Yamaguchi University, Yamaguchi 753-8515, Japan

**Keywords:** LED light, Nitric oxide, Antioxidant, Β-Caryophyllene, Humulene

## Abstract

Holy basil (*Ocimum tenuiflorum* L.) is highly susceptible to chilling injury (CI) during postharvest storage, which limiting marketability and storage life. This study examined whether white or high-intensity blue light during cultivation, combined with postharvest sodium nitroprusside (SNP) treatment, alleviates CI and modulates volatile oil composition in green holy basil under cold storage. Plants were grown under white or high-intensity blue light and subsequently treated with 0, 100, 200, or 300 μM SNP before storage at 10 °C. Basil grown under high blue light and treated with 200 μM SNP showed the lowest electrolyte leakage and malondialdehyde levels, indicating reduced membrane damage and oxidative stress. High blue light also maintained higher antioxidant capacity and preserved key volatile compounds, including β-caryophyllene and humulene. Integrating high blue light with optimal SNP application effectively mitigates chilling-induced oxidative damage and improves postharvest quality, offering a practical strategy to extend shelf life in holy basil.

## Highlights

• High blue light during cultivation enhanced chilling tolerance in holy basil.
• White and high blue light with 200 μM SNP showed the lowest malondialdehyde levels.
• 300 μM SNP increased antioxidant capacity under high blue light conditions.
• β-caryophyllene and humulene were highest at 200 μM SNP treatment.

## 1 Introduction

Holy basil (*Ocimum tenuiflorum* L.) is an increasingly recognized herbal plant as a valuable crop with diverse industrial potential, driven by growing awareness of its health benefits. Its cultivation and trade offer opportunities to stimulate both local and global economies. Holy basil leaves are rich in three primary chemical classes: monoterpenes, sesquiterpenes, and phenylpropanoids. These compounds exhibit a range of bioactive properties. Specifically, linalool demonstrates potent antibacterial and antifungal activities (Herman et al., 2016). The sesquiterpene β-caryophyllene provides anti-inflammatory and antioxidant benefits (Yang et al., 2017). Humulene (α-caryophyllene) offers anti-inflammatory and analgesic effects, and promotes wound healing (Rogerio et al., 2009). Eugenol, a key phenylpropanoid, exhibits antibacterial, anti-inflammatory, and antioxidant activities and has been traditionally employed in the treatment of multiple conditions (Sharma et al., 2022). The accumulation of these bioactive chemicals is significantly influenced by several pre-harvest conditions, including temperature, light intensity, water availability, and nutrition (Chutimanukul, Wanichananan, et al., 2022; Sale et al., 2023).

In herbal plant cultivation, supplemental lighting not only increases secondary metabolite production in plants but also extends the storage life of vegetables (Loi et al., 2020). Due to their low power consumption and environmentally benign operation, artificial light sources known as light-emitting diodes (LEDs) are highly energy-efficient and produce low heat emissions. Red light, with an emission peak around 660 nm, affects stem elongation, leaf growth, chlorophyll content, and the development of the photosynthetic apparatus (Rabara et al., 2017). It also promotes significant increases in plant height of sweet basil (Sale et al., 2023). Blue light, within the approximate wavelength range of 400 to 500 nm, contributes significantly to the control of several physiological functions in plants, including stomatal differentiation and opening, leaf expansion and structural development, and the levels of hormonal regulators (Jensen et al., 2018). Sale et al. (2023) found that blue light has been shown to promote basil leaf expansion and increase phenolic content and phenylalanine ammonia-lyase (PAL) activity. Moreover, several studies have indicated that red and blue light quality influences photosynthetic efficiency, antioxidant activity, and vegetable growth (Naznin et al., 2019), while their combined application enhances volatile compound synthesis (Sale et al., 2023). Fresh vegetables are typically stored at low temperatures to slow leaf senescence and prolong their postharvest life. However, many tropical fresh products are susceptible to chilling injury (CI) when kept under cold conditions. In basil, it is well established that storage at temperatures below 12 °C induces CI symptoms such as stem browning, leaf wilting, loss of leaf gloss, and aroma alteration, while storage above 15 °C accelerates senescence. Therefore, maintaining storage around 10–12 °C has been suggested as an optimal compromise to minimize CI while retaining freshness (Cozzolino et al., 2016).

Numerous studies have revealed that nitric oxide (NO) contributes to improved plant tolerance against biotic and abiotic challenges while influencing secondary metabolite production (Bai et al., 2024; Nabi et al., 2019). Sodium nitroprusside (SNP) is now widely recognized for its role in releasing NO, which acts as a signaling molecule in plants and it is considered safe for use in research aimed at prolonging the longevity of harvested horticultural crops. SNP application has been suggested as a potential method to mitigate CI in horticulture products after harvest (Lu et al., 2023). However, its utilization in vegetables has been less common. According to Lu et al. (2023), SNP alleviated CI and limited the increase in membrane permeability, thereby preserving superior cucumber quality. Additionally, applying exogenous SNP can alleviate the yellowing of postharvest broccoli by suppressing chlorophyll degradation. Furthermore, treating broccoli with exogenous SNP slowed the decline of its nutritional quality during storage at 4 °C (Zou et al., 2025). Moreover, SNP application promotes the production of secondary metabolites in marjoram herbs (Farouk & Al-Huqail, 2020). Despite these advances, there has been little effort to integrate pre-harvest environmental modulation (such as LED light spectra) with postharvest SNP application to explore their synergistic effect on alleviating CI in leafy herbs like holy basil. This combined strategy represents a novel approach that bridges production and postharvest handling stages, addressing quality deterioration from farm to market.

To date, no studies have reported the effects of postharvest SNP treatment on holy basil cultivated under different light conditions and stored at low temperatures. While previous studies have examined the effects of light quality or NO treatments independently, their combined influence on postharvest physiology and aroma-related compounds in holy basil has not been documented. This study addresses this knowledge gap by integrating pre-harvest light spectrum modulation with postharvest SNP application to improve chilling tolerance, physiological quality, and volatile oil preservation during storage at 10 °C. Accordingly, the objective of this study was to evaluate the combined effects of pre-harvest light spectrum manipulation and postharvest SNP application on physiological responses and volatile oil composition of holy basil during cold storage.

From a practical perspective, this integrated strategy is particularly relevant given the high susceptibility of holy basil to chilling injury and offers a sustainable, low-cost approach to extending shelf life and maintaining aroma quality during cold-chain distribution. Such an approach has potential to support export-oriented supply chains and enhance the commercial value of basil for both smallholder growers and industrial processors.

## 2 Materials and methods

### 2.1 Plant materials and treatments

Green holy basil seeds acquired from Chia Tai Co. Ltd. (Bangkok, Thailand) were germinated under 150 μmol m^−2^ s^−1^ LED light intensity, a 16-h photoperiod, 25 ± 1.5 °C temperature, and 70 ± 5 % humidity. Fourteen-day-old seedlings were moved to a deep-flow technique (DFT) hydroponic system with a modified Enshi nutrient solution (pH 6.5 ± 0.15, EC 2 ± 0.02 mS), following the methods of Chutimanukul, Wanichananan, et al. (2022). After transplantation, plants were exposed to two light spectrum treatments: white light (λ = 400–780 nm) as the control and high blue light with a red-to-blue (R:B) ratio of 1:3. Blue (λ = 400–500 nm, peak at 450 nm) and red (λ = 600–700 nm, peak at 660 nm) LEDs supplied by AGRI-OPTECH Co., Ltd. (Taiwan) were used as light sources. This light regime was selected based on previous findings by Chutimanukul, Wanichananan, et al. (2022), who reported that an R:B ratio of 1:3 resulted in the highest biomass accumulation in holy basil leaves. The PPFDs of light were measured at the bench surface level and maintained at 200 μmol·m^−2^·s^−1^ for all treatments. A light meter (Sekonic C-7000, Japan) was used to measure the spectral distributions of the light treatments (Supplementary Document S1). All plants were placed in the same room; however, the experimental areas were physically partitioned to prevent light contamination between treatments.

Three-month-old holy basil stem segments with leaves were harvested and trimmed to a length of 20 to 25 cm. Following harvest, each sample was sprayed with a foliar SNP (Sigma-Aldrich, Germany) solution at concentrations of 100, 200, or 300 μM (20 mL per 30 ± 5 g sample), while distilled water served as the control. The selected concentration range was determined based on previous experiments by Gohari et al. (2020) and was further validated through preliminary testing prior to implementation in this study. After spraying, all samples were air-dried at room temperature for 10 min to remove surface moisture and were subsequently packaged in low-density polyethylene (LDPE) bags, with 10 plants allocated per treatment unit. Storage was conducted at 10 °C and 80–85 % RH, with data collected on days 0, 3, 6, and 9, or until the end of postharvest life.

### 2.2 Weight loss

From the beginning of the experiment, weight loss was assessed. First, the samples were weighed (day 0), and then the fresh weight changes (day t) were measured. The following formula was used to get the weight reduction percentage:

Weight loss (%) = [(W0 - Wt) / W0] × 100.

where W0 is the initial weight and Wt is the sample weight at day t.

### 2.3 Senescence symptom score

Chilling injury (CI) and storage life were evaluated using a 1–5 senescence symptom scoring system based on leaf turgidity, browning associated with physiological deterioration, and pathogen-related damage, following Patiño et al. (2018) with slight modifications.

A score of 1 indicated fresh samples of acceptable commercial quality with no visible senescence symptoms. A score of 2 represented very slight senescence, with less than 5 % of leaves affected by injuries smaller than 3 mm in diameter, while maintaining freshness and turgidity. A score of 3 denoted minor quality deterioration, characterized by injuries of 3–10 mm affecting up to 10 % of leaves and mild water loss; samples at this stage remained marketable. A score of 4 reflected substantial quality deterioration, with up to 20 % of leaves showing injuries up to 10 mm in diameter, moderate desiccation, and loss of turgidity, rendering the samples unmarketable. A score of 5 indicated severe senescence, with more than 20 % of leaves exhibiting physiological or pathological damage, leaf abscission, dryness, and poor overall quality. Samples reaching a score of 4 were considered to have reached the end of their storage life.

### 2.4 Electrolyte leakage (EL)

EL was assessed using the procedure of Jiang and Huang (2002). Holy basil leaves were harvested and trimmed into 1 cm discs (3 leaves from each plant). Following three cleanings, the samples were left at room temperature for 24 h in deionized water. A conductance meter (Mettler Toledo, Switzerland) was used to measure the initial electrical conductivity (EC1). The samples were boiled for 30 min before being analyzed. The system was allowed to cool to ambient temperature before the second electrical conductivity test (EC2) was conducted. Electrolyte leakage (%) was calculated using the formula:

EL (%) = (EC1 / EC2) × 100.

### 2.5 Malondialdehyde (MDA) content

Malondialdehyde (MDA) concentration was determined using the thiobarbituric acid (TBA) assay, following a modified method of Wantat et al. (2021). Leaf tissue (0.3 g) was homogenized using a mortar and pestle in 5 mL of 0.1 % (*w*/*v*) trichloroacetic acid (TCA). The TCA solution was freshly prepared by dissolving analytical-grade TCA in distilled water and was kept on ice throughout the extraction process. Following a 15-min centrifugation at 12,000 ×*g*, the supernatant was combined with 0.5 % (w/v) TBA. After measuring absorbance at 532 nm and correcting for nonspecific absorption at 600 nm using a microplate reader (Multiskan SkyHigh Microplate, Thermo Scientific, MA, USA), MDA content was analyzed using an extinction coefficient of 155 mM^−1^ cm^−1^ and expressed on a fresh weight basis (nmol g^−1^ FW).

### 2.6 2,2-Diphenyl-1-picrylhydrazyl radical (DPPH) radical scavenging activity

The DPPH assay was modified from the method described by Wantat et al. (2021). Holy basil leaf tissue (0.5 g) was homogenized in chilled 80 % ethanol and centrifuged at 12,000 ×*g*. The reaction mixture consisted of 0.2 mM DPPH solution and 20 μL of the extract, and was incubated in complete darkness at 25 °C for 20 min. Absorbance (Abs) at 520 nm was measured, using 80 % ethanol as both the control and the blank. DPPH radical inhibition (%) was then calculated as follows:

DPPH inhibition (%) = [(Abs.control - Abs.sample) / (Abs.control)] × 100.

### 2.7 Volatile oil compounds analysis

Sample preparation: 10 mL methanol was added to holy basil frozen powder prepared with liquid nitrogen (1 g). Then, the frozen powder in methanol was mixed by a vortex. The extraction was then performed by using a sonicator at 35–40 °C and centrifuged at 10,000 ×*g* to eliminate solids. This protocol was established through preliminary optimization to ensure high extraction efficiency and reproducibility for holy basil and has been previously reported by Chutimanukul, Jindamol, et al. (2022). The resulting supernatant was stored at −20 °C for further analysis.

Instrumentation: The analysis of VOCs was performed using a gas chromatograph (Agilent 7890B) coupled to a quadrupole time-of-flight mass spectrometer (GC/Q-TOF, Agilent 7250) and equipped with a PAL autosampler system (CTC Analytics AG, Switzerland). A 1-μL aliquot of the extract was injected through a multimode inlet (MMI) operated in splitless mode, with the inlet temperature maintained at 250 °C. Gas chromatography was performed on a DB-FFAP column (30 m × 0.25 mm × 0.25 μm, Agilent Technologies, USA) using high-purity helium (>99.999 %) as the carrier gas at a flow rate of 1.0 mL/min. The oven temperature was programmed to rise from 60 °C to 250 °C. For mass spectrometry, the transfer line was maintained at 250 °C, and ionization was performed by electron ionization (EI) at 70 eV. The mass detector operated in full scan mode, acquiring ions in the 20–350 amu range at 5 spectra per second. Quantitative data were processed with MassHunter software (version B0.4, Agilent Technologies, USA).

Targeted quantitation: Linalool, β-caryophyllene, humulene, methyl eugenol, and eugenol were selected as target compounds because they are the major volatile constituents of holy basil and key contributors to its aroma, whereas other volatiles occur at trace levels with inconsistent detectability (Chutimanukul, Wanichananan, et al., 2022). Calibration curves were generated using mixtures of five target standard compounds at concentrations ranging from 1.96 × 10^−1^ M to 6.48 × 10^−1^ M, with γ-hexalactone (at 1.75 × 10^−4^ M) serving as an internal standard. Compound identities were confirmed by their retention times and characteristic ions. As shown in Table 1, the chosen quantifier and qualifier ions were analyzed using Agilent MassHunter software (version B0.4, Agilent Technologies, USA). The results were exported to Microsoft Excel, processed, and expressed on a fresh weight basis (mg g^−1^).

**Table 1:** The selected quantifier and qualifier ions.

Retention time	Target compound	Quantifier	Qualifier	
			1	2
8.447	Linalool	71.0494	55.0545	93.0701
9.133	β-Caryophyllene	91.0552	79.0524	105.0679
9.994	Humulene	93.0701	91.0545	105.0701
13.662	Methyl eugenol	107.0471	91.0523	178.0963
15.147	Eugenol	164.0846	77.0367	103.0522

### 2.8 Statistical and data analysis

The experiment was conducted using a randomized complete block design (RCBD). For each time point and treatment, a minimum of three biological replicates was included, with each replicate consisting of at least three packets. All data, except for volatile oil composition, were analyzed using three-way ANOVA with Bonferroni correction to adjust for multiple comparisons. Although the senescence symptom scores were obtained using a 5-point scale, the scores were treated as continuous variables, which is a common practice in sensory and postharvest studies, and were analyzed using three-way ANOVA. Volatile oil composition data were analyzed separately using a two-way ANOVA with replication. SPSS software (version 29.0.1) was used for statistical analyses. Duncan's multiple range test (DMRT) was applied for mean separation, and differences were considered significant at *p* < 0.05.

## 3 Results

### 3.1 Effects of SNP on weight loss of holy basil grown under white and high blue light conditions

Weight loss was significantly influenced by light spectrum, SNP concentration, and storage duration, with notable interaction effects (Table 2). The three-way ANOVA revealed that light spectrum (F(1, 64) = 18.639, *p* < 0.001), SNP concentration (F(3, 64) = 98.063, *p* < 0.001), and day (F(3, 64) = 2488.439, *p* < 0.001) each had significant main effects on weight loss. All two-way interactions were also significant: light × SNP (F(3, 64) = 18.157, *p* < 0.001), light × day (F(3, 64) = 6.565, *p* < 0.001), and SNP × day (F(9, 64) = 27.702, *p* < 0.001). Importantly, a significant three-way interaction was observed among light, SNP, and day (F(9, 64) = 4.681, *p* < 0.001), indicating that the effect of SNP treatment on weight loss varied depending on both the light condition during cultivation and the day of measurement. The overall model exhibited high explanatory power (R^2^ = 0.992; adjusted R^2^ = 0.988). Detailed statistics are provided in Supplementary Document S2.

**Table 2:** Three-way ANOVA for indicators of holy basil responses under the combined effects of light spectrum (Light), SNP concentration (SNP), and storage duration (Day).

Factors	Weight loss	Senescence symptom score	Electrolyte leakage	MDA content	DPPH inhibition
Light	***	ns	ns	**	***
SNP	***	ns	***	***	***
Day	***	***	***	***	***
Light * SNP	***	ns	***	ns	**
Light * Day	***	ns	**	***	***
SNP * Day	***	ns	***	***	***
Light * SNP * Day	***	ns	ns	**	*

Note: *, **, and *** indicate significance levels at *p* ≤ 0.05, *p* ≤ 0.01, and *p* ≤ 0.001, respectively; ns denotes that differences were not significant.

**Fig. 1:**
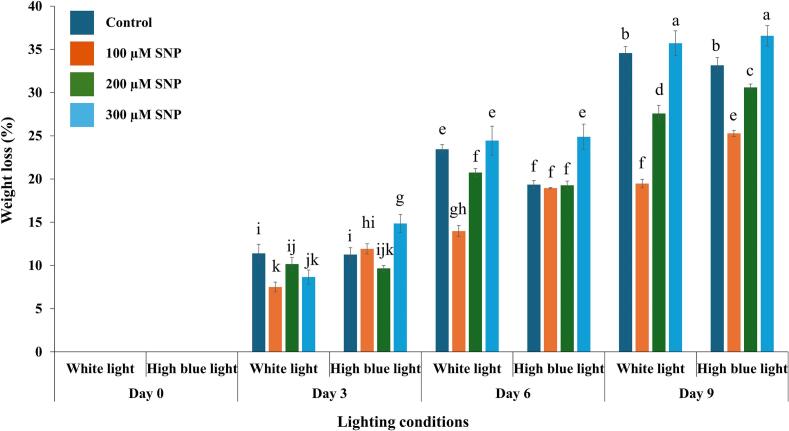
Effects of SNP on weight loss of holy basil grown under white and high blue light conditions. The bars represent the standard error (SE), with values expressed as mean ± SE (n = 3). Distinct letters indicate significant differences among treatments according to DMRT at *p* < 0.05. (For interpretation of the references to colour in this figure legend, the reader is referred to the web version of this article.)

Fresh weight loss of holy basil increased progressively during the nine-day storage period across all treatments. However, samples treated with SNP consistently exhibited lower weight loss than the control, particularly under high blue light conditions (Fig. 1). This pattern indicates that SNP treatment was associated with improved retention of fresh weight during cold storage.

Holy basil cultivated under white light conditions and treated with 100 μM SNP exhibited the most effective weight retention, showing lower weight loss after being stored at 10 °C for 6 d. Holy basil grown under white and high blue light and treated with 100 μM SNP showed lower weight loss than other treatments on day 9 of cold storage, whereas the 300 μM SNP-treated samples cultivated under high blue light conditions exhibited comparatively higher weight loss beginning on day 3 of the storage.

### 3.2 Effects of SNP on membrane stability of holy basil grown under white and high blue light conditions

**Fig. 2:**
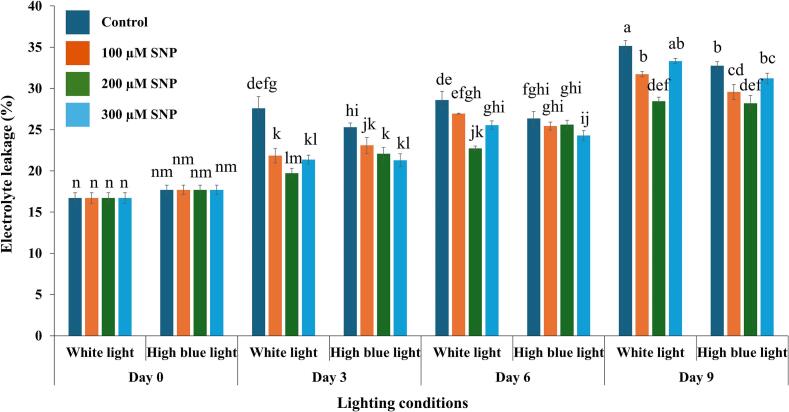
Effects of SNP on electrolyte leakage of holy basil grown under white and high blue light conditions. The bars represent the standard error (SE), with values expressed as mean ± SE (*n* = 3). Distinct letters indicate significant differences among treatments according to DMRT at *p* < 0.05. (For interpretation of the references to colour in this figure legend, the reader is referred to the web version of this article.)

Membrane stability was indirectly evaluated by measuring the percentage of electrolyte leakage (EL). Both SNP treatment and storage duration had significant effects on EL, along with notable two-way interactions among light, SNP concentration, and storage duration. However, the three-way interaction (light × SNP × day) was not statistically significant (F(9, 64) = 1.44,*p* = 0.192), indicating that the combined influence of light and SNP on EL did not vary significantly across storage days (Table 2; see Supplementary Document S2). Both the light spectrum and SNP treatments influenced EL in holy basil (Fig. 2). Pre-harvest exposure to white light and high blue light exhibited similar trends in EL values throughout the storage period. Basil grown under white and high blue light and treated with SNP showed lower EL on days 3, 6, and 9 compared to untreated controls. Noticeably, the postharvest application of 200 μM SNP resulted in the lowest EL values on day 9 in holy basil grown under both white and high blue light conditions.

### 3.3 Effects of SNP on the visual senescence symptom score of holy basil grown under white and high blue light conditions

The senescence symptom score is a visual indicator used to assess the extent of quality deterioration during storage. Holy basil treated with 200 μM SNP exhibited the lowest senescence symptom score under high blue light conditions on day 9 of storage (Table 3). Storage duration had a significant effect on senescence symptom scores throughout the storage period (*p* ≤ 0.001; Table 2; Supplementary Document S2).

**Table 3:** Effects of SNP on senescence symptom score of holy basil grown under white and high blue light conditions.

Storage (Days)	SNP treatment	Senescence symptom score	
		Whight light	High blue light
0	0 μM SNP	1.00d	1.00d
	100 μM SNP	1.00d	1.00d
	200 μM SNP	1.00d	1.00d
	300 μM SNP	1.00d	1.00d
3	0 μM SNP	1.67 ± 0.33c	1.00d
	100 μM SNP	1.67 ± 0.33c	1.67 ± 0.33c
	200 μM SNP	1.67 ± 0.33c	1.33 ± 0.33 cd
	300 μM SNP	1.67 ± 0.33c	1.67 ± 0.33c
6	0 μM SNP	2.67 ± 0.33b	2.00c
	100 μM SNP	3.00b	3.00b
	200 μM SNP	2.67 ± 0.33b	2.33 ± 0.33b
	300 μM SNP	2.67 ± 0.33b	1.67 ± 0.33c
9	0 μM SNP	4.00 ± 0.58a	3.67 ± 0.66ab
	100 μM SNP	3.67 ± 0.33a	3.33 ± 0.33ab
	200 μM SNP	3.33 ± 0.33a	3.00b
	300 μM SNP	3.67 ± 0.33a	3.67 ± 0.33a

Note: The values expressed as mean ± SE (n = 3). Distinct letters indicate significant differences among treatments according to DMRT at *p* < 0.05.

In contrast, no significant two-way interactions (light × SNP, light × day, or SNP × day) or three-way interaction (light × SNP × day) were detected (F(9, 64) = 0.19, *p* = 0.995), indicating that the effects of light conditions and SNP treatment on senescence progression were consistent over time (Table 2; Supplementary Document S2). Despite the absence of significant interaction effects, treatment with 200 μM SNP effectively delayed visual senescence and contributed to an extended shelf life of holy basil cultivated under high blue light conditions.

### 3.4 Effects of SNP on lipid peroxidation of holy basil grown under white and high blue light conditions

Membrane damage was assessed by quantifying lipid peroxidation through malondialdehyde (MDA) content, a reliable marker of oxidative degradation of unsaturated fatty acids. The three-way ANOVA revealed that light spectrum (F(1, 64) = 7.364, *p* = 0.009), SNP treatment (F(3, 64) = 295.676, *p* < 0.001), and storage duration (F(3, 64) = 1159.006, *p* < 0.001) each had statistically significant main effects on MDA levels. While the interaction between light × SNP was not significant (*p* = 0.910), both the light × day (F(3, 64) = 7.060, *p* < 0.001) and SNP × day (F(9, 64) = 103.038, *p* < 0.001) interactions were significant. Importantly, the three-way interaction among light, SNP, and day was also significant (F(9, 64) = 3.159, *p* = 0.003), indicating that the influence of SNP on lipid peroxidation was modulated by both the preharvest light environment and storage duration. These results underscore the dynamic interplay between light conditions and NO treatment in regulating oxidative membrane damage during cold storage (Table 2, Supplementary Document S2).

**Fig. 3:**
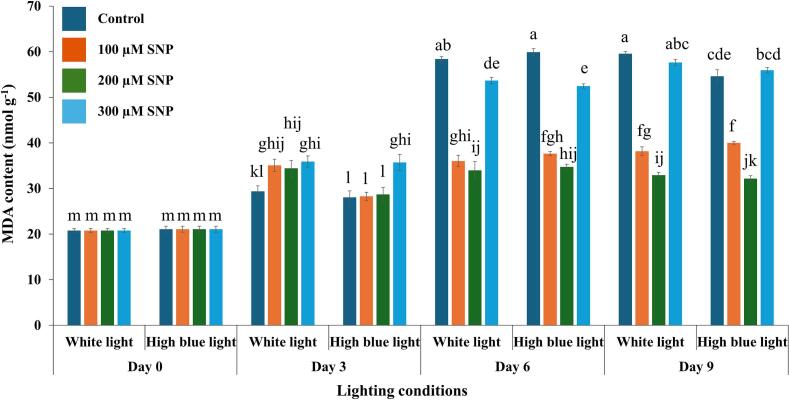
Effects of SNP on malondialdehyde content of holy basil grown under white and high blue light conditions. The bars represent the standard error (SE), with values expressed as mean ± SE (*n* = 3). Distinct letters indicate significant differences among treatments according to DMRT at *p* < 0.05. (For interpretation of the references to colour in this figure legend, the reader is referred to the web version of this article.)

On day 3, MDA content increased in the SNP treatments under white light. In contrast, under high blue light, 100 and 200 μM SNP treatments showed lower MDA levels than the 300 μM SNP treatment. Notably, the highest MDA levels remained in the control and 300 μM SNP treatments under both light conditions, while 200 μM SNP under high blue light consistently maintained the lowest MDA levels, followed by 100 μM SNP (Fig. 3).

### 3.5 Effects of SNP on antioxidant capacity of holy basil grown under white and high blue light conditions

**Fig. 4:**
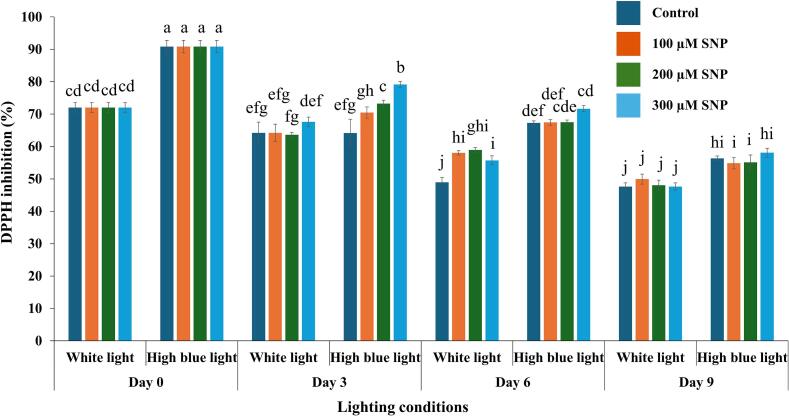
Effects of SNP on DPPH inhibition (%) of holy basil grown under white and high blue light conditions. The bars represent the standard error (SE), with values expressed as mean ± SE (*n* = 3). Distinct letters indicate significant differences among treatments according to DMRT at *p* < 0.05. (For interpretation of the references to colour in this figure legend, the reader is referred to the web version of this article.)

The variation in antioxidant capacity was shown in Fig. 4. Significant main effects of light treatment were detected by three-way ANOVA (F(1, 64) = 342.30, *p* < 0.001), SNP concentration (F(3, 64) = 8.15, *p* < 0.001), and storage day (F(3, 64) = 404.36, *p* < 0.001) on DPPH activity. All two-way interactions were also statistically significant: light × SNP (F(3, 64) = 4.99, *p* < 0.001), light × day (F(3, 64) = 27.04, *p* < 0.001), and SNP × day (F(9, 64) = 4.27, *p* < 0.001). Moreover, the three-way interaction among light, SNP, and day was significant (F(9, 64) = 2.49, *p* = 0.017), suggesting that the influence of SNP on antioxidant capacity varied depending on both the light condition and the storage duration. Overall, DPPH activity was primarily influenced by light treatment and sampling day, with SNP concentration having a smaller, yet statistically significant, impact (see Supplementary Document S2).

The results revealed that holy basil treated with 300 μM SNP and grown under high blue light conditions exhibited higher DPPH inhibition on days 3 and 6 compared to untreated plants, suggesting an enhanced ability to scavenge DPPH radicals. However, on day 9, the SNP's DPPH radical scavenging activity enhancement effect was no longer observed.

### 3.6 Effects of SNP on volatile compositions of holy basil grown under white and high blue light conditions

**Fig. 5:**
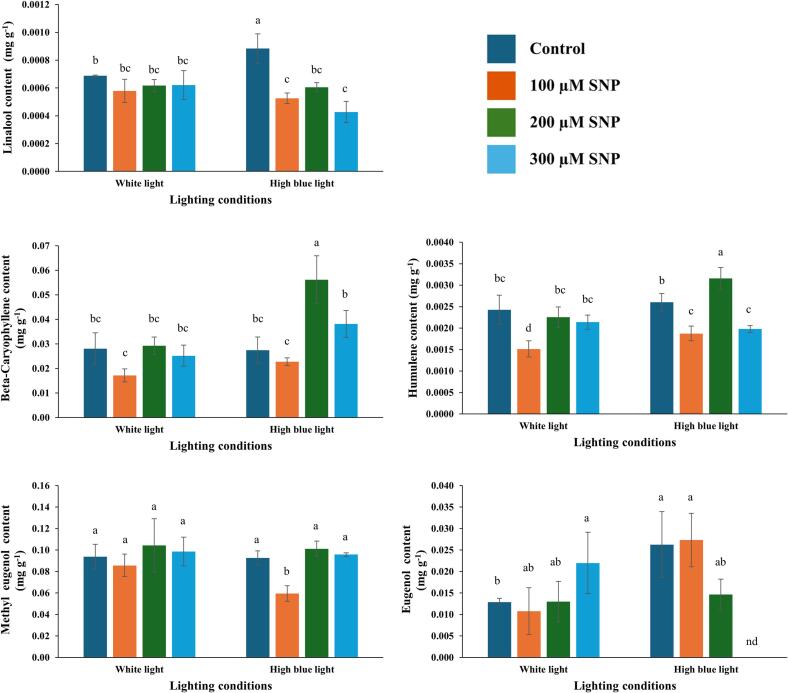
Effects of SNP on volatile oil compositions of holy basil grown under white and high blue light conditions. The bars represent the standard error (SE), with values expressed as mean ± SE (*n* = 3). Distinct letters indicate significant differences among treatments according to DMRT at *p* < 0.05, nd indicates not detected. (For interpretation of the references to colour in this figure legend, the reader is referred to the web version of this article.)

After storage of holy basil at 10 °C, the six-day sampling point was selected based on preliminary observations showing that samples stored up to day 6 exhibited clearly observable changes compared with earlier storage days, including visible deterioration and noticeable changes in aroma, indicating the onset of senescence and quality loss. Therefore, day 6 was chosen as a representative sampling point to evaluate treatment effects before severe deterioration occurred. Three major chemical groups were identified: monoterpenes (linalool), sesquiterpenes (β-caryophyllene and humulene), and phenylpropanoids (eugenol and methyl eugenol), as illustrated in Fig. 5. The R^2^ values of the calibration curves and representative GC–QTOF chromatograms are provided in Supplementary Document S3.

To assess the effects of light exposure and SNP treatment on volatile oil composition, a two-way ANOVA was conducted. For linalool, β-caryophyllene, humulene, and methyl eugenol, the interaction between light spectrum and SNP treatment showed a marginal trend but did not reach statistical significance (Table 4, Supplementary Document S2). In contrast, eugenol exhibited a significant interaction effect (F(3, 16) = 4.588, *p* = 0.017). These findings indicate that although light and SNP treatments individually did not significantly affect eugenol levels, their combined interaction contributed substantially to the variance observed in eugenol accumulation.

**Table 4:** Two-way ANOVA for the volatile oil compositions of holy basil under the combined effects of light spectrum (Light) and SNP concentration (SNP).

Factors	Linalool	β-Caryophyllene	Humulene	Methyl eugenol	Eugenol
Light	ns	**	ns	ns	ns
SNP	**	**	***	ns	ns
Light * SNP	ns	ns	ns	ns	*

Note: *, **, and *** indicate significance levels at *p* ≤ 0.05, *p* ≤ 0.01, and *p* ≤ 0.001, respectively; ns denotes that differences were not significant.

Consistent with the ANOVA results, the data indicated that holy basil grown under high blue light conditions had the highest linalool content. However, SNP treatments reduced linalool levels during cold storage. In contrast, basil grown under high blue light and treated with 200 μM SNP exhibited the highest levels of β-caryophyllene and humulene. Additionally, basil grown under both white and high blue light conditions and treated with 200 or 300 μM SNP maintained methyl eugenol levels comparable to the untreated control. Eugenol content did not differ among light and SNP treatments; however, it was undetectable in basil grown under high blue light and treated with 300 μM SNP.

## 4 Discussion

Weight loss and chilling injury (CI) are major physiological constraints that shorten the shelf life and reduce the market value of holy basil. Therefore, preserving fresh weight and preventing damage from low-temperature storage are critical quality attributes from both commercial and consumer perspectives. In this study, we demonstrate that specific light spectra during cultivation, in combination with postharvest sodium nitroprusside (SNP) treatment, significantly influenced weight loss during cold storage. Holy basil grown under white light and treated with 100 μM SNP exhibited the greatest reduction in weight loss after nine days of storage, while plants cultivated under high blue light and treated with either 100 or 200 μM SNP also showed improved weight retention compared with untreated controls. The reduced weight loss observed in SNP-treated samples is likely associated with the physiological role of nitric oxide (NO) in mitigating postharvest water loss. SNP-derived NO has been reported to regulate stomatal aperture, promoting partial stomatal closure under stress conditions and thereby reducing transpirational water loss (Garcia-Mata & Lamattina, 2001). In addition, NO has been shown to maintain cell membrane integrity, limit oxidative damage, and delay tissue senescence, all of which contribute to improved water retention during storage (Zhu et al., 2022). These combined effects provide a plausible explanation for the lower weight loss observed in SNP-treated basil. A significant three-way interaction among light spectrum, SNP concentration, and storage duration was detected, indicating that the effectiveness of SNP in reducing weight loss is not independent but is strongly influenced by preharvest light conditions and postharvest storage time. This interaction highlights the importance of integrating cultivation light strategies with postharvest NO-based treatments to optimize the shelf life and quality of holy basil.

Holy basil treated with 200 μM SNP exhibited the lowest senescence symptom scores under high blue light conditions on day 9 of storage, indicating that this treatment effectively extended shelf life. This finding is consistent with Bai et al. (2024), who reported delayed senescence in SNP-treated cowpeas, as evidenced by improved firmness and reduced postharvest weight loss. Similarly, Han et al. (2024) demonstrated that SNP application helped maintain quality in hyacinth beans, while Khaliq et al. (2021) showed that SNP significantly reduced anthracnose incidence by suppressing disease development and severity in custard apple during storage. Overall, these studies support the present results and highlight the positive role of NO in delaying senescence and preserving the postharvest quality of holy basil grown under high blue light conditions. The observed complexity suggests that photoperiod-dependent physiological mechanisms may modulate the plant's responsiveness to SNP over time, influencing water retention and senescence processes. The combination of pre-harvest light exposure (white or high blue light) and post-harvest SNP treatment likely acted synergistically — the light treatments primed the plants' defense systems, while SNP reinforced membrane stability during cold storage. This synergy may explain why basil exposed to these combined treatments exhibited reduced weight loss compared to controls. Likewise, Bai et al. (2024) observed that while weight loss in cowpeas increased over time, it remained lower in SNP-treated samples, particularly in those treated with 300 μM SNP demonstrating the potential of SNP across various crops. The reduction in water loss due to SNP treatment may also contribute to decreased CI.

Hydrogen peroxide (H_2_O_2_) is a key mediator of oxidative stress that induces membrane lipid peroxidation (Biswas & Mano, 2021). Although it does not directly attack unsaturated fatty acids, H_2_O_2_ may undergo the Fenton reaction to produce highly reactive hydroxyl radicals (^•^OH), which in turn initiate and propagate lipid peroxidation. MDA serves as a common stress biomarker for assessing cell membrane damage and lipid peroxidation levels. Low temperatures induce oxidative stress by disrupting electron transport and enhancing reactive oxygen species (ROS) accumulation. Our results suggested that 200 μM SNP-treated holy basil grown under all light conditions could reduce MDA accumulation during storage. This protective effect was observed regardless of the pre-harvest light treatment, implying that NO's role in modulating oxidative stress is central to reducing lipid peroxidation by upregulating antioxidant defense enzymes (Liu et al., 2011). Our results indicate a clear dose-dependent effect of SNP on lipid peroxidation, as reflected by MDA levels. The persistence of high MDA in both the untreated control and the 300 μM SNP treatment suggests that either the absence of exogenous NO or an excessive concentration fails to confer protective effects against oxidative stress. In contrast, the 200 μM SNP treatment under high blue light appears to be the optimal condition, effectively suppressing MDA accumulation and thereby reducing membrane lipid peroxidation. These findings suggest that NO at moderate concentrations plays a protective role in mitigating oxidative damage, whereas excessive levels may become ineffective or even detrimental. Previous studies have consistently shown that exogenous NO, supplied as SNP, can reduce lipid peroxidation under stress conditions. Suppression of MDA accumulation has been reported in tea leaves treated with 500 μM SNP under low-temperature stress (Wang et al., 2021), in NO-treated Bermuda grass (Fan et al., 2015), broccoli treated with 200 μM SNP (Zou et al., 2025), and SNP-treated potatoes (Dai et al., 2023). Furthermore, Sharma and Sharma (2015) also found that increasing NO concentrations led to a minor rise in MDA levels in Japanese plum.

The cell membrane is the primary plant component affected by low temperatures, as cold conditions enhance membrane permeability, resulting in intracellular electrolyte leakage (EL) and potential cell damage. Venkatachalam and Meenune (2015) stated that the increase in EL might result from the loss of membrane integrity caused by lipoxygenase (LOX) enzyme. Our results showed the lowest EL in 200 μM SNP-treated holy basil plants on day 9. SNP may protect membrane integrity by maintaining fluidity, scavenging ROS, or altering phospholipid metabolism, which may explain the lower EL in treated samples (Gan et al., 2015). Similarly, Duan et al. (2007) found that the increase in EL, potentially linked to LOX activity, was inhibited by NO; however, the extent of this inhibition appeared to depend on the concentration of SNP. The observed damage could be attributed to low temperatures increasing H_2_O_2_ and superoxide anion radical levels, which stimulate MDA production and disrupt the leaf cell membrane structure, resulting in EL. As a signaling molecule, NO plays a critical role in reducing MDA accumulation and membrane peroxidation, thereby preserving cytomembrane structure and mitigating damage under cold stress (Wang et al., 2021). Furthermore, SNP treatment reduced EL in potato compared with the control, indicating that 200 μM SNP can limit the increase in EL (Dai et al., 2023).

DPPH inhibition was used as an indicator of non-enzymatic antioxidant activity in holy basil leaves. A continuous decline in DPPH inhibition was observed throughout storage, which is commonly associated with metabolic slowdown, oxidative degradation of bioactive compounds, and senescence processes (Pérez-Lamela et al., 2021). However, treatments involving optimized light spectra combined with SNP partially mitigated this decline by maintaining relatively higher antioxidant activity during the first six days of storage, thereby helping to limit ROS-induced oxidative damage. In a previous study, Chutimanukul, Wanichananan, et al. (2022) demonstrated that the ratio of light spectra affected antioxidant capacity, leading to elevated total phenolic content and greater DPPH inhibition in postharvest holy basil when exposed to a 1 red: 3 blue LED light spectrum ratio. Similarly, Zou et al. (2025) reported that the exogenous application of 200 μM SNP enhanced the total antioxidant capacity of broccoli samples. Additionally, strawberry fruit treated with SNP exhibited the highest DPPH activity (Gautam et al., 2024). While 200 μM SNP was the most effective in mitigating CI, 300 μM SNP led to the highest accumulation of antioxidant levels. This suggests a potential trade-off between extending shelf life and enhancing nutritional quality. Therefore, selecting the optimal SNP concentration should be guided by the primary goal of the postharvest treatment whether it prioritizes visual quality and longevity or nutritional enhancement.

The essential oil of holy basil contains more than 30 secondary metabolites, predominantly phenolics and terpenoids, which are synthesized via the phenylpropanoid and mevalonate pathways, respectively (Chutimanukul, Jindamol, et al., 2022; Tangpao et al., 2022). After storing holy basil leaves at 10 °C for six days, three primary volatile compounds were identified: monoterpenes (linalool), sesquiterpenes (β-caryophyllene and humulene), and phenylpropanoids (eugenol and methyl eugenol). Phenylpropanoids are synthesized via the shikimate and phenylpropanoid pathways. According to Ge et al. (2019), SNP treatment in apple fruit significantly enhanced phenylalanine ammonia-lyase (PAL), cinnamate 4-hydroxylase (C4H), and 4-coumarate: coenzyme A ligase (4CL) activities, leading to a greater accumulation of phenolic compounds, flavonoids, and lignin. These findings suggest a strong association between the activation of the phenylpropanoid pathway and metabolite accumulation in the shikimate pathway. The highest levels of β-caryophyllene and humulene were found in basil grown under high blue light conditions and treated with 200 μM SNP. The fact that the highest levels of β-caryophyllene and humulene were observed in plants grown under high blue light and treated with 200 μM SNP suggests a synergistic effect. Light priming may enhance metabolic readiness, while NO from SNP amplifies the metabolic flux through terpenoid biosynthetic pathways. β-Caryophyllene is a sesquiterpene known not only for its antioxidant and anti-inflammatory activities but also for its involvement in plant defense responses under abiotic stress. Wu et al. (2019) reported that caryophyllene levels increased in papaya peel under chilling stress, suggesting its potential role in membrane stabilization and ROS scavenging. Therefore, the elevated β-caryophyllene content observed in this study may reflect a protective mechanism induced by the combined pre-harvest blue light exposure and SNP treatment, which enhances tolerance to chilling stress during storage.

Holy basil grown under white light condition and treated with SNP did not show any difference in linalool content. However, a reduction in linalool was observed in SNP-treated holy basil grown under high blue light, which may result from metabolic pathway balance regulation. Decreased expression of enzymes involved in linalool biosynthesis, along with competition from the phenylpropanoid pathway, possibly due to light-induced shifts in metabolic flux between terpenoid and phenylpropanoid pathways (Das & Prakash, 2024; Gurav, Dholakia and Giri, 2022). Interestingly, linalool, the dominant volatile compound in papaya, was found by Wu et al. (2019) to decrease markedly under chilling storage, indicating that its biosynthesis is inhibited by low temperature. This reduction may contribute to the loss of aroma and quality typically observed in chilling-sensitive fruits. In the present study, the decline in linalool in blue-light–grown, SNP-treated basil might therefore reflect a metabolic adjustment, where precursors are redirected toward the production of other stress-responsive volatiles such as β-caryophyllene and humulene, which may provide greater protection against cold-induced oxidative damage.

Eugenol and methyl eugenol were also unaffected by light quality and SNP treatments. These findings highlight the role of specific light spectrum ratios and SNP treatment in reducing cellular damage by enhancing antioxidant properties, VOC accumulation, and the preservation of holy basil quality during low-temperature storage.

Although this study focused on major volatile oil constituents, the combined application of pre-harvest light spectrum manipulation and postharvest SNP treatment may also influence other biochemical components of holy basil, including stress-related metabolites and non-volatile secondary metabolites; however, these effects were not directly assessed. The proposed approach has limitations, as SNP efficacy is concentration-dependent and excessive nitric oxide may induce undesirable physiological responses, and the targeted analysis of selected volatiles over a limited storage period may not fully capture quality changes during extended storage. Compared with conventional preservation strategies such as modified atmosphere packaging or edible coatings, the light–SNP treatment represents a complementary, physiologically driven approach that integrates pre-harvest and postharvest management to modulate antioxidant status and volatile metabolism.

Taken together, our findings highlight the novelty and strength of this study in demonstrating the synergistic interaction between nitric oxide (NO), released from SNP, and pre-harvest blue light in enhancing postharvest cold-stress tolerance of holy basil. Unlike previous studies that investigated NO or light treatments independently, this work integrates pre-harvest light priming with postharvest NO application, providing a more comprehensive understanding of how membrane stability, antioxidant defense, and secondary metabolism are coordinately regulated. This combined approach offers a practical and effective strategy for improving postharvest quality and extending the storage life of holy basil under cold conditions.

## 5 Conclusion

The present study demonstrates that the combined application of high-intensity blue light during cultivation and postharvest SNP treatment effectively alleviates chilling injury in holy basil during cold storage. This integrated strategy enhanced membrane integrity, suppressed oxidative stress, and preserved antioxidant capacity and key volatile oil components. In particular, the combination of blue light preconditioning with 200 μM SNP was the most effective treatment for maintaining postharvest quality and extending storage life under chilling conditions. These findings highlight the potential of integrating light management and NO-based postharvest treatments as a practical approach for improving the cold storage performance of holy basil. Further studies focusing on NO-mediated signaling pathways, molecular mechanisms, and commercial-scale validation would support broader application in medicinal plant supply chains.

## CRediT authorship contribution statement

**Thanaboon Plakunmonthon:** Writing – original draft, Methodology, Investigation, Formal analysis, Conceptualization. **Panita Chutimanukul:** Writing – review & editing, Methodology. **Kenji Matsui:** Writing – review & editing, Methodology. **Kanogwan Seraypheap:** Writing – review & editing, Supervision, Project administration, Methodology, Conceptualization.

## Declaration of competing interest

The authors declare that they have no known competing financial interests or personal relationships that could have appeared to influence the work reported in this paper.

## Data Availability

Data will be made available on request.

## Appendix A Supplementary data

Supplementary material Image 1

## References

[bb0005] Bai C., Zhang F., Meng D., Watkins C.B., Ma L., Fu A., Sang Z., Guo S., Wang H., Wang Q., Zuo J., Zheng Y. (2024). Sodium nitroprusside (SNP) generated nitric oxide delays senescence of cowpea (*Vigna unguiculata* (L.) Walp). Postharvest Biology and Technology.

[bb0010] Biswas M.S., Mano J.I. (2021). Lipid peroxide-derived reactive carbonyl species as mediators of oxidative stress and signaling. Frontiers in Plant Science.

[bb0015] Chutimanukul P., Jindamol H., Thongtip A., Korinsak S., Romyanon K., Toojinda T., Chutimanukul P. (2022). Physiological responses and variation in secondary metabolite content among Thai holy basil cultivars (*Ocimum tenuiflorum* L.) grown under controlled environmental conditions in a plant factory. Frontiers in Plant Science.

[bb0020] Chutimanukul P., Wanichananan P., Janta S., Toojinda T., Darwell C.T., Mosaleeyanon K. (2022). The influence of different light spectra on physiological responses, antioxidant capacity and chemical compositions in two holy basil cultivars. Scientific Reports.

[bb0025] Cozzolino R., Pace B., Cefola M., Martignetti A., Stocchero M., Fratianni F., Naazzaro F., De Giulio B. (2016). Assessment of volatile profile as potential marker of chilling injury of basil leaves during postharvest storage. Food Chemistry.

[bb0030] Dai Y., Zhang Y., Wang W., Zhang Y. (2023). The effect of sodium nitroprusside treatment on storage ability of fresh-cut potato. Foods.

[bb0035] Das S., Prakash B., Prakash B., Dubey N.K., Brilhante F., de São José J. (2024). Plant Essential Oils.

[bb0040] Duan X., Su X., You Y., Qu H., Li Y., Jiang Y. (2007). Influence of the nitric oxide donor, sodium nitroprusside, on lipid peroxidation and antioxidant activity in pericarp tissue of longan fruit. The Journal of Horticultural Science and Biotechnology.

[bb0045] Fan J., Chen K., Amombo E., Hu Z., Chen L., Fu J. (2015). Physiological and molecular mechanism of nitric oxide (NO) involved in bermudagrass response to cold stress. PLoS ONE.

[bb0050] Farouk S., Al-Huqail A.A. (2020). Sodium nitroprusside application regulates antioxidant capacity, improves phytopharmaceutical production and essential oil yield of marjoram herb under drought. Industrial Crops and Products.

[bb0055] Gan L., Wu X., Zhong Y. (2015). Exogenously applied nitric oxide enhances the drought tolerance in hulless barley. Plant Production Science.

[bb0060] Garcia-Mata C., Lamattina L. (2001). Nitric oxide induces stomatal closure and enhances the adaptive plant responses against drought stress. Plant Physiology.

[bb0065] Gautam A., Gill P.P.S., Singh N., Jawandha S.K., Arora R., Singh A., Ajay A. (2024). Composite coating of xanthan gum with sodium nitroprusside alleviates the quality deterioration in strawberry fruit. Food Hydrocolloids.

[bb0070] Ge Y., Chen Y., Li C., Zhao J., Wei M., Li X., Yang S., Mi Y. (2019). Effect of sodium nitroprusside treatment on shikimate and phenylpropanoid pathways of apple fruit. Food Chemistry.

[bb0075] Gohari G., Alavi Z., Esfandiari E., Panahirad S., Hajihoseinlou S., Fotopoulos V. (2020). Interaction between hydrogen peroxide and sodium nitroprusside following chemical priming of *Ocimum basilicum* L. against salt stress. Physiologia Plantarum.

[bb0080] Gurav T.P., Dholakia B.B., Giri A.P. (2022). A glance at the chemodiversity of Ocimum species: Trends, implications, and strategies for the quality and yield improvement of essential oil. Phytochemistry Reviews.

[bb0085] Han L., Wang Z., Watkins C.B., Ma L., He X., Bai C., Zheng Y. (2024). The regulatory mechanisms of delayed senescence of nitric oxide treatment of hyacinth beans. Postharvest Biology and Technology.

[bb0090] Herman A., Tambor K., Herman A. (2016). Linalool affects the antimicrobial efficacy of essential oils. Current Microbiology.

[bb0095] Jensen N.B., Clausen M.R., Kjaer K.H. (2018). Spectral quality of supplemental LED grow light permanently alters stomatal functioning and chilling tolerance in basil (*Ocimum basilicum* L.). Scientia Horticulturae.

[bb0100] Jiang Y., Huang B. (2002). Protein alterations in tall fescue in response to drought stress and abscisic acid. Crop Science.

[bb0105] Khaliq G., Ullah M., Memon S.A., Ali A., Rashid M. (2021). Exogenous nitric oxide reduces postharvest anthracnose disease and maintains quality of custard apple (*Annona squamosa* L.) fruit during ripening. Journal of Food Measurement and Characterization.

[bb0110] Liu X., Wang L., Liu L., Guo Y., Ren H. (2011). Alleviating effect of exogenous nitric oxide in cucumber seedling against chilling stress. African Journal of Biotechnology.

[bb0115] Loi M., Villani A., Paciolla F., Mulè G., Paciolla C. (2020). Challenges and opportunities of light-emitting diode (LED) as key to modulate antioxidant compounds in plants. A review. Antioxidants.

[bb0120] Lu X., Yin F., Liu C., Liang Y., Song M., Shang F., Lui Y., Shuai L. (2023). Nitric oxide alleviates chilling injury in cucumber (*Cucumis sativus* L.) fruit by regulating membrane lipid and energy metabolism. International Journal of Food Properties.

[bb0125] Nabi R.B.S., Tayade R., Hussain A., Kulkarni K.P., Imran Q.M., Mun B.G., Yun B.W. (2019). Nitric oxide regulates plant responses to drought, salinity, and heavy metal stress. Environmental and Experimental Botany.

[bb0130] Naznin M.T., Lefsrud M., Gravel V., Azad M.O.K. (2019). Blue light added with red LEDs enhance growth characteristics, pigments content, and antioxidant capacity in lettuce, spinach, kale, basil, and sweet pepper in a controlled environment. Plants.

[bb0135] Patiño L.S., Castellanos D.A., Herrera A.O. (2018). Influence of 1-MCP and modified atmosphere packaging in the quality and preservation of fresh basil. Postharvest Biology and Technology.

[bb0140] Pérez-Lamela C., Franco I., Falqué E. (2021). Impact of high-pressure processing on antioxidant activity during storage of fruit and fruit products: A review. Molecules.

[bb0145] Rabara R.C., Behrman G., Timbol T., Rushton P.J. (2017). Effect of spectral quality of monochromatic LED lights on the growth of artichoke seedlings. Frontiers in Plant Science.

[bb0150] Rogerio A.P., Andrade E.L., Leite D.F., Figueiredo C.P., Calixto J.B. (2009). Preventive and therapeutic anti-inflammatory properties of the sesquiterpene α-humulene in experimental airways allergic inflammation. British Journal of Pharmacology.

[bb0155] Sale A.I., Uthairatanakij A., Laohakunjit N., Jitareerat P., Kaisangsri N. (2023). Pre-harvest supplemental LED treatments led to improved postharvest quality of sweet basil leaves. Journal of Photochemistry and Photobiology B: Biology.

[bb0160] Sharma A., Bhardwaj G., Sohal H.S., Gohain A. (2022). Nutraceuticals and Health Care.

[bb0165] Sharma S., Sharma R.R. (2015). Nitric oxide inhibits activities of PAL and PME enzymes and reduces chilling injury in ‘Santa Rosa’ Japanese plum (*Prunus salicina* Lindell). Journal of Plant Biochemistry and Biotechnology.

[bb0170] Tangpao T., Charoimek N., Teerakitchotikan P., Leksawasdi N., Jantanasakulwong K., Rachtanapun P., Sommano S.R. (2022). Volatile organic compounds from basil essential oils: Plant taxonomy, biological activities, and their applications in tropical fruit productions. Horticulturae.

[bb0175] Venkatachalam K., Meenune M. (2015). Effect of methyl jasmonate on physiological and biochemical quality changes of longkong fruit under low temperature storage. Fruit.

[bb0180] Wang Y., Yu Q., Li Y., Li J., Chen J., Liu Z., Eissa M.A. (2021). Mechanisms of nitric oxide in the regulation of chilling stress tolerance in *Camellia sinensis*. Horticulturae.

[bb0185] Wantat A., Rojsitthisak P., Seraypheap K. (2021). Inhibitory effects of high molecular weight chitosan coating on ‘Hom thong’ banana fruit softening. Food Packaging and Shelf Life.

[bb0190] Wu Q., Li Z., Chen X., Yun Z., Li T., Jiang Y. (2019). Comparative metabolites profiling of harvested papaya (*Carica papaya* L.) peel in response to chilling stress. Journal of the Science of Food and Agriculture.

[bb0195] Yang M., Lv Y., Tian X., Lou J., An R., Zhang Q., Dong Z. (2017). Neuroprotective effect of β-caryophyllene on cerebral ischemia-reperfusion injury via regulation of necroptotic neuronal death and inflammation: In vivo and in vitro. Frontiers in Neuroscience.

[bb0200] Zhu Y., Du M., Jiang X., Huang M., Zhao J. (2022). Nitric oxide acts as an inhibitor of postharvest senescence in horticultural products. International Journal of Molecular Sciences.

[bb0205] Zou Y., Sun L., Feng Z., Guan Z., Lv C., Wang J. (2025). Exogenous sodium nitroprusside treatment alleviates postharvest broccoli nutritional quality deterioration by mediating the ascorbic acid-glutathione cycle. Journal of Food Measurement and Characterization.

