# Supplementary material for: Integrated effects of pre-harvest high blue light and postharvest sodium nitroprusside on volatile oil composition and quality of cold-stored holy basil
Source: Food Chem X. 2026 Jan 5;33:103493. doi: 10.1016/j.fochx.2026.103493 (PMC12814088; doi:10.1016/j.fochx.2026.103493)
Supplement: Supplementary file 1 — Supplementary material [file mmc1.docx]

**Supplementary Document S1:**

**Pre-harvest blue light exposure and postharvest sodium nitroprusside application enhance chilling tolerance and improve storage quality of holy basil**

Thanaboon Plakunmonthon, Panita Chutimanukul, Kenji Matsui, and Kanogwan Seraypheap^*^

*Corresponding author: Kanogwan Seraypheap

Department of Botany, Faculty of Science, Tel.: +66-2-218-5495, Fax: +66-2-252-8979

E-mail: [kanogwan.k@chula.ac.th](mailto:kanogwan.k@chula.ac.th)


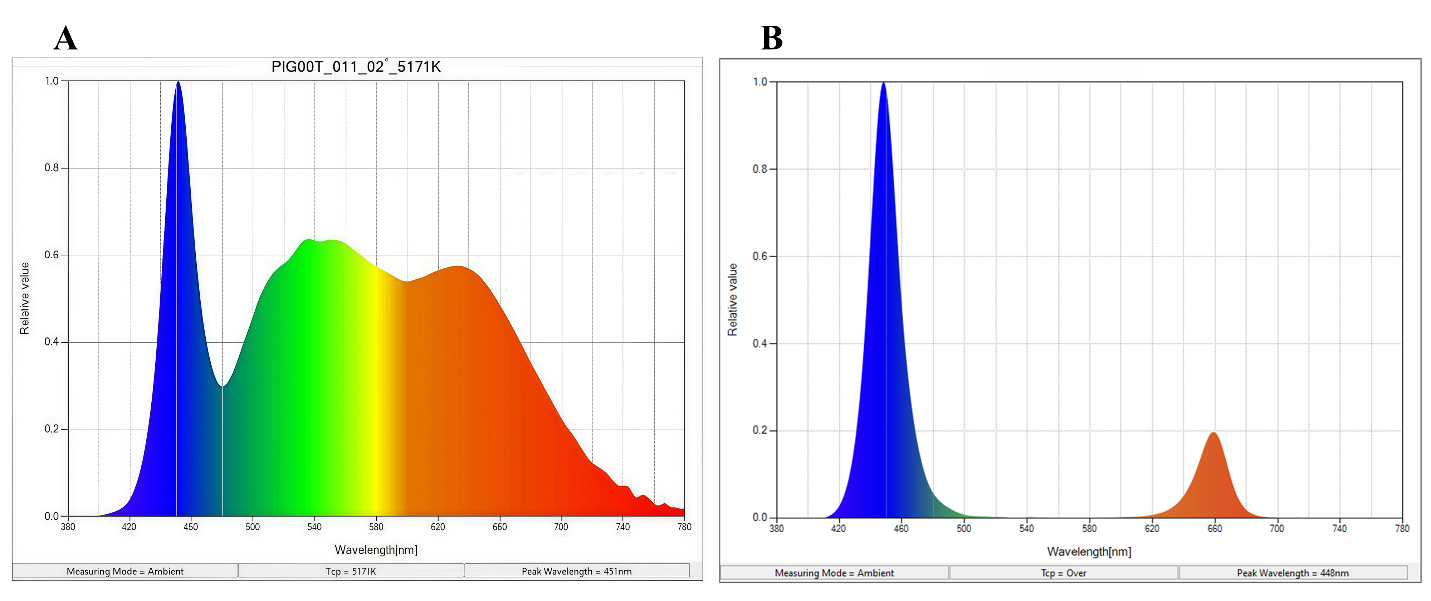


**Figure S1.** The relative spectrum of lighting conditions consists of white light (A) and high blue light (1Red:3Blue) (B) were measured using a light meter (Sekonic C-7000 Spectrometer, Japan).

**Supplementary Document S2:**

**Pre-harvest blue light exposure and postharvest sodium nitroprusside application enhance chilling tolerance and improve storage quality of holy basil**

Thanaboon Plakunmonthon, Panita Chutimanukul, Kenji Matsui, and Kanogwan Seraypheap^*^

*Corresponding author: Kanogwan Seraypheap

Department of Botany, Faculty of Science, Tel.: +66-2-218-5495, Fax: +66-2-252-8979

E-mail: [kanogwan.k@chula.ac.th](mailto:kanogwan.k@chula.ac.th)

**The results of the statistical analysis using a three-way ANOVA**

**Weight loss**

| Tests of Between-Subjects Effects | | | | | |
| --- | --- | --- | --- | --- | --- |
| Dependent Variable: Weight loss | | | | | |
| Source | Type III Sum of Squares | df | Mean Square | F | Sig. |
| Corrected Model | 13373.623^a^ | 31 | 431.407 | 262.702 | <.001 |
| Intercept | 22813.553 | 1 | 22813.553 | 13892.133 | <.001 |
| Light | 30.608 | 1 | 30.608 | 18.639 | <.001 |
| SNP | 483.113 | 3 | 161.038 | 98.063 | <.001 |
| Day | 12259.485 | 3 | 4086.495 | 2488.439 | <.001 |
| Light * SNP | 89.452 | 3 | 29.817 | 18.157 | <.001 |
| Light * Day | 32.343 | 3 | 10.781 | 6.565 | <.001 |
| SNP * Day | 409.432 | 9 | 45.492 | 27.702 | <.001 |
| Light * SNP * Day | 69.188 | 9 | 7.688 | 4.681 | <.001 |
| Error | 105.100 | 64 | 1.642 |  |  |
| Total | 36292.276 | 96 |  |  |  |
| Corrected Total | 13478.723 | 95 |  |  |  |
| a. R Squared = .992 (Adjusted R Squared = .988) | | | | | |

**Light * SNP**

| Pairwise Comparisons | | | | | | | |
| --- | --- | --- | --- | --- | --- | --- | --- |
| Dependent Variable: Weight loss | | | | | | | |
| SNP (µM) | (I) Light | (J) Light | Mean Difference (I-J) | Std. Error | Sig.^b^ | 95% Confidence Interval for Difference^b^ | |
|  |  |  |  |  |  | Lower Bound | Upper Bound |
| 0 | White light | High blue light | 1.410^*^ | .523 | .009 | .365 | 2.456 |
|  | High blue light | White light | -1.410^*^ | .523 | .009 | -2.456 | -.365 |
| 100 | White light | High blue light | -3.802^*^ | .523 | <.001 | -4.847 | -2.757 |
|  | High blue light | White light | 3.802^*^ | .523 | <.001 | 2.757 | 4.847 |
| 200 | White light | High blue light | -.255 | .523 | .628 | -1.300 | .790 |
|  | High blue light | White light | .255 | .523 | .628 | -.790 | 1.300 |
| 300 | White light | High blue light | -1.871^*^ | .523 | <.001 | -2.916 | -.825 |
|  | High blue light | White light | 1.871^*^ | .523 | <.001 | .825 | 2.916 |
| Based on estimated marginal means | | | | | | | |
| *. The mean difference is significant at the .05 level. | | | | | | | |
| b. Adjustment for multiple comparisons: Bonferroni. | | | | | | | |

| Univariate Tests | | | | | | |
| --- | --- | --- | --- | --- | --- | --- |
| Dependent Variable: Weight loss | | | | | | |
| SNP (µM) | | Sum of Squares | df | Mean Square | F | Sig. |
| 0 | Contrast | 11.936 | 1 | 11.936 | 7.268 | .009 |
|  | Error | 105.100 | 64 | 1.642 |  |  |
| 100 | Contrast | 86.743 | 1 | 86.743 | 52.821 | <.001 |
|  | Error | 105.100 | 64 | 1.642 |  |  |
| 200 | Contrast | .390 | 1 | .390 | .237 | .628 |
|  | Error | 105.100 | 64 | 1.642 |  |  |
| 300 | Contrast | 20.993 | 1 | 20.993 | 12.783 | <.001 |
|  | Error | 105.100 | 64 | 1.642 |  |  |
| Each F tests the simple effects of Light within each level combination of the other effects shown. These tests are based on the linearly independent pairwise comparisons among the estimated marginal means. | | | | | | |

| Pairwise Comparisons | | | | | | | |
| --- | --- | --- | --- | --- | --- | --- | --- |
| Dependent Variable: Weight loss | | | | | | | |
| Light | (I) SNP  (µM) | (J) SNP  (µM) | Mean Difference (I-J) | Std. Error | Sig.^b^ | 95% Confidence Interval for Difference^b^ | |
|  |  |  |  |  |  | Lower Bound | Upper Bound |
| White light | 0 | 100 | 7.119^*^ | .523 | <.001 | 5.695 | 8.544 |
|  |  | 200 | 2.731^*^ | .523 | <.001 | 1.307 | 4.156 |
|  |  | 300 | .152 | .523 | 1.000 | -1.273 | 1.576 |
|  | 100 | 0 | -7.119^*^ | .523 | <.001 | -8.544 | -5.695 |
|  |  | 200 | -4.388^*^ | .523 | <.001 | -5.813 | -2.964 |
|  |  | 300 | -6.968^*^ | .523 | <.001 | -8.392 | -5.543 |
|  | 200 | 0 | -2.731^*^ | .523 | <.001 | -4.156 | -1.307 |
|  |  | 100 | 4.388^*^ | .523 | <.001 | 2.964 | 5.813 |
|  |  | 300 | -2.579^*^ | .523 | <.001 | -4.004 | -1.155 |
|  | 300 | 0 | -.152 | .523 | 1.000 | -1.576 | 1.273 |
|  |  | 100 | 6.968^*^ | .523 | <.001 | 5.543 | 8.392 |
|  |  | 200 | 2.579^*^ | .523 | <.001 | 1.155 | 4.004 |
| High blue light | 0 | 100 | 1.907^*^ | .523 | .003 | .482 | 3.331 |
|  |  | 200 | 1.066 | .523 | .275 | -.359 | 2.490 |
|  |  | 300 | -3.129^*^ | .523 | <.001 | -4.554 | -1.705 |
|  | 100 | 0 | -1.907^*^ | .523 | .003 | -3.331 | -.482 |
|  |  | 200 | -.841 | .523 | .678 | -2.265 | .584 |
|  |  | 300 | -5.036^*^ | .523 | <.001 | -6.460 | -3.611 |
|  | 200 | 0 | -1.066 | .523 | .275 | -2.490 | .359 |
|  |  | 100 | .841 | .523 | .678 | -.584 | 2.265 |
|  |  | 300 | -4.195^*^ | .523 | <.001 | -5.620 | -2.771 |
|  | 300 | 0 | 3.129^*^ | .523 | <.001 | 1.705 | 4.554 |
|  |  | 100 | 5.036^*^ | .523 | <.001 | 3.611 | 6.460 |
|  |  | 200 | 4.195^*^ | .523 | <.001 | 2.771 | 5.620 |
| Based on estimated marginal means | | | | | | | |
| *. The mean difference is significant at the .05 level. | | | | | | | |
| b. Adjustment for multiple comparisons: Bonferroni. | | | | | | | |

| Univariate Tests | | | | | | |
| --- | --- | --- | --- | --- | --- | --- |
| Dependent Variable: Weight loss | | | | | | |
| Light | | Sum of Squares | df | Mean Square | F | Sig. |
| White light | Contrast | 397.876 | 3 | 132.625 | 80.761 | <.001 |
|  | Error | 105.100 | 64 | 1.642 |  |  |
| High blue light | Contrast | 174.689 | 3 | 58.230 | 35.459 | <.001 |
|  | Error | 105.100 | 64 | 1.642 |  |  |
| Each F tests the simple effects of SNP within each level combination of the other effects shown. These tests are based on the linearly independent pairwise comparisons among the estimated marginal means. | | | | | | |

**Light * Day**

| Pairwise Comparisons | | | | | | | |
| --- | --- | --- | --- | --- | --- | --- | --- |
| Dependent Variable: Weight loss | | | | | | | |
| Day | (I) Light | (J) Light | Mean Difference (I-J) | Std. Error | Sig.^b^ | 95% Confidence Interval for Difference^b^ | |
|  |  |  |  |  |  | Lower Bound | Upper Bound |
| Day 0 | White light | High blue light | -2.220E-16 | .523 | 1.000 | -1.045 | 1.045 |
|  | High blue light | White light | 2.220E-16 | .523 | 1.000 | -1.045 | 1.045 |
| Day 3 | White light | High blue light | -2.496^*^ | .523 | <.001 | -3.541 | -1.450 |
|  | High blue light | White light | 2.496^*^ | .523 | <.001 | 1.450 | 3.541 |
| Day 6 | White light | High blue light | .043 | .523 | .935 | -1.002 | 1.088 |
|  | High blue light | White light | -.043 | .523 | .935 | -1.088 | 1.002 |
| Day 9 | White light | High blue light | -2.065^*^ | .523 | <.001 | -3.110 | -1.019 |
|  | High blue light | White light | 2.065^*^ | .523 | <.001 | 1.019 | 3.110 |
| Based on estimated marginal means | | | | | | | |
| *. The mean difference is significant at the .05 level. | | | | | | | |
| b. Adjustment for multiple comparisons: Bonferroni. | | | | | | | |

| Univariate Tests | | | | | | |
| --- | --- | --- | --- | --- | --- | --- |
| Dependent Variable: Weight loss | | | | | | |
| Day | | Sum of Squares | df | Mean Square | F | Sig. |
| Day 0 | Contrast | 2.958E-31 | 1 | 2.958E-31 | .000 | 1.000 |
|  | Error | 105.100 | 64 | 1.642 |  |  |
| Day 3 | Contrast | 37.368 | 1 | 37.368 | 22.755 | <.001 |
|  | Error | 105.100 | 64 | 1.642 |  |  |
| Day 6 | Contrast | .011 | 1 | .011 | .007 | .935 |
|  | Error | 105.100 | 64 | 1.642 |  |  |
| Day 9 | Contrast | 25.573 | 1 | 25.573 | 15.572 | <.001 |
|  | Error | 105.100 | 64 | 1.642 |  |  |
| Each F tests the simple effects of Light within each level combination of the other effects shown. These tests are based on the linearly independent pairwise comparisons among the estimated marginal means. | | | | | | |

| Pairwise Comparisons | | | | | | | |
| --- | --- | --- | --- | --- | --- | --- | --- |
| Dependent Variable: Weight loss | | | | | | | |
| Light | (I) Day | (J) Day | Mean Difference (I-J) | Std. Error | Sig.^b^ | 95% Confidence Interval for Difference^b^ | |
|  |  |  |  |  |  | Lower Bound | Upper Bound |
| White light | Day 0 | Day 3 | -9.422^*^ | .523 | <.001 | -10.847 | -7.998 |
|  |  | Day 6 | -20.649^*^ | .523 | <.001 | -22.073 | -19.224 |
|  |  | Day 9 | -29.333^*^ | .523 | <.001 | -30.757 | -27.908 |
|  | Day 3 | Day 0 | 9.422^*^ | .523 | <.001 | 7.998 | 10.847 |
|  |  | Day 6 | -11.226^*^ | .523 | <.001 | -12.651 | -9.802 |
|  |  | Day 9 | -19.911^*^ | .523 | <.001 | -21.335 | -18.486 |
|  | Day 6 | Day 0 | 20.649^*^ | .523 | <.001 | 19.224 | 22.073 |
|  |  | Day 3 | 11.226^*^ | .523 | <.001 | 9.802 | 12.651 |
|  |  | Day 9 | -8.684^*^ | .523 | <.001 | -10.109 | -7.260 |
|  | Day 9 | Day 0 | 29.333^*^ | .523 | <.001 | 27.908 | 30.757 |
|  |  | Day 3 | 19.911^*^ | .523 | <.001 | 18.486 | 21.335 |
|  |  | Day 6 | 8.684^*^ | .523 | <.001 | 7.260 | 10.109 |
| High blue light | Day 0 | Day 3 | -11.918^*^ | .523 | <.001 | -13.342 | -10.493 |
|  |  | Day 6 | -20.606^*^ | .523 | <.001 | -22.030 | -19.181 |
|  |  | Day 9 | -31.397^*^ | .523 | <.001 | -32.822 | -29.973 |
|  | Day 3 | Day 0 | 11.918^*^ | .523 | <.001 | 10.493 | 13.342 |
|  |  | Day 6 | -8.688^*^ | .523 | <.001 | -10.112 | -7.264 |
|  |  | Day 9 | -19.480^*^ | .523 | <.001 | -20.904 | -18.055 |
|  | Day 6 | Day 0 | 20.606^*^ | .523 | <.001 | 19.181 | 22.030 |
|  |  | Day 3 | 8.688^*^ | .523 | <.001 | 7.264 | 10.112 |
|  |  | Day 9 | -10.792^*^ | .523 | <.001 | -12.216 | -9.367 |
|  | Day 9 | Day 0 | 31.397^*^ | .523 | <.001 | 29.973 | 32.822 |
|  |  | Day 3 | 19.480^*^ | .523 | <.001 | 18.055 | 20.904 |
|  |  | Day 6 | 10.792^*^ | .523 | <.001 | 9.367 | 12.216 |
| Based on estimated marginal means | | | | | | | |
| *. The mean difference is significant at the .05 level. | | | | | | | |
| b. Adjustment for multiple comparisons: Bonferroni. | | | | | | | |

| Univariate Tests | | | | | | |
| --- | --- | --- | --- | --- | --- | --- |
| Dependent Variable: Weight loss | | | | | | |
| Light | | Sum of Squares | df | Mean Square | F | Sig. |
| White light | Contrast | 5920.349 | 3 | 1973.450 | 1201.717 | <.001 |
|  | Error | 105.100 | 64 | 1.642 |  |  |
| High blue light | Contrast | 6371.480 | 3 | 2123.827 | 1293.288 | <.001 |
|  | Error | 105.100 | 64 | 1.642 |  |  |
| Each F tests the simple effects of Day within each level combination of the other effects shown. These tests are based on the linearly independent pairwise comparisons among the estimated marginal means. | | | | | | |

**SNP * Day**

| Pairwise Comparisons | | | | | | | |
| --- | --- | --- | --- | --- | --- | --- | --- |
| Dependent Variable: Weight loss | | | | | | | |
| Day | (I) SNP (µM) | (J) SNP (µM) | Mean Difference (I-J) | Std. Error | Sig.^b^ | 95% Confidence Interval for Difference^b^ | |
|  |  |  |  |  |  | Lower Bound | Upper Bound |
| Day 0 | 0 | 100 | 3.553E-15 | .740 | 1.000 | -2.014 | 2.014 |
|  |  | 200 | -1.776E-15 | .740 | 1.000 | -2.014 | 2.014 |
|  |  | 300 | -6.661E-16 | .740 | 1.000 | -2.014 | 2.014 |
|  | 100 | 0 | -3.553E-15 | .740 | 1.000 | -2.014 | 2.014 |
|  |  | 200 | -1.776E-15 | .740 | 1.000 | -2.014 | 2.014 |
|  |  | 300 | -3.553E-15 | .740 | 1.000 | -2.014 | 2.014 |
|  | 200 | 0 | 1.776E-15 | .740 | 1.000 | -2.014 | 2.014 |
|  |  | 100 | 1.776E-15 | .740 | 1.000 | -2.014 | 2.014 |
|  |  | 300 | .000 | .740 | 1.000 | -2.014 | 2.014 |
|  | 300 | 0 | 6.661E-16 | .740 | 1.000 | -2.014 | 2.014 |
|  |  | 100 | 3.553E-15 | .740 | 1.000 | -2.014 | 2.014 |
|  |  | 200 | .000 | .740 | 1.000 | -2.014 | 2.014 |
| Day 3 | 0 | 100 | 1.614 | .740 | .197 | -.400 | 3.629 |
|  |  | 200 | 1.418 | .740 | .358 | -.596 | 3.433 |
|  |  | 300 | -.432 | .740 | 1.000 | -2.447 | 1.582 |
|  | 100 | 0 | -1.614 | .740 | .197 | -3.629 | .400 |
|  |  | 200 | -.196 | .740 | 1.000 | -2.211 | 1.818 |
|  |  | 300 | -2.047^*^ | .740 | .044 | -4.061 | -.032 |
|  | 200 | 0 | -1.418 | .740 | .358 | -3.433 | .596 |
|  |  | 100 | .196 | .740 | 1.000 | -1.818 | 2.211 |
|  |  | 300 | -1.851 | .740 | .090 | -3.865 | .164 |
|  | 300 | 0 | .432 | .740 | 1.000 | -1.582 | 2.447 |
|  |  | 100 | 2.047^*^ | .740 | .044 | .032 | 4.061 |
|  |  | 200 | 1.851 | .740 | .090 | -.164 | 3.865 |
| Day 6 | 0 | 100 | 4.945^*^ | .740 | <.001 | 2.931 | 6.960 |
|  |  | 200 | 1.397 | .740 | .382 | -.618 | 3.411 |
|  |  | 300 | -3.257^*^ | .740 | <.001 | -5.271 | -1.242 |
|  | 100 | 0 | -4.945^*^ | .740 | <.001 | -6.960 | -2.931 |
|  |  | 200 | -3.549^*^ | .740 | <.001 | -5.563 | -1.534 |
|  |  | 300 | -8.202^*^ | .740 | <.001 | -10.216 | -6.188 |
|  | 200 | 0 | -1.397 | .740 | .382 | -3.411 | .618 |
|  |  | 100 | 3.549^*^ | .740 | <.001 | 1.534 | 5.563 |
|  |  | 300 | -4.653^*^ | .740 | <.001 | -6.668 | -2.639 |
|  | 300 | 0 | 3.257^*^ | .740 | <.001 | 1.242 | 5.271 |
|  |  | 100 | 8.202^*^ | .740 | <.001 | 6.188 | 10.216 |
|  |  | 200 | 4.653^*^ | .740 | <.001 | 2.639 | 6.668 |
| Day 9 | 0 | 100 | 11.492^*^ | .740 | <.001 | 9.478 | 13.507 |
|  |  | 200 | 4.779^*^ | .740 | <.001 | 2.765 | 6.793 |
|  |  | 300 | -2.266^*^ | .740 | .019 | -4.280 | -.252 |
|  | 100 | 0 | -11.492^*^ | .740 | <.001 | -13.507 | -9.478 |
|  |  | 200 | -6.713^*^ | .740 | <.001 | -8.728 | -4.699 |
|  |  | 300 | -13.758^*^ | .740 | <.001 | -15.773 | -11.744 |
|  | 200 | 0 | -4.779^*^ | .740 | <.001 | -6.793 | -2.765 |
|  |  | 100 | 6.713^*^ | .740 | <.001 | 4.699 | 8.728 |
|  |  | 300 | -7.045^*^ | .740 | <.001 | -9.059 | -5.031 |
|  | 300 | 0 | 2.266^*^ | .740 | .019 | .252 | 4.280 |
|  |  | 100 | 13.758^*^ | .740 | <.001 | 11.744 | 15.773 |
|  |  | 200 | 7.045^*^ | .740 | <.001 | 5.031 | 9.059 |
| Based on estimated marginal means | | | | | | | |
| *. The mean difference is significant at the .05 level. | | | | | | | |
| b. Adjustment for multiple comparisons: Bonferroni. | | | | | | | |

| Univariate Tests | | | | | | |
| --- | --- | --- | --- | --- | --- | --- |
| Dependent Variable: Weight loss | | | | | | |
| Day | | Sum of Squares | df | Mean Square | F | Sig. |
| Day 0 | Contrast | 9.548E-29 | 3 | 3.183E-29 | .000 | 1.000 |
|  | Error | 105.100 | 64 | 1.642 |  |  |
| Day 3 | Contrast | 18.687 | 3 | 6.229 | 3.793 | .014 |
|  | Error | 105.100 | 64 | 1.642 |  |  |
| Day 6 | Contrast | 207.798 | 3 | 69.266 | 42.179 | <.001 |
|  | Error | 105.100 | 64 | 1.642 |  |  |
| Day 9 | Contrast | 666.060 | 3 | 222.020 | 135.197 | <.001 |
|  | Error | 105.100 | 64 | 1.642 |  |  |
| Each F tests the simple effects of SNP within each level combination of the other effects shown. These tests are based on the linearly independent pairwise comparisons among the estimated marginal means. | | | | | | |

| Pairwise Comparisons | | | | | | | |
| --- | --- | --- | --- | --- | --- | --- | --- |
| Dependent Variable: Weight loss | | | | | | | |
| SNP (µM) | (I) Day | (J) Day | Mean Difference (I-J) | Std. Error | Sig.^b^ | 95% Confidence Interval for Difference^b^ | |
|  |  |  |  |  |  | Lower Bound | Upper Bound |
| 0 | Day 0 | Day 3 | -11.320^*^ | .740 | <.001 | -13.335 | -9.306 |
|  |  | Day 6 | -21.399^*^ | .740 | <.001 | -23.413 | -19.384 |
|  |  | Day 9 | -33.867^*^ | .740 | <.001 | -35.881 | -31.852 |
|  | Day 3 | Day 0 | 11.320^*^ | .740 | <.001 | 9.306 | 13.335 |
|  |  | Day 6 | -10.078^*^ | .740 | <.001 | -12.093 | -8.064 |
|  |  | Day 9 | -22.546^*^ | .740 | <.001 | -24.561 | -20.532 |
|  | Day 6 | Day 0 | 21.399^*^ | .740 | <.001 | 19.384 | 23.413 |
|  |  | Day 3 | 10.078^*^ | .740 | <.001 | 8.064 | 12.093 |
|  |  | Day 9 | -12.468^*^ | .740 | <.001 | -14.482 | -10.454 |
|  | Day 9 | Day 0 | 33.867^*^ | .740 | <.001 | 31.852 | 35.881 |
|  |  | Day 3 | 22.546^*^ | .740 | <.001 | 20.532 | 24.561 |
|  |  | Day 6 | 12.468^*^ | .740 | <.001 | 10.454 | 14.482 |
| 100 | Day 0 | Day 3 | -9.706^*^ | .740 | <.001 | -11.720 | -7.691 |
|  |  | Day 6 | -16.453^*^ | .740 | <.001 | -18.468 | -14.439 |
|  |  | Day 9 | -22.374^*^ | .740 | <.001 | -24.389 | -20.360 |
|  | Day 3 | Day 0 | 9.706^*^ | .740 | <.001 | 7.691 | 11.720 |
|  |  | Day 6 | -6.748^*^ | .740 | <.001 | -8.762 | -4.733 |
|  |  | Day 9 | -12.668^*^ | .740 | <.001 | -14.683 | -10.654 |
|  | Day 6 | Day 0 | 16.453^*^ | .740 | <.001 | 14.439 | 18.468 |
|  |  | Day 3 | 6.748^*^ | .740 | <.001 | 4.733 | 8.762 |
|  |  | Day 9 | -5.921^*^ | .740 | <.001 | -7.935 | -3.906 |
|  | Day 9 | Day 0 | 22.374^*^ | .740 | <.001 | 20.360 | 24.389 |
|  |  | Day 3 | 12.668^*^ | .740 | <.001 | 10.654 | 14.683 |
|  |  | Day 6 | 5.921^*^ | .740 | <.001 | 3.906 | 7.935 |
| 200 | Day 0 | Day 3 | -9.902^*^ | .740 | <.001 | -11.916 | -7.887 |
|  |  | Day 6 | -20.002^*^ | .740 | <.001 | -22.016 | -17.987 |
|  |  | Day 9 | -29.088^*^ | .740 | <.001 | -31.102 | -27.073 |
|  | Day 3 | Day 0 | 9.902^*^ | .740 | <.001 | 7.887 | 11.916 |
|  |  | Day 6 | -10.100^*^ | .740 | <.001 | -12.114 | -8.086 |
|  |  | Day 9 | -19.186^*^ | .740 | <.001 | -21.200 | -17.171 |
|  | Day 6 | Day 0 | 20.002^*^ | .740 | <.001 | 17.987 | 22.016 |
|  |  | Day 3 | 10.100^*^ | .740 | <.001 | 8.086 | 12.114 |
|  |  | Day 9 | -9.086^*^ | .740 | <.001 | -11.100 | -7.071 |
|  | Day 9 | Day 0 | 29.088^*^ | .740 | <.001 | 27.073 | 31.102 |
|  |  | Day 3 | 19.186^*^ | .740 | <.001 | 17.171 | 21.200 |
|  |  | Day 6 | 9.086^*^ | .740 | <.001 | 7.071 | 11.100 |
| 300 | Day 0 | Day 3 | -11.753^*^ | .740 | <.001 | -13.767 | -9.738 |
|  |  | Day 6 | -24.655^*^ | .740 | <.001 | -26.670 | -22.641 |
|  |  | Day 9 | -36.132^*^ | .740 | <.001 | -38.147 | -34.118 |
|  | Day 3 | Day 0 | 11.753^*^ | .740 | <.001 | 9.738 | 13.767 |
|  |  | Day 6 | -12.903^*^ | .740 | <.001 | -14.917 | -10.888 |
|  |  | Day 9 | -24.380^*^ | .740 | <.001 | -26.394 | -22.366 |
|  | Day 6 | Day 0 | 24.655^*^ | .740 | <.001 | 22.641 | 26.670 |
|  |  | Day 3 | 12.903^*^ | .740 | <.001 | 10.888 | 14.917 |
|  |  | Day 9 | -11.477^*^ | .740 | <.001 | -13.492 | -9.463 |
|  | Day 9 | Day 0 | 36.132^*^ | .740 | <.001 | 34.118 | 38.147 |
|  |  | Day 3 | 24.380^*^ | .740 | <.001 | 22.366 | 26.394 |
|  |  | Day 6 | 11.477^*^ | .740 | <.001 | 9.463 | 13.492 |
| Based on estimated marginal means | | | | | | | |
| *. The mean difference is significant at the .05 level. | | | | | | | |
| b. Adjustment for multiple comparisons: Bonferroni. | | | | | | | |

| Univariate Tests | | | | | | |
| --- | --- | --- | --- | --- | --- | --- |
| Dependent Variable: Weight loss | | | | | | |
| SNP (µM) | | Sum of Squares | df | Mean Square | F | Sig. |
| 0 | Contrast | 3747.514 | 3 | 1249.171 | 760.673 | <.001 |
|  | Error | 105.100 | 64 | 1.642 |  |  |
| 100 | Contrast | 1659.890 | 3 | 553.297 | 336.926 | <.001 |
|  | Error | 105.100 | 64 | 1.642 |  |  |
| 200 | Contrast | 2845.277 | 3 | 948.426 | 577.536 | <.001 |
|  | Error | 105.100 | 64 | 1.642 |  |  |
| 300 | Contrast | 4416.236 | 3 | 1472.079 | 896.411 | <.001 |
|  | Error | 105.100 | 64 | 1.642 |  |  |
| Each F tests the simple effects of Day within each level combination of the other effects shown. These tests are based on the linearly independent pairwise comparisons among the estimated marginal means. | | | | | | |

**Light * SNP * Day**

| Pairwise Comparisons | | | | | | | | |
| --- | --- | --- | --- | --- | --- | --- | --- | --- |
| Dependent Variable: Weight loss | | | | | | | | |
| SNP  (µM) | Day | (I) Light | (J) Light | Mean Difference (I-J) | Std. Error | Sig.^b^ | 95% Confidence Interval for Difference^b^ | |
|  |  |  |  |  |  |  | Lower Bound | Upper Bound |
| 0 | Day 0 | White light | High blue light | -4.441E-16 | 1.046 | 1.000 | -2.090 | 2.090 |
|  |  | High blue light | White light | 4.441E-16 | 1.046 | 1.000 | -2.090 | 2.090 |
|  | Day 3 | White light | High blue light | .136 | 1.046 | .897 | -1.954 | 2.227 |
|  |  | High blue light | White light | -.136 | 1.046 | .897 | -2.227 | 1.954 |
|  | Day 6 | White light | High blue light | 4.091^*^ | 1.046 | <.001 | 2.001 | 6.181 |
|  |  | High blue light | White light | -4.091^*^ | 1.046 | <.001 | -6.181 | -2.001 |
|  | Day 9 | White light | High blue light | 1.414 | 1.046 | .181 | -.676 | 3.505 |
|  |  | High blue light | White light | -1.414 | 1.046 | .181 | -3.505 | .676 |
| 100 | Day 0 | White light | High blue light | .000 | 1.046 | 1.000 | -2.090 | 2.090 |
|  |  | High blue light | White light | .000 | 1.046 | 1.000 | -2.090 | 2.090 |
|  | Day 3 | White light | High blue light | -4.418^*^ | 1.046 | <.001 | -6.508 | -2.328 |
|  |  | High blue light | White light | 4.418^*^ | 1.046 | <.001 | 2.328 | 6.508 |
|  | Day 6 | White light | High blue light | -4.973^*^ | 1.046 | <.001 | -7.064 | -2.883 |
|  |  | High blue light | White light | 4.973^*^ | 1.046 | <.001 | 2.883 | 7.064 |
|  | Day 9 | White light | High blue light | -5.818^*^ | 1.046 | <.001 | -7.908 | -3.727 |
|  |  | High blue light | White light | 5.818^*^ | 1.046 | <.001 | 3.727 | 7.908 |
| 200 | Day 0 | White light | High blue light | -4.441E-16 | 1.046 | 1.000 | -2.090 | 2.090 |
|  |  | High blue light | White light | 4.441E-16 | 1.046 | 1.000 | -2.090 | 2.090 |
|  | Day 3 | White light | High blue light | .502 | 1.046 | .633 | -1.589 | 2.592 |
|  |  | High blue light | White light | -.502 | 1.046 | .633 | -2.592 | 1.589 |
|  | Day 6 | White light | High blue light | 1.486 | 1.046 | .160 | -.605 | 3.576 |
|  |  | High blue light | White light | -1.486 | 1.046 | .160 | -3.576 | .605 |
|  | Day 9 | White light | High blue light | -3.007^*^ | 1.046 | .005 | -5.097 | -.917 |
|  |  | High blue light | White light | 3.007^*^ | 1.046 | .005 | .917 | 5.097 |
| 300 | Day 0 | White light | High blue light | -4.441E-16 | 1.046 | 1.000 | -2.090 | 2.090 |
|  |  | High blue light | White light | 4.441E-16 | 1.046 | 1.000 | -2.090 | 2.090 |
|  | Day 3 | White light | High blue light | -6.202^*^ | 1.046 | <.001 | -8.293 | -4.112 |
|  |  | High blue light | White light | 6.202^*^ | 1.046 | <.001 | 4.112 | 8.293 |
|  | Day 6 | White light | High blue light | -.432 | 1.046 | .681 | -2.522 | 1.658 |
|  |  | High blue light | White light | .432 | 1.046 | .681 | -1.658 | 2.522 |
|  | Day 9 | White light | High blue light | -.848 | 1.046 | .421 | -2.938 | 1.243 |
|  |  | High blue light | White light | .848 | 1.046 | .421 | -1.243 | 2.938 |
| Based on estimated marginal means | | | | | | | | |
| *. The mean difference is significant at the .05 level. | | | | | | | | |
| b. Adjustment for multiple comparisons: Bonferroni. | | | | | | | | |

| Univariate Tests | | | | | | | |
| --- | --- | --- | --- | --- | --- | --- | --- |
| Dependent Variable: Weight loss | | | | | | | |
| SNP (µM) | Day | | Sum of Squares | df | Mean Square | F | Sig. |
| 0 | Day 0 | Contrast | 2.958E-31 | 1 | 2.958E-31 | .000 | 1.000 |
|  |  | Error | 105.100 | 64 | 1.642 |  |  |
|  | Day 3 | Contrast | .028 | 1 | .028 | .017 | .897 |
|  |  | Error | 105.100 | 64 | 1.642 |  |  |
|  | Day 6 | Contrast | 25.104 | 1 | 25.104 | 15.287 | <.001 |
|  |  | Error | 105.100 | 64 | 1.642 |  |  |
|  | Day 9 | Contrast | 3.001 | 1 | 3.001 | 1.827 | .181 |
|  |  | Error | 105.100 | 64 | 1.642 |  |  |
| 100 | Day 0 | Contrast | .000 | 1 | .000 | .000 | 1.000 |
|  |  | Error | 105.100 | 64 | 1.642 |  |  |
|  | Day 3 | Contrast | 29.278 | 1 | 29.278 | 17.829 | <.001 |
|  |  | Error | 105.100 | 64 | 1.642 |  |  |
|  | Day 6 | Contrast | 37.101 | 1 | 37.101 | 22.592 | <.001 |
|  |  | Error | 105.100 | 64 | 1.642 |  |  |
|  | Day 9 | Contrast | 50.768 | 1 | 50.768 | 30.915 | <.001 |
|  |  | Error | 105.100 | 64 | 1.642 |  |  |
| 200 | Day 0 | Contrast | 2.958E-31 | 1 | 2.958E-31 | .000 | 1.000 |
|  |  | Error | 105.100 | 64 | 1.642 |  |  |
|  | Day 3 | Contrast | .378 | 1 | .378 | .230 | .633 |
|  |  | Error | 105.100 | 64 | 1.642 |  |  |
|  | Day 6 | Contrast | 3.311 | 1 | 3.311 | 2.016 | .160 |
|  |  | Error | 105.100 | 64 | 1.642 |  |  |
|  | Day 9 | Contrast | 13.563 | 1 | 13.563 | 8.259 | .005 |
|  |  | Error | 105.100 | 64 | 1.642 |  |  |
| 300 | Day 0 | Contrast | 2.958E-31 | 1 | 2.958E-31 | .000 | 1.000 |
|  |  | Error | 105.100 | 64 | 1.642 |  |  |
|  | Day 3 | Contrast | 57.703 | 1 | 57.703 | 35.138 | <.001 |
|  |  | Error | 105.100 | 64 | 1.642 |  |  |
|  | Day 6 | Contrast | .280 | 1 | .280 | .170 | .681 |
|  |  | Error | 105.100 | 64 | 1.642 |  |  |
|  | Day 9 | Contrast | 1.078 | 1 | 1.078 | .656 | .421 |
|  |  | Error | 105.100 | 64 | 1.642 |  |  |
| Each F tests the simple effects of Light within each level combination of the other effects shown. These tests are based on the linearly independent pairwise comparisons among the estimated marginal means. | | | | | | | |

| Pairwise Comparisons | | | | | | | | |
| --- | --- | --- | --- | --- | --- | --- | --- | --- |
| Dependent Variable: Weight loss | | | | | | | | |
| Light | Day | (I) SNP (µM) | (J) SNP(µM) | Mean Difference (I-J) | Std. Error | Sig.^b^ | 95% Confidence Interval for Difference^b^ | |
|  |  |  |  |  |  |  | Lower Bound | Upper Bound |
| White light | Day 0 | 0 | 100 | 3.553E-15 | 1.046 | 1.000 | -2.849 | 2.849 |
|  |  |  | 200 | -1.776E-15 | 1.046 | 1.000 | -2.849 | 2.849 |
|  |  |  | 300 | -8.882E-16 | 1.046 | 1.000 | -2.849 | 2.849 |
|  |  | 100 | 0 | -3.553E-15 | 1.046 | 1.000 | -2.849 | 2.849 |
|  |  |  | 200 | -3.553E-15 | 1.046 | 1.000 | -2.849 | 2.849 |
|  |  |  | 300 | -3.553E-15 | 1.046 | 1.000 | -2.849 | 2.849 |
|  |  | 200 | 0 | 1.776E-15 | 1.046 | 1.000 | -2.849 | 2.849 |
|  |  |  | 100 | 3.553E-15 | 1.046 | 1.000 | -2.849 | 2.849 |
|  |  |  | 300 | .000 | 1.046 | 1.000 | -2.849 | 2.849 |
|  |  | 300 | 0 | 8.882E-16 | 1.046 | 1.000 | -2.849 | 2.849 |
|  |  |  | 100 | 3.553E-15 | 1.046 | 1.000 | -2.849 | 2.849 |
|  |  |  | 200 | .000 | 1.046 | 1.000 | -2.849 | 2.849 |
|  | Day 3 | 0 | 100 | 3.892^*^ | 1.046 | .003 | 1.043 | 6.741 |
|  |  |  | 200 | 1.236 | 1.046 | 1.000 | -1.613 | 4.085 |
|  |  |  | 300 | 2.737 | 1.046 | .067 | -.112 | 5.586 |
|  |  | 100 | 0 | -3.892^*^ | 1.046 | .003 | -6.741 | -1.043 |
|  |  |  | 200 | -2.656 | 1.046 | .081 | -5.505 | .193 |
|  |  |  | 300 | -1.155 | 1.046 | 1.000 | -4.004 | 1.694 |
|  |  | 200 | 0 | -1.236 | 1.046 | 1.000 | -4.085 | 1.613 |
|  |  |  | 100 | 2.656 | 1.046 | .081 | -.193 | 5.505 |
|  |  |  | 300 | 1.501 | 1.046 | .937 | -1.348 | 4.350 |
|  |  | 300 | 0 | -2.737 | 1.046 | .067 | -5.586 | .112 |
|  |  |  | 100 | 1.155 | 1.046 | 1.000 | -1.694 | 4.004 |
|  |  |  | 200 | -1.501 | 1.046 | .937 | -4.350 | 1.348 |
|  | Day 6 | 0 | 100 | 9.477^*^ | 1.046 | <.001 | 6.628 | 12.326 |
|  |  |  | 200 | 2.699 | 1.046 | .073 | -.150 | 5.548 |
|  |  |  | 300 | -.995 | 1.046 | 1.000 | -3.844 | 1.854 |
|  |  | 100 | 0 | -9.477^*^ | 1.046 | <.001 | -12.326 | -6.628 |
|  |  |  | 200 | -6.778^*^ | 1.046 | <.001 | -9.627 | -3.929 |
|  |  |  | 300 | -10.473^*^ | 1.046 | <.001 | -13.322 | -7.624 |
|  |  | 200 | 0 | -2.699 | 1.046 | .073 | -5.548 | .150 |
|  |  |  | 100 | 6.778^*^ | 1.046 | <.001 | 3.929 | 9.627 |
|  |  |  | 300 | -3.695^*^ | 1.046 | .005 | -6.544 | -.846 |
|  |  | 300 | 0 | .995 | 1.046 | 1.000 | -1.854 | 3.844 |
|  |  |  | 100 | 10.473^*^ | 1.046 | <.001 | 7.624 | 13.322 |
|  |  |  | 200 | 3.695^*^ | 1.046 | .005 | .846 | 6.544 |
|  | Day 9 | 0 | 100 | 15.108^*^ | 1.046 | <.001 | 12.259 | 17.957 |
|  |  |  | 200 | 6.990^*^ | 1.046 | <.001 | 4.141 | 9.839 |
|  |  |  | 300 | -1.135 | 1.046 | 1.000 | -3.984 | 1.714 |
|  |  | 100 | 0 | -15.108^*^ | 1.046 | <.001 | -17.957 | -12.259 |
|  |  |  | 200 | -8.119^*^ | 1.046 | <.001 | -10.968 | -5.270 |
|  |  |  | 300 | -16.243^*^ | 1.046 | <.001 | -19.092 | -13.394 |
|  |  | 200 | 0 | -6.990^*^ | 1.046 | <.001 | -9.839 | -4.141 |
|  |  |  | 100 | 8.119^*^ | 1.046 | <.001 | 5.270 | 10.968 |
|  |  |  | 300 | -8.125^*^ | 1.046 | <.001 | -10.974 | -5.276 |
|  |  | 300 | 0 | 1.135 | 1.046 | 1.000 | -1.714 | 3.984 |
|  |  |  | 100 | 16.243^*^ | 1.046 | <.001 | 13.394 | 19.092 |
|  |  |  | 200 | 8.125^*^ | 1.046 | <.001 | 5.276 | 10.974 |
| High blue light | Day 0 | 0 | 100 | 3.553E-15 | 1.046 | 1.000 | -2.849 | 2.849 |
|  |  |  | 200 | -8.882E-16 | 1.046 | 1.000 | -2.849 | 2.849 |
|  |  |  | 300 | -8.882E-16 | 1.046 | 1.000 | -2.849 | 2.849 |
|  |  | 100 | 0 | -3.553E-15 | 1.046 | 1.000 | -2.849 | 2.849 |
|  |  |  | 200 | -3.553E-15 | 1.046 | 1.000 | -2.849 | 2.849 |
|  |  |  | 300 | -3.553E-15 | 1.046 | 1.000 | -2.849 | 2.849 |
|  |  | 200 | 0 | 8.882E-16 | 1.046 | 1.000 | -2.849 | 2.849 |
|  |  |  | 100 | 3.553E-15 | 1.046 | 1.000 | -2.849 | 2.849 |
|  |  |  | 300 | .000 | 1.046 | 1.000 | -2.849 | 2.849 |
|  |  | 300 | 0 | 8.882E-16 | 1.046 | 1.000 | -2.849 | 2.849 |
|  |  |  | 100 | 3.553E-15 | 1.046 | 1.000 | -2.849 | 2.849 |
|  |  |  | 200 | .000 | 1.046 | 1.000 | -2.849 | 2.849 |
|  | Day 3 | 0 | 100 | -.663 | 1.046 | 1.000 | -3.512 | 2.186 |
|  |  |  | 200 | 1.601 | 1.046 | .785 | -1.248 | 4.450 |
|  |  |  | 300 | -3.602^*^ | 1.046 | .006 | -6.451 | -.753 |
|  |  | 100 | 0 | .663 | 1.046 | 1.000 | -2.186 | 3.512 |
|  |  |  | 200 | 2.264 | 1.046 | .205 | -.585 | 5.113 |
|  |  |  | 300 | -2.939^*^ | 1.046 | .040 | -5.788 | -.090 |
|  |  | 200 | 0 | -1.601 | 1.046 | .785 | -4.450 | 1.248 |
|  |  |  | 100 | -2.264 | 1.046 | .205 | -5.113 | .585 |
|  |  |  | 300 | -5.203^*^ | 1.046 | <.001 | -8.052 | -2.354 |
|  |  | 300 | 0 | 3.602^*^ | 1.046 | .006 | .753 | 6.451 |
|  |  |  | 100 | 2.939^*^ | 1.046 | .040 | .090 | 5.788 |
|  |  |  | 200 | 5.203^*^ | 1.046 | <.001 | 2.354 | 8.052 |
|  | Day 6 | 0 | 100 | .413 | 1.046 | 1.000 | -2.436 | 3.262 |
|  |  |  | 200 | .094 | 1.046 | 1.000 | -2.755 | 2.943 |
|  |  |  | 300 | -5.518^*^ | 1.046 | <.001 | -8.367 | -2.669 |
|  |  | 100 | 0 | -.413 | 1.046 | 1.000 | -3.262 | 2.436 |
|  |  |  | 200 | -.319 | 1.046 | 1.000 | -3.168 | 2.530 |
|  |  |  | 300 | -5.931^*^ | 1.046 | <.001 | -8.780 | -3.082 |
|  |  | 200 | 0 | -.094 | 1.046 | 1.000 | -2.943 | 2.755 |
|  |  |  | 100 | .319 | 1.046 | 1.000 | -2.530 | 3.168 |
|  |  |  | 300 | -5.612^*^ | 1.046 | <.001 | -8.461 | -2.763 |
|  |  | 300 | 0 | 5.518^*^ | 1.046 | <.001 | 2.669 | 8.367 |
|  |  |  | 100 | 5.931^*^ | 1.046 | <.001 | 3.082 | 8.780 |
|  |  |  | 200 | 5.612^*^ | 1.046 | <.001 | 2.763 | 8.461 |
|  | Day 9 | 0 | 100 | 7.876^*^ | 1.046 | <.001 | 5.027 | 10.725 |
|  |  |  | 200 | 2.568 | 1.046 | .101 | -.281 | 5.417 |
|  |  |  | 300 | -3.397^*^ | 1.046 | .011 | -6.246 | -.548 |
|  |  | 100 | 0 | -7.876^*^ | 1.046 | <.001 | -10.725 | -5.027 |
|  |  |  | 200 | -5.308^*^ | 1.046 | <.001 | -8.157 | -2.459 |
|  |  |  | 300 | -11.273^*^ | 1.046 | <.001 | -14.122 | -8.424 |
|  |  | 200 | 0 | -2.568 | 1.046 | .101 | -5.417 | .281 |
|  |  |  | 100 | 5.308^*^ | 1.046 | <.001 | 2.459 | 8.157 |
|  |  |  | 300 | -5.965^*^ | 1.046 | <.001 | -8.814 | -3.116 |
|  |  | 300 | 0 | 3.397^*^ | 1.046 | .011 | .548 | 6.246 |
|  |  |  | 100 | 11.273^*^ | 1.046 | <.001 | 8.424 | 14.122 |
|  |  |  | 200 | 5.965^*^ | 1.046 | <.001 | 3.116 | 8.814 |
| Based on estimated marginal means | | | | | | | | |
| *. The mean difference is significant at the .05 level. | | | | | | | | |
| b. Adjustment for multiple comparisons: Bonferroni. | | | | | | | | |

| Univariate Tests | | | | | | | |
| --- | --- | --- | --- | --- | --- | --- | --- |
| Dependent Variable: Wight loss | | | | | | | |
| Light | Day | | Sum of Squares | df | Mean Square | F | Sig. |
| White light | Day 0 | Contrast | 4.911E-29 | 3 | 1.637E-29 | .000 | 1.000 |
|  |  | Error | 105.100 | 64 | 1.642 |  |  |
|  | Day 3 | Contrast | 26.104 | 3 | 8.701 | 5.299 | .003 |
|  |  | Error | 105.100 | 64 | 1.642 |  |  |
|  | Day 6 | Contrast | 200.524 | 3 | 66.841 | 40.703 | <.001 |
|  |  | Error | 105.100 | 64 | 1.642 |  |  |
|  | Day 9 | Contrast | 505.631 | 3 | 168.544 | 102.633 | <.001 |
|  |  | Error | 105.100 | 64 | 1.642 |  |  |
| High blue light | Day 0 | Contrast | 4.023E-29 | 3 | 1.341E-29 | .000 | 1.000 |
|  |  | Error | 105.100 | 64 | 1.642 |  |  |
|  | Day 3 | Contrast | 42.603 | 3 | 14.201 | 8.648 | <.001 |
|  |  | Error | 105.100 | 64 | 1.642 |  |  |
|  | Day 6 | Contrast | 73.059 | 3 | 24.353 | 14.830 | <.001 |
|  |  | Error | 105.100 | 64 | 1.642 |  |  |
|  | Day 9 | Contrast | 203.266 | 3 | 67.755 | 41.259 | <.001 |
|  |  | Error | 105.100 | 64 | 1.642 |  |  |
| Each F tests the simple effects of SNP within each level combination of the other effects shown. These tests are based on the linearly independent pairwise comparisons among the estimated marginal means. | | | | | | | |

| Pairwise Comparisons | | | | | | | | |
| --- | --- | --- | --- | --- | --- | --- | --- | --- |
| Dependent Variable: Weight loss | | | | | | | | |
| Light | SNP (µM) | (I) Day | (J) Day | Mean Difference (I-J) | Std. Error | Sig.^b^ | 95% Confidence Interval for Difference^b^ | |
|  |  |  |  |  |  |  | Lower Bound | Upper Bound |
| White light | 0 | Day 0 | Day 3 | -11.388^*^ | 1.046 | <.001 | -14.237 | -8.539 |
|  |  |  | Day 6 | -23.444^*^ | 1.046 | <.001 | -26.293 | -20.595 |
|  |  |  | Day 9 | -34.574^*^ | 1.046 | <.001 | -37.423 | -31.725 |
|  |  | Day 3 | Day 0 | 11.388^*^ | 1.046 | <.001 | 8.539 | 14.237 |
|  |  |  | Day 6 | -12.056^*^ | 1.046 | <.001 | -14.905 | -9.207 |
|  |  |  | Day 9 | -23.185^*^ | 1.046 | <.001 | -26.034 | -20.336 |
|  |  | Day 6 | Day 0 | 23.444^*^ | 1.046 | <.001 | 20.595 | 26.293 |
|  |  |  | Day 3 | 12.056^*^ | 1.046 | <.001 | 9.207 | 14.905 |
|  |  |  | Day 9 | -11.130^*^ | 1.046 | <.001 | -13.979 | -8.281 |
|  |  | Day 9 | Day 0 | 34.574^*^ | 1.046 | <.001 | 31.725 | 37.423 |
|  |  |  | Day 3 | 23.185^*^ | 1.046 | <.001 | 20.336 | 26.034 |
|  |  |  | Day 6 | 11.130^*^ | 1.046 | <.001 | 8.281 | 13.979 |
|  | 100 | Day 0 | Day 3 | -7.497^*^ | 1.046 | <.001 | -10.346 | -4.648 |
|  |  |  | Day 6 | -13.967^*^ | 1.046 | <.001 | -16.816 | -11.118 |
|  |  |  | Day 9 | -19.465^*^ | 1.046 | <.001 | -22.314 | -16.616 |
|  |  | Day 3 | Day 0 | 7.497^*^ | 1.046 | <.001 | 4.648 | 10.346 |
|  |  |  | Day 6 | -6.470^*^ | 1.046 | <.001 | -9.319 | -3.621 |
|  |  |  | Day 9 | -11.969^*^ | 1.046 | <.001 | -14.818 | -9.120 |
|  |  | Day 6 | Day 0 | 13.967^*^ | 1.046 | <.001 | 11.118 | 16.816 |
|  |  |  | Day 3 | 6.470^*^ | 1.046 | <.001 | 3.621 | 9.319 |
|  |  |  | Day 9 | -5.499^*^ | 1.046 | <.001 | -8.348 | -2.650 |
|  |  | Day 9 | Day 0 | 19.465^*^ | 1.046 | <.001 | 16.616 | 22.314 |
|  |  |  | Day 3 | 11.969^*^ | 1.046 | <.001 | 9.120 | 14.818 |
|  |  |  | Day 6 | 5.499^*^ | 1.046 | <.001 | 2.650 | 8.348 |
|  | 200 | Day 0 | Day 3 | -10.153^*^ | 1.046 | <.001 | -13.002 | -7.304 |
|  |  |  | Day 6 | -20.745^*^ | 1.046 | <.001 | -23.594 | -17.896 |
|  |  |  | Day 9 | -27.584^*^ | 1.046 | <.001 | -30.433 | -24.735 |
|  |  | Day 3 | Day 0 | 10.153^*^ | 1.046 | <.001 | 7.304 | 13.002 |
|  |  |  | Day 6 | -10.592^*^ | 1.046 | <.001 | -13.441 | -7.743 |
|  |  |  | Day 9 | -17.431^*^ | 1.046 | <.001 | -20.280 | -14.582 |
|  |  | Day 6 | Day 0 | 20.745^*^ | 1.046 | <.001 | 17.896 | 23.594 |
|  |  |  | Day 3 | 10.592^*^ | 1.046 | <.001 | 7.743 | 13.441 |
|  |  |  | Day 9 | -6.839^*^ | 1.046 | <.001 | -9.688 | -3.990 |
|  |  | Day 9 | Day 0 | 27.584^*^ | 1.046 | <.001 | 24.735 | 30.433 |
|  |  |  | Day 3 | 17.431^*^ | 1.046 | <.001 | 14.582 | 20.280 |
|  |  |  | Day 6 | 6.839^*^ | 1.046 | <.001 | 3.990 | 9.688 |
|  | 300 | Day 0 | Day 3 | -8.651^*^ | 1.046 | <.001 | -11.500 | -5.802 |
|  |  |  | Day 6 | -24.439^*^ | 1.046 | <.001 | -27.288 | -21.590 |
|  |  |  | Day 9 | -35.709^*^ | 1.046 | <.001 | -38.558 | -32.860 |
|  |  | Day 3 | Day 0 | 8.651^*^ | 1.046 | <.001 | 5.802 | 11.500 |
|  |  |  | Day 6 | -15.788^*^ | 1.046 | <.001 | -18.637 | -12.939 |
|  |  |  | Day 9 | -27.057^*^ | 1.046 | <.001 | -29.906 | -24.208 |
|  |  | Day 6 | Day 0 | 24.439^*^ | 1.046 | <.001 | 21.590 | 27.288 |
|  |  |  | Day 3 | 15.788^*^ | 1.046 | <.001 | 12.939 | 18.637 |
|  |  |  | Day 9 | -11.269^*^ | 1.046 | <.001 | -14.118 | -8.420 |
|  |  | Day 9 | Day 0 | 35.709^*^ | 1.046 | <.001 | 32.860 | 38.558 |
|  |  |  | Day 3 | 27.057^*^ | 1.046 | <.001 | 24.208 | 29.906 |
|  |  |  | Day 6 | 11.269^*^ | 1.046 | <.001 | 8.420 | 14.118 |
| High blue light | 0 | Day 0 | Day 3 | -11.252^*^ | 1.046 | <.001 | -14.101 | -8.403 |
|  |  |  | Day 6 | -19.353^*^ | 1.046 | <.001 | -22.202 | -16.504 |
|  |  |  | Day 9 | -33.159^*^ | 1.046 | <.001 | -36.008 | -30.310 |
|  |  | Day 3 | Day 0 | 11.252^*^ | 1.046 | <.001 | 8.403 | 14.101 |
|  |  |  | Day 6 | -8.101^*^ | 1.046 | <.001 | -10.950 | -5.252 |
|  |  |  | Day 9 | -21.907^*^ | 1.046 | <.001 | -24.756 | -19.058 |
|  |  | Day 6 | Day 0 | 19.353^*^ | 1.046 | <.001 | 16.504 | 22.202 |
|  |  |  | Day 3 | 8.101^*^ | 1.046 | <.001 | 5.252 | 10.950 |
|  |  |  | Day 9 | -13.806^*^ | 1.046 | <.001 | -16.655 | -10.957 |
|  |  | Day 9 | Day 0 | 33.159^*^ | 1.046 | <.001 | 30.310 | 36.008 |
|  |  |  | Day 3 | 21.907^*^ | 1.046 | <.001 | 19.058 | 24.756 |
|  |  |  | Day 6 | 13.806^*^ | 1.046 | <.001 | 10.957 | 16.655 |
|  | 100 | Day 0 | Day 3 | -11.915^*^ | 1.046 | <.001 | -14.764 | -9.066 |
|  |  |  | Day 6 | -18.940^*^ | 1.046 | <.001 | -21.789 | -16.091 |
|  |  |  | Day 9 | -25.283^*^ | 1.046 | <.001 | -28.132 | -22.434 |
|  |  | Day 3 | Day 0 | 11.915^*^ | 1.046 | <.001 | 9.066 | 14.764 |
|  |  |  | Day 6 | -7.025^*^ | 1.046 | <.001 | -9.874 | -4.176 |
|  |  |  | Day 9 | -13.368^*^ | 1.046 | <.001 | -16.217 | -10.519 |
|  |  | Day 6 | Day 0 | 18.940^*^ | 1.046 | <.001 | 16.091 | 21.789 |
|  |  |  | Day 3 | 7.025^*^ | 1.046 | <.001 | 4.176 | 9.874 |
|  |  |  | Day 9 | -6.343^*^ | 1.046 | <.001 | -9.192 | -3.494 |
|  |  | Day 9 | Day 0 | 25.283^*^ | 1.046 | <.001 | 22.434 | 28.132 |
|  |  |  | Day 3 | 13.368^*^ | 1.046 | <.001 | 10.519 | 16.217 |
|  |  |  | Day 6 | 6.343^*^ | 1.046 | <.001 | 3.494 | 9.192 |
|  | 200 | Day 0 | Day 3 | -9.651^*^ | 1.046 | <.001 | -12.500 | -6.802 |
|  |  |  | Day 6 | -19.259^*^ | 1.046 | <.001 | -22.108 | -16.410 |
|  |  |  | Day 9 | -30.591^*^ | 1.046 | <.001 | -33.440 | -27.742 |
|  |  | Day 3 | Day 0 | 9.651^*^ | 1.046 | <.001 | 6.802 | 12.500 |
|  |  |  | Day 6 | -9.608^*^ | 1.046 | <.001 | -12.457 | -6.759 |
|  |  |  | Day 9 | -20.940^*^ | 1.046 | <.001 | -23.789 | -18.091 |
|  |  | Day 6 | Day 0 | 19.259^*^ | 1.046 | <.001 | 16.410 | 22.108 |
|  |  |  | Day 3 | 9.608^*^ | 1.046 | <.001 | 6.759 | 12.457 |
|  |  |  | Day 9 | -11.332^*^ | 1.046 | <.001 | -14.181 | -8.483 |
|  |  | Day 9 | Day 0 | 30.591^*^ | 1.046 | <.001 | 27.742 | 33.440 |
|  |  |  | Day 3 | 20.940^*^ | 1.046 | <.001 | 18.091 | 23.789 |
|  |  |  | Day 6 | 11.332^*^ | 1.046 | <.001 | 8.483 | 14.181 |
|  | 300 | Day 0 | Day 3 | -14.854^*^ | 1.046 | <.001 | -17.703 | -12.005 |
|  |  |  | Day 6 | -24.871^*^ | 1.046 | <.001 | -27.720 | -22.022 |
|  |  |  | Day 9 | -36.556^*^ | 1.046 | <.001 | -39.405 | -33.707 |
|  |  | Day 3 | Day 0 | 14.854^*^ | 1.046 | <.001 | 12.005 | 17.703 |
|  |  |  | Day 6 | -10.018^*^ | 1.046 | <.001 | -12.867 | -7.169 |
|  |  |  | Day 9 | -21.703^*^ | 1.046 | <.001 | -24.552 | -18.854 |
|  |  | Day 6 | Day 0 | 24.871^*^ | 1.046 | <.001 | 22.022 | 27.720 |
|  |  |  | Day 3 | 10.018^*^ | 1.046 | <.001 | 7.169 | 12.867 |
|  |  |  | Day 9 | -11.685^*^ | 1.046 | <.001 | -14.534 | -8.836 |
|  |  | Day 9 | Day 0 | 36.556^*^ | 1.046 | <.001 | 33.707 | 39.405 |
|  |  |  | Day 3 | 21.703^*^ | 1.046 | <.001 | 18.854 | 24.552 |
|  |  |  | Day 6 | 11.685^*^ | 1.046 | <.001 | 8.836 | 14.534 |
| Based on estimated marginal means | | | | | | | | |
| *. The mean difference is significant at the .05 level. | | | | | | | | |
| b. Adjustment for multiple comparisons: Bonferroni. | | | | | | | | |

| Univariate Tests | | | | | | | |
| --- | --- | --- | --- | --- | --- | --- | --- |
| Dependent Variable: Weight loss | | | | | | | |
| Light | SNP (µM) | | Sum of Squares | df | Mean Square | F | Sig. |
| White light | 0 | Contrast | 2011.066 | 3 | 670.355 | 408.208 | <.001 |
|  |  | Error | 105.100 | 64 | 1.642 |  |  |
|  | 100 | Contrast | 634.134 | 3 | 211.378 | 128.717 | <.001 |
|  |  | Error | 105.100 | 64 | 1.642 |  |  |
|  | 200 | Contrast | 1317.835 | 3 | 439.278 | 267.495 | <.001 |
|  |  | Error | 105.100 | 64 | 1.642 |  |  |
|  | 300 | Contrast | 2291.695 | 3 | 763.898 | 465.170 | <.001 |
|  |  | Error | 105.100 | 64 | 1.642 |  |  |
| High blue light | 0 | Contrast | 1752.645 | 3 | 584.215 | 355.753 | <.001 |
|  |  | Error | 105.100 | 64 | 1.642 |  |  |
|  | 100 | Contrast | 1056.161 | 3 | 352.054 | 214.380 | <.001 |
|  |  | Error | 105.100 | 64 | 1.642 |  |  |
|  | 200 | Contrast | 1544.304 | 3 | 514.768 | 313.464 | <.001 |
|  |  | Error | 105.100 | 64 | 1.642 |  |  |
|  | 300 | Contrast | 2162.609 | 3 | 720.870 | 438.968 | <.001 |
|  |  | Error | 105.100 | 64 | 1.642 |  |  |
| Each F tests the simple effects of Day within each level combination of the other effects shown. These tests are based on the linearly independent pairwise comparisons among the estimated marginal means. | | | | | | | |

**Post Hoc Tests**

| Weight loss | | | | | |
| --- | --- | --- | --- | --- | --- |
| Duncan^a,b^ | | | | | |
| SNP (µM) | N | Subset | | | |
|  |  | 1 | 2 | 3 | 4 |
| 100 | 24 | 12.1333 |  |  |  |
| 200 | 24 |  | 14.7478 |  |  |
| 0 | 24 |  |  | 16.6463 |  |
| 300 | 24 |  |  |  | 18.1351 |
| Sig. |  | 1.000 | 1.000 | 1.000 | 1.000 |
| Means for groups in homogeneous subsets are displayed.  Based on observed means.  The error term is Mean Square (Error) = 1.642. | | | | | |
| a. Uses Harmonic Mean Sample Size = 24.000. | | | | | |
| b. Alpha = .05. | | | | | |

| Weight loss | | | | | |
| --- | --- | --- | --- | --- | --- |
| Duncan^a,b^ | | | | | |
| Day | N | Subset | | | |
|  |  | 1 | 2 | 3 | 4 |
| Day 0 | 24 | .0000 |  |  |  |
| Day 3 | 24 |  | 10.6700 |  |  |
| Day 6 | 24 |  |  | 20.6273 |  |
| Day 9 | 24 |  |  |  | 30.3652 |
| Sig. |  | 1.000 | 1.000 | 1.000 | 1.000 |
| Means for groups in homogeneous subsets are displayed.  Based on observed means.  The error term is Mean Square (Error) = 1.642. | | | | | |
| a. Uses Harmonic Mean Sample Size = 24.000. | | | | | |
| b. Alpha = .05. | | | | | |

**Senescence symptom score (SSC)**

| Tests of Between-Subjects Effects | | | | | | |
| --- | --- | --- | --- | --- | --- | --- |
| Dependent Variable: SSC | | | | | | |
| Source | Type III Sum of Squares | df | Mean Square | F | Sig. | Partial Eta Squared |
| Corrected Model | 97.990a | 31 | 3.161 | 12.644 | <.001 | 0.86 |
| Intercept | 455.01 | 1 | 455.01 | 1820.042 | <.001 | 0.966 |
| Light | 0.844 | 1 | 0.844 | 3.375 | 0.071 | 0.05 |
| SNP | 0.948 | 3 | 0.316 | 1.264 | 0.294 | 0.056 |
| Day | 92.448 | 3 | 30.816 | 123.264 | <.001 | 0.852 |
| Light * SNP | 0.615 | 3 | 0.205 | 0.819 | 0.488 | 0.037 |
| Light * Day | 0.281 | 3 | 0.094 | 0.375 | 0.771 | 0.017 |
| SNP * Day | 2.427 | 9 | 0.27 | 1.079 | 0.391 | 0.132 |
| Light * SNP * Day | 0.427 | 9 | 0.047 | 0.19 | 0.995 | 0.026 |
| Error | 16 | 64 | 0.25 |  |  |  |
| Total | 569 | 96 |  |  |  |  |
| Corrected Total | 113.99 | 95 |  |  |  |  |
| a R Squared = .860 (Adjusted R Squared = .792) | | | | | | |

**Light * SNP**

| Pairwise Comparisons | | | | | | | |
| --- | --- | --- | --- | --- | --- | --- | --- |
| Dependent Variable: SSC | | | | | | | |
| SNP  (µM) | (I) Light | (J) Light | Mean Difference (I-J) | Std. Error | Sig.b | 95% Confidence Interval for Differenceb | |
|  |  |  |  |  |  | Lower Bound | Upper Bound |
| 0 | White light | High blue light | .417* | 0.204 | 0.045 | 0.009 | 0.824 |
|  | High blue light | White light | -.417* | 0.204 | 0.045 | -0.824 | -0.009 |
| 100 | White light | High blue light | 0.083 | 0.204 | 0.684 | -0.324 | 0.491 |
|  | High blue light | White light | -0.083 | 0.204 | 0.684 | -0.491 | 0.324 |
| 200 | White light | High blue light | 0.25 | 0.204 | 0.225 | -0.158 | 0.658 |
|  | High blue light | White light | -0.25 | 0.204 | 0.225 | -0.658 | 0.158 |
| 300 | White light | High blue light | 1.21E-17 | 0.204 | 1 | -0.408 | 0.408 |
|  | High blue light | White light | -1.21E-17 | 0.204 | 1 | -0.408 | 0.408 |
| Based on estimated marginal means | | | |  |  |  |  |
| * The mean difference is significant at the .05 level. | | | | |  |  |  |
| b Adjustment for multiple comparisons: Bonferroni. | | | | | |  |  |

| Univariate Tests | | | | | | | |
| --- | --- | --- | --- | --- | --- | --- | --- |
| Dependent Variable: SSC | | | | | | | |
| SNP  (µM) |  | Sum of Squares | df | Mean Square | F | Sig. | Partial Eta Squared |
| 0 | Contrast | 1.042 | 1 | 1.042 | 4.167 | 0.045 | 0.061 |
|  | Error | 16 | 64 | 0.25 |  |  |  |
| 100 | Contrast | 0.042 | 1 | 0.042 | 0.167 | 0.684 | 0.003 |
|  | Error | 16 | 64 | 0.25 |  |  |  |
| 200 | Contrast | 0.375 | 1 | 0.375 | 1.5 | 0.225 | 0.023 |
|  | Error | 16 | 64 | 0.25 |  |  |  |
| 300 | Contrast | 8.85E-34 | 1 | 8.85E-34 | 0 | 1 | 0 |
|  | Error | 16 | 64 | 0.25 |  |  |  |
| Each F tests the simple effects of Light within each level combination of the other effects shown. These tests are based on the linearly independent pairwise comparisons among the estimated marginal means. | | | | | | | |

| Pairwise Comparisons | | | | | | | |
| --- | --- | --- | --- | --- | --- | --- | --- |
| Dependent Variable: SSC | | | | | | | |
| Light | (I) SNP  (µM) | (J) SNP  (µM) | Mean Difference (I-J) | Std. Error | Sig.a | 95% Confidence Interval for Differencea | |
|  |  |  |  |  |  | Lower Bound | Upper Bound |
| White light | 0 | 100 | -1.25E-16 | 0.204 | 1 | -0.556 | 0.556 |
|  |  | 200 | 0.167 | 0.204 | 1 | -0.389 | 0.722 |
|  |  | 300 | 0.083 | 0.204 | 1 | -0.472 | 0.639 |
|  | 100 | 0 | 1.25E-16 | 0.204 | 1 | -0.556 | 0.556 |
|  |  | 200 | 0.167 | 0.204 | 1 | -0.389 | 0.722 |
|  |  | 300 | 0.083 | 0.204 | 1 | -0.472 | 0.639 |
|  | 200 | 0 | -0.167 | 0.204 | 1 | -0.722 | 0.389 |
|  |  | 100 | -0.167 | 0.204 | 1 | -0.722 | 0.389 |
|  |  | 300 | -0.083 | 0.204 | 1 | -0.639 | 0.472 |
|  | 300 | 0 | -0.083 | 0.204 | 1 | -0.639 | 0.472 |
|  |  | 100 | -0.083 | 0.204 | 1 | -0.639 | 0.472 |
|  |  | 200 | 0.083 | 0.204 | 1 | -0.472 | 0.639 |
| High blue light | 0 | 100 | -0.333 | 0.204 | 0.644 | -0.889 | 0.222 |
|  |  | 200 | -1.67E-16 | 0.204 | 1 | -0.556 | 0.556 |
|  |  | 300 | -0.333 | 0.204 | 0.644 | -0.889 | 0.222 |
|  | 100 | 0 | 0.333 | 0.204 | 0.644 | -0.222 | 0.889 |
|  |  | 200 | 0.333 | 0.204 | 0.644 | -0.222 | 0.889 |
|  |  | 300 | 2.78E-17 | 0.204 | 1 | -0.556 | 0.556 |
|  | 200 | 0 | 1.67E-16 | 0.204 | 1 | -0.556 | 0.556 |
|  |  | 100 | -0.333 | 0.204 | 0.644 | -0.889 | 0.222 |
|  |  | 300 | -0.333 | 0.204 | 0.644 | -0.889 | 0.222 |
|  | 300 | 0 | 0.333 | 0.204 | 0.644 | -0.222 | 0.889 |
|  |  | 100 | -2.78E-17 | 0.204 | 1 | -0.556 | 0.556 |
|  |  | 200 | 0.333 | 0.204 | 0.644 | -0.222 | 0.889 |
| Based on estimated marginal means | | | |  |  |  |  |
| a Adjustment for multiple comparisons: Bonferroni. | | | | | |  |  |

| Univariate Tests | | | | | | | |
| --- | --- | --- | --- | --- | --- | --- | --- |
| Dependent Variable: SSC | | | | | | | |
| Light |  | Sum of Squares | df | Mean Square | F | Sig. | Partial Eta Squared |
| White light | Contrast | 0.229 | 3 | 0.076 | 0.306 | 0.821 | 0.014 |
|  | Error | 16 | 64 | 0.25 |  |  |  |
| High blue light | Contrast | 1.333 | 3 | 0.444 | 1.778 | 0.16 | 0.077 |
|  | Error | 16 | 64 | 0.25 |  |  |  |
| Each F tests the simple effects of SNP within each level combination of the other effects shown. These tests are based on the linearly independent pairwise comparisons among the estimated marginal means. | | | | | | | |

| Pairwise Comparisons | | | | | | | |
| --- | --- | --- | --- | --- | --- | --- | --- |
| Dependent Variable: SSC | | | | | | | |
| Day | (I) Light | (J) Light | Mean Difference (I-J) | Std. Error | Sig.a | 95% Confidence Interval for Differencea | |
|  |  |  |  |  |  | Lower Bound | Upper Bound |
| Day 0 | White light | High blue light | 1.39E-17 | 0.204 | 1 | -0.408 | 0.408 |
|  | High blue light | White light | -1.39E-17 | 0.204 | 1 | -0.408 | 0.408 |
| Day 3 | White light | High blue light | 0.25 | 0.204 | 0.225 | -0.158 | 0.658 |
|  | High blue light | White light | -0.25 | 0.204 | 0.225 | -0.658 | 0.158 |
| Day 6 | White light | High blue light | 0.25 | 0.204 | 0.225 | -0.158 | 0.658 |
|  | High blue light | White light | -0.25 | 0.204 | 0.225 | -0.658 | 0.158 |
| Day 9 | White light | High blue light | 0.25 | 0.204 | 0.225 | -0.158 | 0.658 |
|  | High blue light | White light | -0.25 | 0.204 | 0.225 | -0.658 | 0.158 |
| Based on estimated marginal means | | | |  |  |  |  |
| a Adjustment for multiple comparisons: Bonferroni. | | | | | |  |  |

**Light * Day**

| Univariate Tests | | | | | | | |
| --- | --- | --- | --- | --- | --- | --- | --- |
| Dependent Variable: SSC | | | | | | | |
| Day |  | Sum of Squares | df | Mean Square | F | Sig. | Partial Eta Squared |
| Day 0 | Contrast | 1.16E-33 | 1 | 1.16E-33 | 0 | 1 | 0 |
|  | Error | 16 | 64 | 0.25 |  |  |  |
| Day 3 | Contrast | 0.375 | 1 | 0.375 | 1.5 | 0.225 | 0.023 |
|  | Error | 16 | 64 | 0.25 |  |  |  |
| Day 6 | Contrast | 0.375 | 1 | 0.375 | 1.5 | 0.225 | 0.023 |
|  | Error | 16 | 64 | 0.25 |  |  |  |
| Day 9 | Contrast | 0.375 | 1 | 0.375 | 1.5 | 0.225 | 0.023 |
|  | Error | 16 | 64 | 0.25 |  |  |  |
| Each F tests the simple effects of Light within each level combination of the other effects shown. These tests are based on the linearly independent pairwise comparisons among the estimated marginal means. | | | | | | | |

| Pairwise Comparisons | | | | | | | |
| --- | --- | --- | --- | --- | --- | --- | --- |
| Dependent Variable: SSC | | | | | | | |
| Light | (I) Day | (J) Day | Mean Difference (I-J) | Std. Error | Sig.b | 95% Confidence Interval for Differenceb | |
|  |  |  |  |  |  | Lower Bound | Upper Bound |
| White light | Day 0 | Day 3 | -.667* | 0.204 | 0.011 | -1.222 | -0.111 |
|  |  | Day 6 | -1.750* | 0.204 | <.001 | -2.306 | -1.194 |
|  |  | Day 9 | -2.667* | 0.204 | <.001 | -3.222 | -2.111 |
|  | Day 3 | Day 0 | .667* | 0.204 | 0.011 | 0.111 | 1.222 |
|  |  | Day 6 | -1.083* | 0.204 | <.001 | -1.639 | -0.528 |
|  |  | Day 9 | -2.000* | 0.204 | <.001 | -2.556 | -1.444 |
|  | Day 6 | Day 0 | 1.750* | 0.204 | <.001 | 1.194 | 2.306 |
|  |  | Day 3 | 1.083* | 0.204 | <.001 | 0.528 | 1.639 |
|  |  | Day 9 | -.917* | 0.204 | <.001 | -1.472 | -0.361 |
|  | Day 9 | Day 0 | 2.667* | 0.204 | <.001 | 2.111 | 3.222 |
|  |  | Day 3 | 2.000* | 0.204 | <.001 | 1.444 | 2.556 |
|  |  | Day 6 | .917* | 0.204 | <.001 | 0.361 | 1.472 |
| High blue light | Day 0 | Day 3 | -0.417 | 0.204 | 0.272 | -0.972 | 0.139 |
|  |  | Day 6 | -1.500* | 0.204 | <.001 | -2.056 | -0.944 |
|  |  | Day 9 | -2.417* | 0.204 | <.001 | -2.972 | -1.861 |
|  | Day 3 | Day 0 | 0.417 | 0.204 | 0.272 | -0.139 | 0.972 |
|  |  | Day 6 | -1.083* | 0.204 | <.001 | -1.639 | -0.528 |
|  |  | Day 9 | -2.000* | 0.204 | <.001 | -2.556 | -1.444 |
|  | Day 6 | Day 0 | 1.500* | 0.204 | <.001 | 0.944 | 2.056 |
|  |  | Day 3 | 1.083* | 0.204 | <.001 | 0.528 | 1.639 |
|  |  | Day 9 | -.917* | 0.204 | <.001 | -1.472 | -0.361 |
|  | Day 9 | Day 0 | 2.417* | 0.204 | <.001 | 1.861 | 2.972 |
|  |  | Day 3 | 2.000* | 0.204 | <.001 | 1.444 | 2.556 |
|  |  | Day 6 | .917* | 0.204 | <.001 | 0.361 | 1.472 |
| Based on estimated marginal means | | | |  |  |  |  |
| * The mean difference is significant at the .05 level. | | | | |  |  |  |
| b Adjustment for multiple comparisons: Bonferroni. | | | | | |  |  |

| Univariate Tests | | | | | | | |
| --- | --- | --- | --- | --- | --- | --- | --- |
| Dependent Variable: SSC | | | | | | | |
| Light |  | Sum of Squares | df | Mean Square | F | Sig. | Partial Eta Squared |
| White light | Contrast | 49.896 | 3 | 16.632 | 66.528 | <.001 | 0.757 |
|  | Error | 16 | 64 | 0.25 |  |  |  |
| High blue light | Contrast | 42.833 | 3 | 14.278 | 57.111 | <.001 | 0.728 |
|  | Error | 16 | 64 | 0.25 |  |  |  |
| Each F tests the simple effects of Day within each level combination of the other effects shown. These tests are based on the linearly independent pairwise comparisons among the estimated marginal means. | | | | | | | |

**SNP * Day**

| Pairwise Comparisons | | | | | | | |
| --- | --- | --- | --- | --- | --- | --- | --- |
| Dependent Variable: SSC | | | | | | | |
| Day | (I) SNP (µM) | (J) SNP  (µM) | Mean Difference (I-J) | Std. Error | Sig.a | 95% Confidence Interval for Differencea | |
|  |  |  |  |  |  | Lower Bound | Upper Bound |
| Day 0 | 0 | 100 | -1.94E-16 | 0.289 | 1 | -0.786 | 0.786 |
|  |  | 200 | -2.78E-16 | 0.289 | 1 | -0.786 | 0.786 |
|  |  | 300 | -1.39E-16 | 0.289 | 1 | -0.786 | 0.786 |
|  | 100 | 0 | 1.94E-16 | 0.289 | 1 | -0.786 | 0.786 |
|  |  | 200 | 5.55E-17 | 0.289 | 1 | -0.786 | 0.786 |
|  |  | 300 | 5.55E-17 | 0.289 | 1 | -0.786 | 0.786 |
|  | 200 | 0 | 2.78E-16 | 0.289 | 1 | -0.786 | 0.786 |
|  |  | 100 | -5.55E-17 | 0.289 | 1 | -0.786 | 0.786 |
|  |  | 300 | 5.55E-17 | 0.289 | 1 | -0.786 | 0.786 |
|  | 300 | 0 | 1.39E-16 | 0.289 | 1 | -0.786 | 0.786 |
|  |  | 100 | -5.55E-17 | 0.289 | 1 | -0.786 | 0.786 |
|  |  | 200 | -5.55E-17 | 0.289 | 1 | -0.786 | 0.786 |
| Day 3 | 0 | 100 | -0.333 | 0.289 | 1 | -1.119 | 0.453 |
|  |  | 200 | -0.167 | 0.289 | 1 | -0.953 | 0.619 |
|  |  | 300 | -0.333 | 0.289 | 1 | -1.119 | 0.453 |
|  | 100 | 0 | 0.333 | 0.289 | 1 | -0.453 | 1.119 |
|  |  | 200 | 0.167 | 0.289 | 1 | -0.619 | 0.953 |
|  |  | 300 | -1.39E-16 | 0.289 | 1 | -0.786 | 0.786 |
|  | 200 | 0 | 0.167 | 0.289 | 1 | -0.619 | 0.953 |
|  |  | 100 | -0.167 | 0.289 | 1 | -0.953 | 0.619 |
|  |  | 300 | -0.167 | 0.289 | 1 | -0.953 | 0.619 |
|  | 300 | 0 | 0.333 | 0.289 | 1 | -0.453 | 1.119 |
|  |  | 100 | 1.39E-16 | 0.289 | 1 | -0.786 | 0.786 |
|  |  | 200 | 0.167 | 0.289 | 1 | -0.619 | 0.953 |
| Day 6 | 0 | 100 | -0.667 | 0.289 | 0.145 | -1.453 | 0.119 |
|  |  | 200 | -0.167 | 0.289 | 1 | -0.953 | 0.619 |
|  |  | 300 | -0.333 | 0.289 | 1 | -1.119 | 0.453 |
|  | 100 | 0 | 0.667 | 0.289 | 0.145 | -0.119 | 1.453 |
|  |  | 200 | 0.5 | 0.289 | 0.528 | -0.286 | 1.286 |
|  |  | 300 | 0.333 | 0.289 | 1 | -0.453 | 1.119 |
|  | 200 | 0 | 0.167 | 0.289 | 1 | -0.619 | 0.953 |
|  |  | 100 | -0.5 | 0.289 | 0.528 | -1.286 | 0.286 |
|  |  | 300 | -0.167 | 0.289 | 1 | -0.953 | 0.619 |
|  | 300 | 0 | 0.333 | 0.289 | 1 | -0.453 | 1.119 |
|  |  | 100 | -0.333 | 0.289 | 1 | -1.119 | 0.453 |
|  |  | 200 | 0.167 | 0.289 | 1 | -0.619 | 0.953 |
| Day 9 | 0 | 100 | 0.333 | 0.289 | 1 | -0.453 | 1.119 |
|  |  | 200 | 0.667 | 0.289 | 0.145 | -0.119 | 1.453 |
|  |  | 300 | 0.167 | 0.289 | 1 | -0.619 | 0.953 |
|  | 100 | 0 | -0.333 | 0.289 | 1 | -1.119 | 0.453 |
|  |  | 200 | 0.333 | 0.289 | 1 | -0.453 | 1.119 |
|  |  | 300 | -0.167 | 0.289 | 1 | -0.953 | 0.619 |
|  | 200 | 0 | -0.667 | 0.289 | 0.145 | -1.453 | 0.119 |
|  |  | 100 | -0.333 | 0.289 | 1 | -1.119 | 0.453 |
|  |  | 300 | -0.5 | 0.289 | 0.528 | -1.286 | 0.286 |
|  | 300 | 0 | -0.167 | 0.289 | 1 | -0.953 | 0.619 |
|  |  | 100 | 0.167 | 0.289 | 1 | -0.619 | 0.953 |
|  |  | 200 | 0.5 | 0.289 | 0.528 | -0.286 | 1.286 |
| Based on estimated marginal means | | | |  |  |  |  |
| a Adjustment for multiple comparisons: Bonferroni. | | | | | |  |  |

| Univariate Tests | | | | | | | |
| --- | --- | --- | --- | --- | --- | --- | --- |
| Dependent Variable: SSC | | | | | | | |
| Day |  | Sum of Squares | df | Mean Square | F | Sig. | Partial Eta Squared |
| Day 0 | Contrast | 2.45E-31 | 3 | 8.17E-32 | 0 | 1 | 0 |
|  | Error | 16 | 64 | 0.25 |  |  |  |
| Day 3 | Contrast | 0.458 | 3 | 0.153 | 0.611 | 0.61 | 0.028 |
|  | Error | 16 | 64 | 0.25 |  |  |  |
| Day 6 | Contrast | 1.458 | 3 | 0.486 | 1.944 | 0.131 | 0.084 |
|  | Error | 16 | 64 | 0.25 |  |  |  |
| Day 9 | Contrast | 1.458 | 3 | 0.486 | 1.944 | 0.131 | 0.084 |
|  | Error | 16 | 64 | 0.25 |  |  |  |
| Each F tests the simple effects of SNP within each level combination of the other effects shown. These tests are based on the linearly independent pairwise comparisons among the estimated marginal means. | | | | | | | |

| Pairwise Comparisons | | | | | | | |
| --- | --- | --- | --- | --- | --- | --- | --- |
| Dependent Variable: SSC | | | | | | | |
| SNP | (I) Day | (J) Day | Mean Difference (I-J) | Std. Error | Sig.b | 95% Confidence Interval for Differenceb | |
|  |  |  |  |  |  | Lower Bound | Upper Bound |
| 0 | Day 0 | Day 3 | -0.333 | 0.289 | 1 | -1.119 | 0.453 |
|  |  | Day 6 | -1.333* | 0.289 | <.001 | -2.119 | -0.547 |
|  |  | Day 9 | -2.833* | 0.289 | <.001 | -3.619 | -2.047 |
|  | Day 3 | Day 0 | 0.333 | 0.289 | 1 | -0.453 | 1.119 |
|  |  | Day 6 | -1.000* | 0.289 | 0.006 | -1.786 | -0.214 |
|  |  | Day 9 | -2.500* | 0.289 | <.001 | -3.286 | -1.714 |
|  | Day 6 | Day 0 | 1.333* | 0.289 | <.001 | 0.547 | 2.119 |
|  |  | Day 3 | 1.000* | 0.289 | 0.006 | 0.214 | 1.786 |
|  |  | Day 9 | -1.500* | 0.289 | <.001 | -2.286 | -0.714 |
|  | Day 9 | Day 0 | 2.833* | 0.289 | <.001 | 2.047 | 3.619 |
|  |  | Day 3 | 2.500* | 0.289 | <.001 | 1.714 | 3.286 |
|  |  | Day 6 | 1.500* | 0.289 | <.001 | 0.714 | 2.286 |
| 100 | Day 0 | Day 3 | -0.667 | 0.289 | 0.145 | -1.453 | 0.119 |
|  |  | Day 6 | -2.000* | 0.289 | <.001 | -2.786 | -1.214 |
|  |  | Day 9 | -2.500* | 0.289 | <.001 | -3.286 | -1.714 |
|  | Day 3 | Day 0 | 0.667 | 0.289 | 0.145 | -0.119 | 1.453 |
|  |  | Day 6 | -1.333* | 0.289 | <.001 | -2.119 | -0.547 |
|  |  | Day 9 | -1.833* | 0.289 | <.001 | -2.619 | -1.047 |
|  | Day 6 | Day 0 | 2.000* | 0.289 | <.001 | 1.214 | 2.786 |
|  |  | Day 3 | 1.333* | 0.289 | <.001 | 0.547 | 2.119 |
|  |  | Day 9 | -0.5 | 0.289 | 0.528 | -1.286 | 0.286 |
|  | Day 9 | Day 0 | 2.500* | 0.289 | <.001 | 1.714 | 3.286 |
|  |  | Day 3 | 1.833* | 0.289 | <.001 | 1.047 | 2.619 |
|  |  | Day 6 | 0.5 | 0.289 | 0.528 | -0.286 | 1.286 |
| 200 | Day 0 | Day 3 | -0.5 | 0.289 | 0.528 | -1.286 | 0.286 |
|  |  | Day 6 | -1.500* | 0.289 | <.001 | -2.286 | -0.714 |
|  |  | Day 9 | -2.167* | 0.289 | <.001 | -2.953 | -1.381 |
|  | Day 3 | Day 0 | 0.5 | 0.289 | 0.528 | -0.286 | 1.286 |
|  |  | Day 6 | -1.000* | 0.289 | 0.006 | -1.786 | -0.214 |
|  |  | Day 9 | -1.667* | 0.289 | <.001 | -2.453 | -0.881 |
|  | Day 6 | Day 0 | 1.500* | 0.289 | <.001 | 0.714 | 2.286 |
|  |  | Day 3 | 1.000* | 0.289 | 0.006 | 0.214 | 1.786 |
|  |  | Day 9 | -0.667 | 0.289 | 0.145 | -1.453 | 0.119 |
|  | Day 9 | Day 0 | 2.167* | 0.289 | <.001 | 1.381 | 2.953 |
|  |  | Day 3 | 1.667* | 0.289 | <.001 | 0.881 | 2.453 |
|  |  | Day 6 | 0.667 | 0.289 | 0.145 | -0.119 | 1.453 |
| 300 | Day 0 | Day 3 | -0.667 | 0.289 | 0.145 | -1.453 | 0.119 |
|  |  | Day 6 | -1.667* | 0.289 | <.001 | -2.453 | -0.881 |
|  |  | Day 9 | -2.667* | 0.289 | <.001 | -3.453 | -1.881 |
|  | Day 3 | Day 0 | 0.667 | 0.289 | 0.145 | -0.119 | 1.453 |
|  |  | Day 6 | -1.000* | 0.289 | 0.006 | -1.786 | -0.214 |
|  |  | Day 9 | -2.000* | 0.289 | <.001 | -2.786 | -1.214 |
|  | Day 6 | Day 0 | 1.667* | 0.289 | <.001 | 0.881 | 2.453 |
|  |  | Day 3 | 1.000* | 0.289 | 0.006 | 0.214 | 1.786 |
|  |  | Day 9 | -1.000* | 0.289 | 0.006 | -1.786 | -0.214 |
|  | Day 9 | Day 0 | 2.667* | 0.289 | <.001 | 1.881 | 3.453 |
|  |  | Day 3 | 2.000* | 0.289 | <.001 | 1.214 | 2.786 |
|  |  | Day 6 | 1.000* | 0.289 | 0.006 | 0.214 | 1.786 |
| Based on estimated marginal means | | | |  |  |  |  |
| * The mean difference is significant at the .05 level. | | | | |  |  |  |
| b Adjustment for multiple comparisons: Bonferroni. | | | | | |  |  |

| Univariate Tests | | | | | | | |
| --- | --- | --- | --- | --- | --- | --- | --- |
| Dependent Variable: SSC | | | | | | | |
| SNP  (µM) |  | Sum of Squares | df | Mean Square | F | Sig. | Partial Eta Squared |
| 0 | Contrast | 29.125 | 3 | 9.708 | 38.833 | <.001 | 0.645 |
|  | Error | 16 | 64 | 0.25 |  |  |  |
| 100 | Contrast | 24.125 | 3 | 8.042 | 32.167 | <.001 | 0.601 |
|  | Error | 16 | 64 | 0.25 |  |  |  |
| 200 | Contrast | 17.125 | 3 | 5.708 | 22.833 | <.001 | 0.517 |
|  | Error | 16 | 64 | 0.25 |  |  |  |
| 300 | Contrast | 24.5 | 3 | 8.167 | 32.667 | <.001 | 0.605 |
|  | Error | 16 | 64 | 0.25 |  |  |  |
| Each F tests the simple effects of Day within each level combination of the other effects shown. These tests are based on the linearly independent pairwise comparisons among the estimated marginal means. | | | | | | | |

**Post Hoc Tests**

| SSC |  |  |  |
| --- | --- | --- | --- |
|  | SNP (µM) | N | Subset |
|  |  |  | 1 |
| Duncana,b | 200 | 24 | 2.0417 |
|  | 0 | 24 | 2.125 |
|  | 300 | 24 | 2.25 |
|  | 100 | 24 | 2.2917 |
|  | Sig. |  | 0.119 |
| Means for groups in homogeneous subsets are displayed. | | | |
| Based on observed means. | | |  |
| The error term is Mean Square(Error) = .250. | | | |
| a Uses Harmonic Mean Sample Size = 24.000. | | | |
| b Alpha = .05. | |  |  |

| SSC |  |  |  |  |  |  |
| --- | --- | --- | --- | --- | --- | --- |
|  | Day | N | Subset |  |  |  |
|  |  |  | 1 | 2 | 3 | 4 |
| Duncana,b | Day 0 | 24 | 1 |  |  |  |
|  | Day 3 | 24 |  | 1.5417 |  |  |
|  | Day 6 | 24 |  |  | 2.625 |  |
|  | Day 9 | 24 |  |  |  | 3.5417 |
|  | Sig. |  | 1 | 1 | 1 | 1 |
| Means for groups in homogeneous subsets are displayed. | | | | | |  |
| Based on observed means. | | |  |  |  |  |
| The error term is Mean Square (Error) = .250. | | | | |  |  |
| a Uses Harmonic Mean Sample Size = 24.000. | | | | |  |  |
| b Alpha = .05. | |  |  |  |  |  |

**Electrolyte leakage**

| Tests of Between-Subjects Effects | | | | | | |
| --- | --- | --- | --- | --- | --- | --- |
| Dependent Variable: Electrolyte leakage | | | | | | |
| Source | Type III Sum of Squares | df | Mean Square | F | Sig. | Partial Eta Squared |
| Corrected Model | 2813.652^a^ | 31 | 90.763 | 64.484 | <.001 | .969 |
| Intercept | 56430.722 | 1 | 56430.722 | 40092.371 | <.001 | .998 |
| Light | 1.387 | 1 | 1.387 | .986 | .325 | .015 |
| SNP | 162.397 | 3 | 54.132 | 38.459 | <.001 | .643 |
| Day | 2484.345 | 3 | 828.115 | 588.351 | <.001 | .965 |
| Light * SNP | 28.192 | 3 | 9.397 | 6.677 | <.001 | .238 |
| Light * Day | 24.578 | 3 | 8.193 | 5.821 | .001 | .214 |
| SNP * Day | 94.556 | 9 | 10.506 | 7.464 | <.001 | .512 |
| Light * SNP * Day | 18.197 | 9 | 2.022 | 1.436 | .192 | .168 |
| Error | 90.081 | 64 | 1.408 |  |  |  |
| Total | 59334.455 | 96 |  |  |  |  |
| Corrected Total | 2903.733 | 95 |  |  |  |  |
| a. R Squared = .969 (Adjusted R Squared = .954) | | | | | | |

**Light * SNP**

| Pairwise Comparisons | | | | | | | |
| --- | --- | --- | --- | --- | --- | --- | --- |
| Dependent Variable: Electrolyte leakage | | | | | | | |
| SNP (µM) | (I) Light | (J) Light | Mean Difference (I-J) | Std. Error | Sig.^b^ | 95% Confidence Interval for Difference^b^ | |
|  |  |  |  |  |  | Lower Bound | Upper Bound |
| 0 | White light | High blue light | 1.482^*^ | .484 | .003 | .515 | 2.450 |
|  | High blue light | White light | -1.482^*^ | .484 | .003 | -2.450 | -.515 |
| 100 | White light | High blue light | .358 | .484 | .462 | -.609 | 1.326 |
|  | High blue light | White light | -.358 | .484 | .462 | -1.326 | .609 |
| 200 | White light | High blue light | -1.493^*^ | .484 | .003 | -2.460 | -.525 |
|  | High blue light | White light | 1.493^*^ | .484 | .003 | .525 | 2.460 |
| 300 | White light | High blue light | .613 | .484 | .210 | -.354 | 1.581 |
|  | High blue light | White light | -.613 | .484 | .210 | -1.581 | .354 |
| Based on estimated marginal means | | | | | | | |
| *. The mean difference is significant at the .05 level. | | | | | | | |
| b. Adjustment for multiple comparisons: Bonferroni. | | | | | | | |

| Univariate Tests | | | | | | | |
| --- | --- | --- | --- | --- | --- | --- | --- |
| Dependent Variable: Electrolyte leakage | | | | | | | |
| SNP (µM) | | Sum of Squares | df | Mean Square | F | Sig. | Partial Eta Squared |
| 0 | Contrast | 13.187 | 1 | 13.187 | 9.369 | .003 | .128 |
|  | Error | 90.081 | 64 | 1.408 |  |  |  |
| 100 | Contrast | .770 | 1 | .770 | .547 | .462 | .008 |
|  | Error | 90.081 | 64 | 1.408 |  |  |  |
| 200 | Contrast | 13.365 | 1 | 13.365 | 9.496 | .003 | .129 |
|  | Error | 90.081 | 64 | 1.408 |  |  |  |
| 300 | Contrast | 2.257 | 1 | 2.257 | 1.604 | .210 | .024 |
|  | Error | 90.081 | 64 | 1.408 |  |  |  |
| Each F tests the simple effects of Light within each level combination of the other effects shown. These tests are based on the linearly independent pairwise comparisons among the estimated marginal means. | | | | | | | |

| Pairwise Comparisons | | | | | | | |
| --- | --- | --- | --- | --- | --- | --- | --- |
| Dependent Variable: Electrolyte leakage | | | | | | | |
| Light | (I) SNP (µM) | (J) SNP (µM) | Mean Difference (I-J) | Std. Error | Sig.^b^ | 95% Confidence Interval for Difference^b^ | |
|  |  |  |  |  |  | Lower Bound | Upper Bound |
| White light | 0 | 100 | 2.702^*^ | .484 | <.001 | 1.384 | 4.021 |
|  |  | 200 | 5.111^*^ | .484 | <.001 | 3.792 | 6.430 |
|  |  | 300 | 2.772^*^ | .484 | <.001 | 1.454 | 4.091 |
|  | 100 | 0 | -2.702^*^ | .484 | <.001 | -4.021 | -1.384 |
|  |  | 200 | 2.408^*^ | .484 | <.001 | 1.090 | 3.727 |
|  |  | 300 | .070 | .484 | 1.000 | -1.249 | 1.389 |
|  | 200 | 0 | -5.111^*^ | .484 | <.001 | -6.430 | -3.792 |
|  |  | 100 | -2.408^*^ | .484 | <.001 | -3.727 | -1.090 |
|  |  | 300 | -2.338^*^ | .484 | <.001 | -3.657 | -1.020 |
|  | 300 | 0 | -2.772^*^ | .484 | <.001 | -4.091 | -1.454 |
|  |  | 100 | -.070 | .484 | 1.000 | -1.389 | 1.249 |
|  |  | 200 | 2.338^*^ | .484 | <.001 | 1.020 | 3.657 |
| High blue light | 0 | 100 | 1.578^*^ | .484 | .011 | .260 | 2.897 |
|  |  | 200 | 2.136^*^ | .484 | <.001 | .817 | 3.455 |
|  |  | 300 | 1.903^*^ | .484 | .001 | .585 | 3.222 |
|  | 100 | 0 | -1.578^*^ | .484 | .011 | -2.897 | -.260 |
|  |  | 200 | .557 | .484 | 1.000 | -.761 | 1.876 |
|  |  | 300 | .325 | .484 | 1.000 | -.994 | 1.644 |
|  | 200 | 0 | -2.136^*^ | .484 | <.001 | -3.455 | -.817 |
|  |  | 100 | -.557 | .484 | 1.000 | -1.876 | .761 |
|  |  | 300 | -.232 | .484 | 1.000 | -1.551 | 1.086 |
|  | 300 | 0 | -1.903^*^ | .484 | .001 | -3.222 | -.585 |
|  |  | 100 | -.325 | .484 | 1.000 | -1.644 | .994 |
|  |  | 200 | .232 | .484 | 1.000 | -1.086 | 1.551 |
| Based on estimated marginal means | | | | | | | |
| *. The mean difference is significant at the .05 level. | | | | | | | |
| b. Adjustment for multiple comparisons: Bonferroni. | | | | | | | |

| Univariate Tests | | | | | | | |
| --- | --- | --- | --- | --- | --- | --- | --- |
| Dependent Variable: Electrolyte leakage | | | | | | | |
| Light | | Sum of Squares | df | Mean Square | F | Sig. | Partial Eta Squared |
| White light | Contrast | 157.151 | 3 | 52.384 | 37.217 | <.001 | .636 |
|  | Error | 90.081 | 64 | 1.408 |  |  |  |
| High blue light | Contrast | 33.438 | 3 | 11.146 | 7.919 | <.001 | .271 |
|  | Error | 90.081 | 64 | 1.408 |  |  |  |
| Each F tests the simple effects of SNP within each level combination of the other effects shown. These tests are based on the linearly independent pairwise comparisons among the estimated marginal means. | | | | | | | |

**Light * Day**

| Pairwise Comparisons | | | | | | | |
| --- | --- | --- | --- | --- | --- | --- | --- |
| Dependent Variable: Electrolyte leakage | | | | | | | |
| Day | (I) Light | (J) Light | Mean Difference (I-J) | Std. Error | Sig.^b^ | 95% Confidence Interval for Difference^b^ | |
|  |  |  |  |  |  | Lower Bound | Upper Bound |
| Day 0 | White light | High blue light | -.987^*^ | .484 | .046 | -1.954 | -.019 |
|  | High blue light | White light | .987^*^ | .484 | .046 | .019 | 1.954 |
| Day 3 | White light | High blue light | -.307 | .484 | .528 | -1.275 | .660 |
|  | High blue light | White light | .307 | .484 | .528 | -.660 | 1.275 |
| Day 6 | White light | High blue light | .530 | .484 | .278 | -.438 | 1.498 |
|  | High blue light | White light | -.530 | .484 | .278 | -1.498 | .438 |
| Day 9 | White light | High blue light | 1.726^*^ | .484 | <.001 | .758 | 2.693 |
|  | High blue light | White light | -1.726^*^ | .484 | <.001 | -2.693 | -.758 |
| Based on estimated marginal means | | | | | | | |
| *. The mean difference is significant at the .05 level. | | | | | | | |
| b. Adjustment for multiple comparisons: Bonferroni. | | | | | | | |

| Univariate Tests | | | | | | | |
| --- | --- | --- | --- | --- | --- | --- | --- |
| Dependent Variable: Electrolyte leakage | | | | | | | |
| Day | | Sum of Squares | df | Mean Square | F | Sig. | Partial Eta Squared |
| Day 0 | Contrast | 5.841 | 1 | 5.841 | 4.150 | .046 | .061 |
|  | Error | 90.081 | 64 | 1.408 |  |  |  |
| Day 3 | Contrast | .567 | 1 | .567 | .403 | .528 | .006 |
|  | Error | 90.081 | 64 | 1.408 |  |  |  |
| Day 6 | Contrast | 1.685 | 1 | 1.685 | 1.197 | .278 | .018 |
|  | Error | 90.081 | 64 | 1.408 |  |  |  |
| Day 9 | Contrast | 17.871 | 1 | 17.871 | 12.697 | <.001 | .166 |
|  | Error | 90.081 | 64 | 1.408 |  |  |  |
| Each F tests the simple effects of Light within each level combination of the other effects shown. These tests are based on the linearly independent pairwise comparisons among the estimated marginal means. | | | | | | | |

| Pairwise Comparisons | | | | | | | |
| --- | --- | --- | --- | --- | --- | --- | --- |
| Dependent Variable: Electrolyte leakage | | | | | | | |
| Light | (I) Day | (J) Day | Mean Difference (I-J) | Std. Error | Sig.^b^ | 95% Confidence Interval for Difference^b^ | |
|  |  |  |  |  |  | Lower Bound | Upper Bound |
| White light | Day 0 | Day 3 | -5.918^*^ | .484 | <.001 | -7.236 | -4.599 |
|  |  | Day 6 | -9.242^*^ | .484 | <.001 | -10.560 | -7.923 |
|  |  | Day 9 | -15.448^*^ | .484 | <.001 | -16.767 | -14.130 |
|  | Day 3 | Day 0 | 5.918^*^ | .484 | <.001 | 4.599 | 7.236 |
|  |  | Day 6 | -3.324^*^ | .484 | <.001 | -4.643 | -2.005 |
|  |  | Day 9 | -9.531^*^ | .484 | <.001 | -10.850 | -8.212 |
|  | Day 6 | Day 0 | 9.242^*^ | .484 | <.001 | 7.923 | 10.560 |
|  |  | Day 3 | 3.324^*^ | .484 | <.001 | 2.005 | 4.643 |
|  |  | Day 9 | -6.207^*^ | .484 | <.001 | -7.525 | -4.888 |
|  | Day 9 | Day 0 | 15.448^*^ | .484 | <.001 | 14.130 | 16.767 |
|  |  | Day 3 | 9.531^*^ | .484 | <.001 | 8.212 | 10.850 |
|  |  | Day 6 | 6.207^*^ | .484 | <.001 | 4.888 | 7.525 |
| High blue light | Day 0 | Day 3 | -5.238^*^ | .484 | <.001 | -6.557 | -3.920 |
|  |  | Day 6 | -7.725^*^ | .484 | <.001 | -9.044 | -6.406 |
|  |  | Day 9 | -12.736^*^ | .484 | <.001 | -14.055 | -11.417 |
|  | Day 3 | Day 0 | 5.238^*^ | .484 | <.001 | 3.920 | 6.557 |
|  |  | Day 6 | -2.487^*^ | .484 | <.001 | -3.805 | -1.168 |
|  |  | Day 9 | -7.497^*^ | .484 | <.001 | -8.816 | -6.179 |
|  | Day 6 | Day 0 | 7.725^*^ | .484 | <.001 | 6.406 | 9.044 |
|  |  | Day 3 | 2.487^*^ | .484 | <.001 | 1.168 | 3.805 |
|  |  | Day 9 | -5.011^*^ | .484 | <.001 | -6.330 | -3.692 |
|  | Day 9 | Day 0 | 12.736^*^ | .484 | <.001 | 11.417 | 14.055 |
|  |  | Day 3 | 7.497^*^ | .484 | <.001 | 6.179 | 8.816 |
|  |  | Day 6 | 5.011^*^ | .484 | <.001 | 3.692 | 6.330 |
| Based on estimated marginal means | | | | | | | |
| *. The mean difference is significant at the .05 level. | | | | | | | |
| b. Adjustment for multiple comparisons: Bonferroni. | | | | | | | |

| Univariate Tests | | | | | | | |
| --- | --- | --- | --- | --- | --- | --- | --- |
| Dependent Variable: Electrolyte leakage | | | | | | | |
| Light | | Sum of Squares | df | Mean Square | F | Sig. | Partial Eta Squared |
| White light | Contrast | 1498.457 | 3 | 499.486 | 354.870 | <.001 | .943 |
|  | Error | 90.081 | 64 | 1.408 |  |  |  |
| High blue light | Contrast | 1010.465 | 3 | 336.822 | 239.302 | <.001 | .918 |
|  | Error | 90.081 | 64 | 1.408 |  |  |  |
| Each F tests the simple effects of Day within each level combination of the other effects shown. These tests are based on the linearly independent pairwise comparisons among the estimated marginal means. | | | | | | | |

**SNP * Day**

| Pairwise Comparisons | | | | | | | |
| --- | --- | --- | --- | --- | --- | --- | --- |
| Dependent Variable: Electrolyte leakage | | | | | | | |
| Day | (I) SNP  (µM) | (J) SNP  (µM) | Mean Difference (I-J) | Std. Error | Sig.^b^ | 95% Confidence Interval for Difference^b^ | |
|  |  |  |  |  |  | Lower Bound | Upper Bound |
| Day 0 | 0 | 100 | -2.220E-16 | .685 | 1.000 | -1.865 | 1.865 |
|  |  | 200 | -4.441E-16 | .685 | 1.000 | -1.865 | 1.865 |
|  |  | 300 | -6.939E-16 | .685 | 1.000 | -1.865 | 1.865 |
|  | 100 | 0 | 2.220E-16 | .685 | 1.000 | -1.865 | 1.865 |
|  |  | 200 | -4.441E-16 | .685 | 1.000 | -1.865 | 1.865 |
|  |  | 300 | -4.441E-16 | .685 | 1.000 | -1.865 | 1.865 |
|  | 200 | 0 | 4.441E-16 | .685 | 1.000 | -1.865 | 1.865 |
|  |  | 100 | 4.441E-16 | .685 | 1.000 | -1.865 | 1.865 |
|  |  | 300 | -4.441E-16 | .685 | 1.000 | -1.865 | 1.865 |
|  | 300 | 0 | 6.939E-16 | .685 | 1.000 | -1.865 | 1.865 |
|  |  | 100 | 4.441E-16 | .685 | 1.000 | -1.865 | 1.865 |
|  |  | 200 | 4.441E-16 | .685 | 1.000 | -1.865 | 1.865 |
| Day 3 | 0 | 100 | 3.968^*^ | .685 | <.001 | 2.103 | 5.833 |
|  |  | 200 | 5.540^*^ | .685 | <.001 | 3.675 | 7.405 |
|  |  | 300 | 5.113^*^ | .685 | <.001 | 3.248 | 6.978 |
|  | 100 | 0 | -3.968^*^ | .685 | <.001 | -5.833 | -2.103 |
|  |  | 200 | 1.572 | .685 | .150 | -.293 | 3.437 |
|  |  | 300 | 1.145 | .685 | .597 | -.720 | 3.010 |
|  | 200 | 0 | -5.540^*^ | .685 | <.001 | -7.405 | -3.675 |
|  |  | 100 | -1.572 | .685 | .150 | -3.437 | .293 |
|  |  | 300 | -.427 | .685 | 1.000 | -2.292 | 1.438 |
|  | 300 | 0 | -5.113^*^ | .685 | <.001 | -6.978 | -3.248 |
|  |  | 100 | -1.145 | .685 | .597 | -3.010 | .720 |
|  |  | 200 | .427 | .685 | 1.000 | -1.438 | 2.292 |
| Day 6 | 0 | 100 | 1.288 | .685 | .387 | -.577 | 3.153 |
|  |  | 200 | 3.315^*^ | .685 | <.001 | 1.450 | 5.180 |
|  |  | 300 | 2.557^*^ | .685 | .002 | .692 | 4.422 |
|  | 100 | 0 | -1.288 | .685 | .387 | -3.153 | .577 |
|  |  | 200 | 2.027^*^ | .685 | .026 | .162 | 3.892 |
|  |  | 300 | 1.268 | .685 | .412 | -.597 | 3.133 |
|  | 200 | 0 | -3.315^*^ | .685 | <.001 | -5.180 | -1.450 |
|  |  | 100 | -2.027^*^ | .685 | .026 | -3.892 | -.162 |
|  |  | 300 | -.758 | .685 | 1.000 | -2.623 | 1.107 |
|  | 300 | 0 | -2.557^*^ | .685 | .002 | -4.422 | -.692 |
|  |  | 100 | -1.268 | .685 | .412 | -3.133 | .597 |
|  |  | 200 | .758 | .685 | 1.000 | -1.107 | 2.623 |
| Day 9 | 0 | 100 | 3.305^*^ | .685 | <.001 | 1.440 | 5.170 |
|  |  | 200 | 5.638^*^ | .685 | <.001 | 3.773 | 7.503 |
|  |  | 300 | 1.682 | .685 | .101 | -.183 | 3.547 |
|  | 100 | 0 | -3.305^*^ | .685 | <.001 | -5.170 | -1.440 |
|  |  | 200 | 2.333^*^ | .685 | .007 | .468 | 4.198 |
|  |  | 300 | -1.623 | .685 | .125 | -3.488 | .242 |
|  | 200 | 0 | -5.638^*^ | .685 | <.001 | -7.503 | -3.773 |
|  |  | 100 | -2.333^*^ | .685 | .007 | -4.198 | -.468 |
|  |  | 300 | -3.957^*^ | .685 | <.001 | -5.822 | -2.092 |
|  | 300 | 0 | -1.682 | .685 | .101 | -3.547 | .183 |
|  |  | 100 | 1.623 | .685 | .125 | -.242 | 3.488 |
|  |  | 200 | 3.957^*^ | .685 | <.001 | 2.092 | 5.822 |
| Based on estimated marginal means | | | | | | | |
| *. The mean difference is significant at the .05 level. | | | | | | | |
| b. Adjustment for multiple comparisons: Bonferroni. | | | | | | | |

| Univariate Tests | | | | | | | |
| --- | --- | --- | --- | --- | --- | --- | --- |
| Dependent Variable: Electrolyte leakage | | | | | | | |
| Day | | Sum of Squares | df | Mean Square | F | Sig. | Partial Eta Squared |
| Day 0 | Contrast | 1.594E-30 | 3 | 5.312E-31 | .000 | 1.000 | .000 |
|  | Error | 90.081 | 64 | 1.408 |  |  |  |
| Day 3 | Contrast | 114.823 | 3 | 38.274 | 27.193 | <.001 | .560 |
|  | Error | 90.081 | 64 | 1.408 |  |  |  |
| Day 6 | Contrast | 38.215 | 3 | 12.738 | 9.050 | <.001 | .298 |
|  | Error | 90.081 | 64 | 1.408 |  |  |  |
| Day 9 | Contrast | 103.915 | 3 | 34.638 | 24.610 | <.001 | .536 |
|  | Error | 90.081 | 64 | 1.408 |  |  |  |
| Each F tests the simple effects of SNP within each level combination of the other effects shown. These tests are based on the linearly independent pairwise comparisons among the estimated marginal means. | | | | | | | |

| Pairwise Comparisons | | | | | | | |
| --- | --- | --- | --- | --- | --- | --- | --- |
| Dependent Variable: Electrolyte leakage | | | | | | | |
| SNP (µM) | (I) Day | (J) Day | Mean Difference (I-J) | Std. Error | Sig.^b^ | 95% Confidence Interval for Difference^b^ | |
|  |  |  |  |  |  | Lower Bound | Upper Bound |
| 0 | Day 0 | Day 3 | -9.233^*^ | .685 | <.001 | -11.098 | -7.368 |
|  |  | Day 6 | -10.273^*^ | .685 | <.001 | -12.138 | -8.408 |
|  |  | Day 9 | -16.748^*^ | .685 | <.001 | -18.613 | -14.883 |
|  | Day 3 | Day 0 | 9.233^*^ | .685 | <.001 | 7.368 | 11.098 |
|  |  | Day 6 | -1.040 | .685 | .803 | -2.905 | .825 |
|  |  | Day 9 | -7.515^*^ | .685 | <.001 | -9.380 | -5.650 |
|  | Day 6 | Day 0 | 10.273^*^ | .685 | <.001 | 8.408 | 12.138 |
|  |  | Day 3 | 1.040 | .685 | .803 | -.825 | 2.905 |
|  |  | Day 9 | -6.475^*^ | .685 | <.001 | -8.340 | -4.610 |
|  | Day 9 | Day 0 | 16.748^*^ | .685 | <.001 | 14.883 | 18.613 |
|  |  | Day 3 | 7.515^*^ | .685 | <.001 | 5.650 | 9.380 |
|  |  | Day 6 | 6.475^*^ | .685 | <.001 | 4.610 | 8.340 |
| 100 | Day 0 | Day 3 | -5.265^*^ | .685 | <.001 | -7.130 | -3.400 |
|  |  | Day 6 | -8.985^*^ | .685 | <.001 | -10.850 | -7.120 |
|  |  | Day 9 | -13.443^*^ | .685 | <.001 | -15.308 | -11.578 |
|  | Day 3 | Day 0 | 5.265^*^ | .685 | <.001 | 3.400 | 7.130 |
|  |  | Day 6 | -3.720^*^ | .685 | <.001 | -5.585 | -1.855 |
|  |  | Day 9 | -8.178^*^ | .685 | <.001 | -10.043 | -6.313 |
|  | Day 6 | Day 0 | 8.985^*^ | .685 | <.001 | 7.120 | 10.850 |
|  |  | Day 3 | 3.720^*^ | .685 | <.001 | 1.855 | 5.585 |
|  |  | Day 9 | -4.458^*^ | .685 | <.001 | -6.323 | -2.593 |
|  | Day 9 | Day 0 | 13.443^*^ | .685 | <.001 | 11.578 | 15.308 |
|  |  | Day 3 | 8.178^*^ | .685 | <.001 | 6.313 | 10.043 |
|  |  | Day 6 | 4.458^*^ | .685 | <.001 | 2.593 | 6.323 |
| 200 | Day 0 | Day 3 | -3.693^*^ | .685 | <.001 | -5.558 | -1.828 |
|  |  | Day 6 | -6.958^*^ | .685 | <.001 | -8.823 | -5.093 |
|  |  | Day 9 | -11.110^*^ | .685 | <.001 | -12.975 | -9.245 |
|  | Day 3 | Day 0 | 3.693^*^ | .685 | <.001 | 1.828 | 5.558 |
|  |  | Day 6 | -3.265^*^ | .685 | <.001 | -5.130 | -1.400 |
|  |  | Day 9 | -7.417^*^ | .685 | <.001 | -9.282 | -5.552 |
|  | Day 6 | Day 0 | 6.958^*^ | .685 | <.001 | 5.093 | 8.823 |
|  |  | Day 3 | 3.265^*^ | .685 | <.001 | 1.400 | 5.130 |
|  |  | Day 9 | -4.152^*^ | .685 | <.001 | -6.017 | -2.287 |
|  | Day 9 | Day 0 | 11.110^*^ | .685 | <.001 | 9.245 | 12.975 |
|  |  | Day 3 | 7.417^*^ | .685 | <.001 | 5.552 | 9.282 |
|  |  | Day 6 | 4.152^*^ | .685 | <.001 | 2.287 | 6.017 |
| 300 | Day 0 | Day 3 | -4.120^*^ | .685 | <.001 | -5.985 | -2.255 |
|  |  | Day 6 | -7.717^*^ | .685 | <.001 | -9.582 | -5.852 |
|  |  | Day 9 | -15.067^*^ | .685 | <.001 | -16.932 | -13.202 |
|  | Day 3 | Day 0 | 4.120^*^ | .685 | <.001 | 2.255 | 5.985 |
|  |  | Day 6 | -3.597^*^ | .685 | <.001 | -5.462 | -1.732 |
|  |  | Day 9 | -10.947^*^ | .685 | <.001 | -12.812 | -9.082 |
|  | Day 6 | Day 0 | 7.717^*^ | .685 | <.001 | 5.852 | 9.582 |
|  |  | Day 3 | 3.597^*^ | .685 | <.001 | 1.732 | 5.462 |
|  |  | Day 9 | -7.350^*^ | .685 | <.001 | -9.215 | -5.485 |
|  | Day 9 | Day 0 | 15.067^*^ | .685 | <.001 | 13.202 | 16.932 |
|  |  | Day 3 | 10.947^*^ | .685 | <.001 | 9.082 | 12.812 |
|  |  | Day 6 | 7.350^*^ | .685 | <.001 | 5.485 | 9.215 |
| Based on estimated marginal means | | | | | | | |
| *. The mean difference is significant at the .05 level. | | | | | | | |
| b. Adjustment for multiple comparisons: Bonferroni. | | | | | | | |

| Univariate Tests | | | | | | | |
| --- | --- | --- | --- | --- | --- | --- | --- |
| Dependent Variable: Electrolyte leakage | | | | | | | |
| SNP (µM) | | Sum of Squares | df | Mean Square | F | Sig. | Partial Eta Squared |
| 0 | Contrast | 856.177 | 3 | 285.392 | 202.763 | <.001 | .905 |
|  | Error | 90.081 | 64 | 1.408 |  |  |  |
| 100 | Contrast | 584.661 | 3 | 194.887 | 138.461 | <.001 | .866 |
|  | Error | 90.081 | 64 | 1.408 |  |  |  |
| 200 | Contrast | 402.592 | 3 | 134.197 | 95.343 | <.001 | .817 |
|  | Error | 90.081 | 64 | 1.408 |  |  |  |
| 300 | Contrast | 735.471 | 3 | 245.157 | 174.177 | <.001 | .891 |
|  | Error | 90.081 | 64 | 1.408 |  |  |  |
| Each F tests the simple effects of Day within each level combination of the other effects shown. These tests are based on the linearly independent pairwise comparisons among the estimated marginal means. | | | | | | | |

**Light * SNP * Day**

| Pairwise Comparisons | | | | | | | | |
| --- | --- | --- | --- | --- | --- | --- | --- | --- |
| Dependent Variable: Electrolyte leakage | | | | | | | | |
| SNP (µM) | Day | (I) Light | (J) Light | Mean Difference (I-J) | Std. Error | Sig.^b^ | 95% Confidence Interval for Difference^b^ | |
|  |  |  |  |  |  |  | Lower Bound | Upper Bound |
| 0 | Day 0 | White light | High blue light | -.987 | .969 | .312 | -2.922 | .948 |
|  |  | High blue light | White light | .987 | .969 | .312 | -.948 | 2.922 |
|  | Day 3 | White light | High blue light | 2.300^*^ | .969 | .021 | .365 | 4.235 |
|  |  | High blue light | White light | -2.300^*^ | .969 | .021 | -4.235 | -.365 |
|  | Day 6 | White light | High blue light | 2.220^*^ | .969 | .025 | .285 | 4.155 |
|  |  | High blue light | White light | -2.220^*^ | .969 | .025 | -4.155 | -.285 |
|  | Day 9 | White light | High blue light | 2.397^*^ | .969 | .016 | .462 | 4.332 |
|  |  | High blue light | White light | -2.397^*^ | .969 | .016 | -4.332 | -.462 |
| 100 | Day 0 | White light | High blue light | -.987 | .969 | .312 | -2.922 | .948 |
|  |  | High blue light | White light | .987 | .969 | .312 | -.948 | 2.922 |
|  | Day 3 | White light | High blue light | -1.250 | .969 | .202 | -3.185 | .685 |
|  |  | High blue light | White light | 1.250 | .969 | .202 | -.685 | 3.185 |
|  | Day 6 | White light | High blue light | 1.510 | .969 | .124 | -.425 | 3.445 |
|  |  | High blue light | White light | -1.510 | .969 | .124 | -3.445 | .425 |
|  | Day 9 | White light | High blue light | 2.160^*^ | .969 | .029 | .225 | 4.095 |
|  |  | High blue light | White light | -2.160^*^ | .969 | .029 | -4.095 | -.225 |
| 200 | Day 0 | White light | High blue light | -.987 | .969 | .312 | -2.922 | .948 |
|  |  | High blue light | White light | .987 | .969 | .312 | -.948 | 2.922 |
|  | Day 3 | White light | High blue light | -2.360^*^ | .969 | .018 | -4.295 | -.425 |
|  |  | High blue light | White light | 2.360^*^ | .969 | .018 | .425 | 4.295 |
|  | Day 6 | White light | High blue light | -2.870^*^ | .969 | .004 | -4.805 | -.935 |
|  |  | High blue light | White light | 2.870^*^ | .969 | .004 | .935 | 4.805 |
|  | Day 9 | White light | High blue light | .247 | .969 | .800 | -1.688 | 2.182 |
|  |  | High blue light | White light | -.247 | .969 | .800 | -2.182 | 1.688 |
| 300 | Day 0 | White light | High blue light | -.987 | .969 | .312 | -2.922 | .948 |
|  |  | High blue light | White light | .987 | .969 | .312 | -.948 | 2.922 |
|  | Day 3 | White light | High blue light | .080 | .969 | .934 | -1.855 | 2.015 |
|  |  | High blue light | White light | -.080 | .969 | .934 | -2.015 | 1.855 |
|  | Day 6 | White light | High blue light | 1.260 | .969 | .198 | -.675 | 3.195 |
|  |  | High blue light | White light | -1.260 | .969 | .198 | -3.195 | .675 |
|  | Day 9 | White light | High blue light | 2.100^*^ | .969 | .034 | .165 | 4.035 |
|  |  | High blue light | White light | -2.100^*^ | .969 | .034 | -4.035 | -.165 |
| Based on estimated marginal means | | | | | | | | |
| *. The mean difference is significant at the .05 level. | | | | | | | | |
| b. Adjustment for multiple comparisons: Bonferroni. | | | | | | | | |

| Univariate Tests | | | | | | | | |
| --- | --- | --- | --- | --- | --- | --- | --- | --- |
| Dependent Variable: Electrolyte leakage | | | | | | | | |
| SNP (µM) | Day | | Sum of Squares | df | Mean Square | F | Sig. | Partial Eta Squared |
| 0 | Day 0 | Contrast | 1.460 | 1 | 1.460 | 1.037 | .312 | .016 |
|  |  | Error | 90.081 | 64 | 1.408 |  |  |  |
|  | Day 3 | Contrast | 7.935 | 1 | 7.935 | 5.638 | .021 | .081 |
|  |  | Error | 90.081 | 64 | 1.408 |  |  |  |
|  | Day 6 | Contrast | 7.393 | 1 | 7.393 | 5.252 | .025 | .076 |
|  |  | Error | 90.081 | 64 | 1.408 |  |  |  |
|  | Day 9 | Contrast | 8.616 | 1 | 8.616 | 6.121 | .016 | .087 |
|  |  | Error | 90.081 | 64 | 1.408 |  |  |  |
| 100 | Day 0 | Contrast | 1.460 | 1 | 1.460 | 1.037 | .312 | .016 |
|  |  | Error | 90.081 | 64 | 1.408 |  |  |  |
|  | Day 3 | Contrast | 2.344 | 1 | 2.344 | 1.665 | .202 | .025 |
|  |  | Error | 90.081 | 64 | 1.408 |  |  |  |
|  | Day 6 | Contrast | 3.420 | 1 | 3.420 | 2.430 | .124 | .037 |
|  |  | Error | 90.081 | 64 | 1.408 |  |  |  |
|  | Day 9 | Contrast | 6.998 | 1 | 6.998 | 4.972 | .029 | .072 |
|  |  | Error | 90.081 | 64 | 1.408 |  |  |  |
| 200 | Day 0 | Contrast | 1.460 | 1 | 1.460 | 1.037 | .312 | .016 |
|  |  | Error | 90.081 | 64 | 1.408 |  |  |  |
|  | Day 3 | Contrast | 8.354 | 1 | 8.354 | 5.936 | .018 | .085 |
|  |  | Error | 90.081 | 64 | 1.408 |  |  |  |
|  | Day 6 | Contrast | 12.355 | 1 | 12.355 | 8.778 | .004 | .121 |
|  |  | Error | 90.081 | 64 | 1.408 |  |  |  |
|  | Day 9 | Contrast | .091 | 1 | .091 | .065 | .800 | .001 |
|  |  | Error | 90.081 | 64 | 1.408 |  |  |  |
| 300 | Day 0 | Contrast | 1.460 | 1 | 1.460 | 1.037 | .312 | .016 |
|  |  | Error | 90.081 | 64 | 1.408 |  |  |  |
|  | Day 3 | Contrast | .010 | 1 | .010 | .007 | .934 | .000 |
|  |  | Error | 90.081 | 64 | 1.408 |  |  |  |
|  | Day 6 | Contrast | 2.381 | 1 | 2.381 | 1.692 | .198 | .026 |
|  |  | Error | 90.081 | 64 | 1.408 |  |  |  |
|  | Day 9 | Contrast | 6.615 | 1 | 6.615 | 4.700 | .034 | .068 |
|  |  | Error | 90.081 | 64 | 1.408 |  |  |  |
| Each F tests the simple effects of Light within each level combination of the other effects shown. These tests are based on the linearly independent pairwise comparisons among the estimated marginal means. | | | | | | | | |

| Pairwise Comparisons | | | | | | | | |
| --- | --- | --- | --- | --- | --- | --- | --- | --- |
| Dependent Variable: Electrolyte leakage | | | | | | | | |
| Light | Day | (I) SNP (µM) | (J) SNP (µM) | Mean Difference (I-J) | Std. Error | Sig.^b^ | 95% Confidence Interval for Difference^b^ | |
|  |  |  |  |  |  |  | Lower Bound | Upper Bound |
| White light | Day 0 | 0 | 100 | .000 | .969 | 1.000 | -2.637 | 2.637 |
|  |  |  | 200 | .000 | .969 | 1.000 | -2.637 | 2.637 |
|  |  |  | 300 | -7.216E-16 | .969 | 1.000 | -2.637 | 2.637 |
|  |  | 100 | 0 | .000 | .969 | 1.000 | -2.637 | 2.637 |
|  |  |  | 200 | .000 | .969 | 1.000 | -2.637 | 2.637 |
|  |  |  | 300 | -6.661E-16 | .969 | 1.000 | -2.637 | 2.637 |
|  |  | 200 | 0 | .000 | .969 | 1.000 | -2.637 | 2.637 |
|  |  |  | 100 | .000 | .969 | 1.000 | -2.637 | 2.637 |
|  |  |  | 300 | -8.882E-16 | .969 | 1.000 | -2.637 | 2.637 |
|  |  | 300 | 0 | 7.216E-16 | .969 | 1.000 | -2.637 | 2.637 |
|  |  |  | 100 | 6.661E-16 | .969 | 1.000 | -2.637 | 2.637 |
|  |  |  | 200 | 8.882E-16 | .969 | 1.000 | -2.637 | 2.637 |
|  | Day 3 | 0 | 100 | 5.743^*^ | .969 | <.001 | 3.106 | 8.381 |
|  |  |  | 200 | 7.870^*^ | .969 | <.001 | 5.233 | 10.507 |
|  |  |  | 300 | 6.223^*^ | .969 | <.001 | 3.586 | 8.861 |
|  |  | 100 | 0 | -5.743^*^ | .969 | <.001 | -8.381 | -3.106 |
|  |  |  | 200 | 2.127 | .969 | .191 | -.511 | 4.764 |
|  |  |  | 300 | .480 | .969 | 1.000 | -2.157 | 3.117 |
|  |  | 200 | 0 | -7.870^*^ | .969 | <.001 | -10.507 | -5.233 |
|  |  |  | 100 | -2.127 | .969 | .191 | -4.764 | .511 |
|  |  |  | 300 | -1.647 | .969 | .564 | -4.284 | .991 |
|  |  | 300 | 0 | -6.223^*^ | .969 | <.001 | -8.861 | -3.586 |
|  |  |  | 100 | -.480 | .969 | 1.000 | -3.117 | 2.157 |
|  |  |  | 200 | 1.647 | .969 | .564 | -.991 | 4.284 |
|  | Day 6 | 0 | 100 | 1.643 | .969 | .568 | -.994 | 4.281 |
|  |  |  | 200 | 5.860^*^ | .969 | <.001 | 3.223 | 8.497 |
|  |  |  | 300 | 3.037^*^ | .969 | .016 | .399 | 5.674 |
|  |  | 100 | 0 | -1.643 | .969 | .568 | -4.281 | .994 |
|  |  |  | 200 | 4.217^*^ | .969 | <.001 | 1.579 | 6.854 |
|  |  |  | 300 | 1.393 | .969 | .931 | -1.244 | 4.031 |
|  |  | 200 | 0 | -5.860^*^ | .969 | <.001 | -8.497 | -3.223 |
|  |  |  | 100 | -4.217^*^ | .969 | <.001 | -6.854 | -1.579 |
|  |  |  | 300 | -2.823^*^ | .969 | .029 | -5.461 | -.186 |
|  |  | 300 | 0 | -3.037^*^ | .969 | .016 | -5.674 | -.399 |
|  |  |  | 100 | -1.393 | .969 | .931 | -4.031 | 1.244 |
|  |  |  | 200 | 2.823^*^ | .969 | .029 | .186 | 5.461 |
|  | Day 9 | 0 | 100 | 3.423^*^ | .969 | .005 | .786 | 6.061 |
|  |  |  | 200 | 6.713^*^ | .969 | <.001 | 4.076 | 9.351 |
|  |  |  | 300 | 1.830 | .969 | .380 | -.807 | 4.467 |
|  |  | 100 | 0 | -3.423^*^ | .969 | .005 | -6.061 | -.786 |
|  |  |  | 200 | 3.290^*^ | .969 | .007 | .653 | 5.927 |
|  |  |  | 300 | -1.593 | .969 | .629 | -4.231 | 1.044 |
|  |  | 200 | 0 | -6.713^*^ | .969 | <.001 | -9.351 | -4.076 |
|  |  |  | 100 | -3.290^*^ | .969 | .007 | -5.927 | -.653 |
|  |  |  | 300 | -4.883^*^ | .969 | <.001 | -7.521 | -2.246 |
|  |  | 300 | 0 | -1.830 | .969 | .380 | -4.467 | .807 |
|  |  |  | 100 | 1.593 | .969 | .629 | -1.044 | 4.231 |
|  |  |  | 200 | 4.883^*^ | .969 | <.001 | 2.246 | 7.521 |
| High blue light | Day 0 | 0 | 100 | -2.220E-16 | .969 | 1.000 | -2.637 | 2.637 |
|  |  |  | 200 | .000 | .969 | 1.000 | -2.637 | 2.637 |
|  |  |  | 300 | -6.661E-16 | .969 | 1.000 | -2.637 | 2.637 |
|  |  | 100 | 0 | 2.220E-16 | .969 | 1.000 | -2.637 | 2.637 |
|  |  |  | 200 | 2.220E-16 | .969 | 1.000 | -2.637 | 2.637 |
|  |  |  | 300 | -4.441E-16 | .969 | 1.000 | -2.637 | 2.637 |
|  |  | 200 | 0 | .000 | .969 | 1.000 | -2.637 | 2.637 |
|  |  |  | 100 | -2.220E-16 | .969 | 1.000 | -2.637 | 2.637 |
|  |  |  | 300 | -4.441E-16 | .969 | 1.000 | -2.637 | 2.637 |
|  |  | 300 | 0 | 6.661E-16 | .969 | 1.000 | -2.637 | 2.637 |
|  |  |  | 100 | 4.441E-16 | .969 | 1.000 | -2.637 | 2.637 |
|  |  |  | 200 | 4.441E-16 | .969 | 1.000 | -2.637 | 2.637 |
|  | Day 3 | 0 | 100 | 2.193 | .969 | .162 | -.444 | 4.831 |
|  |  |  | 200 | 3.210^*^ | .969 | .009 | .573 | 5.847 |
|  |  |  | 300 | 4.003^*^ | .969 | <.001 | 1.366 | 6.641 |
|  |  | 100 | 0 | -2.193 | .969 | .162 | -4.831 | .444 |
|  |  |  | 200 | 1.017 | .969 | 1.000 | -1.621 | 3.654 |
|  |  |  | 300 | 1.810 | .969 | .398 | -.827 | 4.447 |
|  |  | 200 | 0 | -3.210^*^ | .969 | .009 | -5.847 | -.573 |
|  |  |  | 100 | -1.017 | .969 | 1.000 | -3.654 | 1.621 |
|  |  |  | 300 | .793 | .969 | 1.000 | -1.844 | 3.431 |
|  |  | 300 | 0 | -4.003^*^ | .969 | <.001 | -6.641 | -1.366 |
|  |  |  | 100 | -1.810 | .969 | .398 | -4.447 | .827 |
|  |  |  | 200 | -.793 | .969 | 1.000 | -3.431 | 1.844 |
|  | Day 6 | 0 | 100 | .933 | .969 | 1.000 | -1.704 | 3.571 |
|  |  |  | 200 | .770 | .969 | 1.000 | -1.867 | 3.407 |
|  |  |  | 300 | 2.077 | .969 | .215 | -.561 | 4.714 |
|  |  | 100 | 0 | -.933 | .969 | 1.000 | -3.571 | 1.704 |
|  |  |  | 200 | -.163 | .969 | 1.000 | -2.801 | 2.474 |
|  |  |  | 300 | 1.143 | .969 | 1.000 | -1.494 | 3.781 |
|  |  | 200 | 0 | -.770 | .969 | 1.000 | -3.407 | 1.867 |
|  |  |  | 100 | .163 | .969 | 1.000 | -2.474 | 2.801 |
|  |  |  | 300 | 1.307 | .969 | 1.000 | -1.331 | 3.944 |
|  |  | 300 | 0 | -2.077 | .969 | .215 | -4.714 | .561 |
|  |  |  | 100 | -1.143 | .969 | 1.000 | -3.781 | 1.494 |
|  |  |  | 200 | -1.307 | .969 | 1.000 | -3.944 | 1.331 |
|  | Day 9 | 0 | 100 | 3.187^*^ | .969 | .010 | .549 | 5.824 |
|  |  |  | 200 | 4.563^*^ | .969 | <.001 | 1.926 | 7.201 |
|  |  |  | 300 | 1.533 | .969 | .710 | -1.104 | 4.171 |
|  |  | 100 | 0 | -3.187^*^ | .969 | .010 | -5.824 | -.549 |
|  |  |  | 200 | 1.377 | .969 | .961 | -1.261 | 4.014 |
|  |  |  | 300 | -1.653 | .969 | .556 | -4.291 | .984 |
|  |  | 200 | 0 | -4.563^*^ | .969 | <.001 | -7.201 | -1.926 |
|  |  |  | 100 | -1.377 | .969 | .961 | -4.014 | 1.261 |
|  |  |  | 300 | -3.030^*^ | .969 | .016 | -5.667 | -.393 |
|  |  | 300 | 0 | -1.533 | .969 | .710 | -4.171 | 1.104 |
|  |  |  | 100 | 1.653 | .969 | .556 | -.984 | 4.291 |
|  |  |  | 200 | 3.030^*^ | .969 | .016 | .393 | 5.667 |
| Based on estimated marginal means | | | | | | | | |
| *. The mean difference is significant at the .05 level. | | | | | | | | |
| b. Adjustment for multiple comparisons: Bonferroni. | | | | | | | | |

| Univariate Tests | | | | | | | | |
| --- | --- | --- | --- | --- | --- | --- | --- | --- |
| Dependent Variable: Electrolyte leakage | | | | | | | | |
| Light | Day | | Sum of Squares | df | Mean Square | F | Sig. | Partial Eta Squared |
| White light | Day 0 | Contrast | 1.172E-30 | 3 | 3.906E-31 | .000 | 1.000 | .000 |
|  |  | Error | 90.081 | 64 | 1.408 |  |  |  |
|  | Day 3 | Contrast | 105.838 | 3 | 35.279 | 25.065 | <.001 | .540 |
|  |  | Error | 90.081 | 64 | 1.408 |  |  |  |
|  | Day 6 | Contrast | 55.466 | 3 | 18.489 | 13.136 | <.001 | .381 |
|  |  | Error | 90.081 | 64 | 1.408 |  |  |  |
|  | Day 9 | Contrast | 73.010 | 3 | 24.337 | 17.290 | <.001 | .448 |
|  |  | Error | 90.081 | 64 | 1.408 |  |  |  |
| High blue light | Day 0 | Contrast | 8.875E-31 | 3 | 2.958E-31 | .000 | 1.000 | .000 |
|  |  | Error | 90.081 | 64 | 1.408 |  |  |  |
|  | Day 3 | Contrast | 27.060 | 3 | 9.020 | 6.409 | <.001 | .231 |
|  |  | Error | 90.081 | 64 | 1.408 |  |  |  |
|  | Day 6 | Contrast | 6.613 | 3 | 2.204 | 1.566 | .206 | .068 |
|  |  | Error | 90.081 | 64 | 1.408 |  |  |  |
|  | Day 9 | Contrast | 35.355 | 3 | 11.785 | 8.373 | <.001 | .282 |
|  |  | Error | 90.081 | 64 | 1.408 |  |  |  |
| Each F tests the simple effects of SNP within each level combination of the other effects shown. These tests are based on the linearly independent pairwise comparisons among the estimated marginal means. | | | | | | | | |

| Pairwise Comparisons | | | | | | | | |
| --- | --- | --- | --- | --- | --- | --- | --- | --- |
| Dependent Variable: Electrolyte leakage | | | | | | | | |
| Light | SNP (µM) | (I) Day | (J) Day | Mean Difference (I-J) | Std. Error | Sig.^b^ | 95% Confidence Interval for Difference^b^ | |
|  |  |  |  |  |  |  | Lower Bound | Upper Bound |
| White light | 0 | Day 0 | Day 3 | -10.877^*^ | .969 | <.001 | -13.514 | -8.239 |
|  |  |  | Day 6 | -11.877^*^ | .969 | <.001 | -14.514 | -9.239 |
|  |  |  | Day 9 | -18.440^*^ | .969 | <.001 | -21.077 | -15.803 |
|  |  | Day 3 | Day 0 | 10.877^*^ | .969 | <.001 | 8.239 | 13.514 |
|  |  |  | Day 6 | -1.000 | .969 | 1.000 | -3.637 | 1.637 |
|  |  |  | Day 9 | -7.563^*^ | .969 | <.001 | -10.201 | -4.926 |
|  |  | Day 6 | Day 0 | 11.877^*^ | .969 | <.001 | 9.239 | 14.514 |
|  |  |  | Day 3 | 1.000 | .969 | 1.000 | -1.637 | 3.637 |
|  |  |  | Day 9 | -6.563^*^ | .969 | <.001 | -9.201 | -3.926 |
|  |  | Day 9 | Day 0 | 18.440^*^ | .969 | <.001 | 15.803 | 21.077 |
|  |  |  | Day 3 | 7.563^*^ | .969 | <.001 | 4.926 | 10.201 |
|  |  |  | Day 6 | 6.563^*^ | .969 | <.001 | 3.926 | 9.201 |
|  | 100 | Day 0 | Day 3 | -5.133^*^ | .969 | <.001 | -7.771 | -2.496 |
|  |  |  | Day 6 | -10.233^*^ | .969 | <.001 | -12.871 | -7.596 |
|  |  |  | Day 9 | -15.017^*^ | .969 | <.001 | -17.654 | -12.379 |
|  |  | Day 3 | Day 0 | 5.133^*^ | .969 | <.001 | 2.496 | 7.771 |
|  |  |  | Day 6 | -5.100^*^ | .969 | <.001 | -7.737 | -2.463 |
|  |  |  | Day 9 | -9.883^*^ | .969 | <.001 | -12.521 | -7.246 |
|  |  | Day 6 | Day 0 | 10.233^*^ | .969 | <.001 | 7.596 | 12.871 |
|  |  |  | Day 3 | 5.100^*^ | .969 | <.001 | 2.463 | 7.737 |
|  |  |  | Day 9 | -4.783^*^ | .969 | <.001 | -7.421 | -2.146 |
|  |  | Day 9 | Day 0 | 15.017^*^ | .969 | <.001 | 12.379 | 17.654 |
|  |  |  | Day 3 | 9.883^*^ | .969 | <.001 | 7.246 | 12.521 |
|  |  |  | Day 6 | 4.783^*^ | .969 | <.001 | 2.146 | 7.421 |
|  | 200 | Day 0 | Day 3 | -3.007^*^ | .969 | .017 | -5.644 | -.369 |
|  |  |  | Day 6 | -6.017^*^ | .969 | <.001 | -8.654 | -3.379 |
|  |  |  | Day 9 | -11.727^*^ | .969 | <.001 | -14.364 | -9.089 |
|  |  | Day 3 | Day 0 | 3.007^*^ | .969 | .017 | .369 | 5.644 |
|  |  |  | Day 6 | -3.010^*^ | .969 | .017 | -5.647 | -.373 |
|  |  |  | Day 9 | -8.720^*^ | .969 | <.001 | -11.357 | -6.083 |
|  |  | Day 6 | Day 0 | 6.017^*^ | .969 | <.001 | 3.379 | 8.654 |
|  |  |  | Day 3 | 3.010^*^ | .969 | .017 | .373 | 5.647 |
|  |  |  | Day 9 | -5.710^*^ | .969 | <.001 | -8.347 | -3.073 |
|  |  | Day 9 | Day 0 | 11.727^*^ | .969 | <.001 | 9.089 | 14.364 |
|  |  |  | Day 3 | 8.720^*^ | .969 | <.001 | 6.083 | 11.357 |
|  |  |  | Day 6 | 5.710^*^ | .969 | <.001 | 3.073 | 8.347 |
|  | 300 | Day 0 | Day 3 | -4.653^*^ | .969 | <.001 | -7.291 | -2.016 |
|  |  |  | Day 6 | -8.840^*^ | .969 | <.001 | -11.477 | -6.203 |
|  |  |  | Day 9 | -16.610^*^ | .969 | <.001 | -19.247 | -13.973 |
|  |  | Day 3 | Day 0 | 4.653^*^ | .969 | <.001 | 2.016 | 7.291 |
|  |  |  | Day 6 | -4.187^*^ | .969 | <.001 | -6.824 | -1.549 |
|  |  |  | Day 9 | -11.957^*^ | .969 | <.001 | -14.594 | -9.319 |
|  |  | Day 6 | Day 0 | 8.840^*^ | .969 | <.001 | 6.203 | 11.477 |
|  |  |  | Day 3 | 4.187^*^ | .969 | <.001 | 1.549 | 6.824 |
|  |  |  | Day 9 | -7.770^*^ | .969 | <.001 | -10.407 | -5.133 |
|  |  | Day 9 | Day 0 | 16.610^*^ | .969 | <.001 | 13.973 | 19.247 |
|  |  |  | Day 3 | 11.957^*^ | .969 | <.001 | 9.319 | 14.594 |
|  |  |  | Day 6 | 7.770^*^ | .969 | <.001 | 5.133 | 10.407 |
| High blue light | 0 | Day 0 | Day 3 | -7.590^*^ | .969 | <.001 | -10.227 | -4.953 |
|  |  |  | Day 6 | -8.670^*^ | .969 | <.001 | -11.307 | -6.033 |
|  |  |  | Day 9 | -15.057^*^ | .969 | <.001 | -17.694 | -12.419 |
|  |  | Day 3 | Day 0 | 7.590^*^ | .969 | <.001 | 4.953 | 10.227 |
|  |  |  | Day 6 | -1.080 | .969 | 1.000 | -3.717 | 1.557 |
|  |  |  | Day 9 | -7.467^*^ | .969 | <.001 | -10.104 | -4.829 |
|  |  | Day 6 | Day 0 | 8.670^*^ | .969 | <.001 | 6.033 | 11.307 |
|  |  |  | Day 3 | 1.080 | .969 | 1.000 | -1.557 | 3.717 |
|  |  |  | Day 9 | -6.387^*^ | .969 | <.001 | -9.024 | -3.749 |
|  |  | Day 9 | Day 0 | 15.057^*^ | .969 | <.001 | 12.419 | 17.694 |
|  |  |  | Day 3 | 7.467^*^ | .969 | <.001 | 4.829 | 10.104 |
|  |  |  | Day 6 | 6.387^*^ | .969 | <.001 | 3.749 | 9.024 |
|  | 100 | Day 0 | Day 3 | -5.397^*^ | .969 | <.001 | -8.034 | -2.759 |
|  |  |  | Day 6 | -7.737^*^ | .969 | <.001 | -10.374 | -5.099 |
|  |  |  | Day 9 | -11.870^*^ | .969 | <.001 | -14.507 | -9.233 |
|  |  | Day 3 | Day 0 | 5.397^*^ | .969 | <.001 | 2.759 | 8.034 |
|  |  |  | Day 6 | -2.340 | .969 | .111 | -4.977 | .297 |
|  |  |  | Day 9 | -6.473^*^ | .969 | <.001 | -9.111 | -3.836 |
|  |  | Day 6 | Day 0 | 7.737^*^ | .969 | <.001 | 5.099 | 10.374 |
|  |  |  | Day 3 | 2.340 | .969 | .111 | -.297 | 4.977 |
|  |  |  | Day 9 | -4.133^*^ | .969 | <.001 | -6.771 | -1.496 |
|  |  | Day 9 | Day 0 | 11.870^*^ | .969 | <.001 | 9.233 | 14.507 |
|  |  |  | Day 3 | 6.473^*^ | .969 | <.001 | 3.836 | 9.111 |
|  |  |  | Day 6 | 4.133^*^ | .969 | <.001 | 1.496 | 6.771 |
|  | 200 | Day 0 | Day 3 | -4.380^*^ | .969 | <.001 | -7.017 | -1.743 |
|  |  |  | Day 6 | -7.900^*^ | .969 | <.001 | -10.537 | -5.263 |
|  |  |  | Day 9 | -10.493^*^ | .969 | <.001 | -13.131 | -7.856 |
|  |  | Day 3 | Day 0 | 4.380^*^ | .969 | <.001 | 1.743 | 7.017 |
|  |  |  | Day 6 | -3.520^*^ | .969 | .003 | -6.157 | -.883 |
|  |  |  | Day 9 | -6.113^*^ | .969 | <.001 | -8.751 | -3.476 |
|  |  | Day 6 | Day 0 | 7.900^*^ | .969 | <.001 | 5.263 | 10.537 |
|  |  |  | Day 3 | 3.520^*^ | .969 | .003 | .883 | 6.157 |
|  |  |  | Day 9 | -2.593 | .969 | .057 | -5.231 | .044 |
|  |  | Day 9 | Day 0 | 10.493^*^ | .969 | <.001 | 7.856 | 13.131 |
|  |  |  | Day 3 | 6.113^*^ | .969 | <.001 | 3.476 | 8.751 |
|  |  |  | Day 6 | 2.593 | .969 | .057 | -.044 | 5.231 |
|  | 300 | Day 0 | Day 3 | -3.587^*^ | .969 | .003 | -6.224 | -.949 |
|  |  |  | Day 6 | -6.593^*^ | .969 | <.001 | -9.231 | -3.956 |
|  |  |  | Day 9 | -13.523^*^ | .969 | <.001 | -16.161 | -10.886 |
|  |  | Day 3 | Day 0 | 3.587^*^ | .969 | .003 | .949 | 6.224 |
|  |  |  | Day 6 | -3.007^*^ | .969 | .017 | -5.644 | -.369 |
|  |  |  | Day 9 | -9.937^*^ | .969 | <.001 | -12.574 | -7.299 |
|  |  | Day 6 | Day 0 | 6.593^*^ | .969 | <.001 | 3.956 | 9.231 |
|  |  |  | Day 3 | 3.007^*^ | .969 | .017 | .369 | 5.644 |
|  |  |  | Day 9 | -6.930^*^ | .969 | <.001 | -9.567 | -4.293 |
|  |  | Day 9 | Day 0 | 13.523^*^ | .969 | <.001 | 10.886 | 16.161 |
|  |  |  | Day 3 | 9.937^*^ | .969 | <.001 | 7.299 | 12.574 |
|  |  |  | Day 6 | 6.930^*^ | .969 | <.001 | 4.293 | 9.567 |
| Based on estimated marginal means | | | | | | | | |
| *. The mean difference is significant at the .05 level. | | | | | | | | |
| b. Adjustment for multiple comparisons: Bonferroni. | | | | | | | | |

| Univariate Tests | | | | | | | | |
| --- | --- | --- | --- | --- | --- | --- | --- | --- |
| Dependent Variable: Electrolyte leakage | | | | | | | | |
| Light | SNP (µM) | | Sum of Squares | df | Mean Square | F | Sig. | Partial Eta Squared |
| White light | 0 | Contrast | 525.504 | 3 | 175.168 | 124.452 | <.001 | .854 |
|  |  | Error | 90.081 | 64 | 1.408 |  |  |  |
|  | 100 | Contrast | 377.357 | 3 | 125.786 | 89.367 | <.001 | .807 |
|  |  | Error | 90.081 | 64 | 1.408 |  |  |  |
|  | 200 | Contrast | 225.343 | 3 | 75.114 | 53.367 | <.001 | .714 |
|  |  | Error | 90.081 | 64 | 1.408 |  |  |  |
|  | 300 | Contrast | 447.416 | 3 | 149.139 | 105.959 | <.001 | .832 |
|  |  | Error | 90.081 | 64 | 1.408 |  |  |  |
| High blue light | 0 | Contrast | 342.890 | 3 | 114.297 | 81.205 | <.001 | .792 |
|  |  | Error | 90.081 | 64 | 1.408 |  |  |  |
|  | 100 | Contrast | 220.756 | 3 | 73.585 | 52.280 | <.001 | .710 |
|  |  | Error | 90.081 | 64 | 1.408 |  |  |  |
|  | 200 | Contrast | 186.145 | 3 | 62.048 | 44.083 | <.001 | .674 |
|  |  | Error | 90.081 | 64 | 1.408 |  |  |  |
|  | 300 | Contrast | 296.264 | 3 | 98.755 | 70.162 | <.001 | .767 |
|  |  | Error | 90.081 | 64 | 1.408 |  |  |  |
| Each F tests the simple effects of Day within each level combination of the other effects shown. These tests are based on the linearly independent pairwise comparisons among the estimated marginal means. | | | | | | | | |

**Post Hoc Tests**

| Electrolyte leakage | | | | |
| --- | --- | --- | --- | --- |
| Duncan^a,b^ | | | | |
| SNP (µM) | N | Subset | | |
|  |  | 1 | 2 | 3 |
| 200 | 24 | 22.6471 |  |  |
| 300 | 24 |  | 23.9325 |  |
| 100 | 24 |  | 24.1300 |  |
| 0 | 24 |  |  | 26.2704 |
| Sig. |  | 1.000 | .566 | 1.000 |
| Means for groups in homogeneous subsets are displayed.  Based on observed means.  The error term is Mean Square(Error) = 1.408. | | | | |
| a. Uses Harmonic Mean Sample Size = 24.000. | | | | |
| b. Alpha = .05. | | | | |

| Electrolyte leakage | | | | | |
| --- | --- | --- | --- | --- | --- |
| Duncan^a,b^ | | | | | |
| Day | N | Subset | | | |
|  |  | 1 | 2 | 3 | 4 |
| Day 0 | 24 | 17.2067 |  |  |  |
| Day 3 | 24 |  | 22.7846 |  |  |
| Day 6 | 24 |  |  | 25.6900 |  |
| Day 9 | 24 |  |  |  | 31.2988 |
| Sig. |  | 1.000 | 1.000 | 1.000 | 1.000 |
| Means for groups in homogeneous subsets are displayed.  Based on observed means.  The error term is Mean Square(Error) = 1.408. | | | | | |
| a. Uses Harmonic Mean Sample Size = 24.000. | | | | | |
| b. Alpha = .05. | | | | | |

**MDA content**

| Tests of Between-Subjects Effects | | | | | | |
| --- | --- | --- | --- | --- | --- | --- |
| Dependent Variable: MDA | | | | | | |
| Source | Type III Sum of Squares | df | Mean Square | F | Sig. | Partial Eta Squared |
| Corrected Model | 16552.982^a^ | 31 | 533.967 | 172.545 | <.001 | .988 |
| Intercept | 126266.963 | 1 | 126266.963 | 40801.698 | <.001 | .998 |
| Light | 22.790 | 1 | 22.790 | 7.364 | .009 | .103 |
| SNP | 2745.043 | 3 | 915.014 | 295.676 | <.001 | .933 |
| Day | 10760.149 | 3 | 3586.716 | 1159.006 | <.001 | .982 |
| Light * SNP | 1.668 | 3 | .556 | .180 | .910 | .008 |
| Light * Day | 65.543 | 3 | 21.848 | 7.060 | <.001 | .249 |
| SNP * Day | 2869.798 | 9 | 318.866 | 103.038 | <.001 | .935 |
| Light * SNP * Day | 87.991 | 9 | 9.777 | 3.159 | .003 | .308 |
| Error | 198.058 | 64 | 3.095 |  |  |  |
| Total | 143018.002 | 96 |  |  |  |  |
| Corrected Total | 16751.039 | 95 |  |  |  |  |
| a. R Squared = .988 (Adjusted R Squared = .982) | | | | | | |

**Light * SNP**

| Pairwise Comparisons | | | | | | | |
| --- | --- | --- | --- | --- | --- | --- | --- |
| Dependent Variable: MDA | | | | | | | |
| SNP (µM) | (I) Light | (J) Light | Mean Difference (I-J) | Std. Error | Sig.^a^ | 95% Confidence Interval for Difference^a^ | |
|  |  |  |  |  |  | Lower Bound | Upper Bound |
| 0 | White light | High blue light | 1.102 | .718 | .130 | -.333 | 2.537 |
|  | High blue light | White light | -1.102 | .718 | .130 | -2.537 | .333 |
| 100 | White light | High blue light | .753 | .718 | .299 | -.682 | 2.187 |
|  | High blue light | White light | -.753 | .718 | .299 | -2.187 | .682 |
| 200 | White light | High blue light | 1.344 | .718 | .066 | -.091 | 2.779 |
|  | High blue light | White light | -1.344 | .718 | .066 | -2.779 | .091 |
| 300 | White light | High blue light | .699 | .718 | .334 | -.736 | 2.134 |
|  | High blue light | White light | -.699 | .718 | .334 | -2.134 | .736 |
| Based on estimated marginal means | | | | | | | |
| a. Adjustment for multiple comparisons: Bonferroni. | | | | | | | |

| Univariate Tests | | | | | | | |
| --- | --- | --- | --- | --- | --- | --- | --- |
| Dependent Variable: MDA | | | | | | | |
| SNP (µM) | | Sum of Squares | df | Mean Square | F | Sig. | Partial Eta Squared |
| 0 | Contrast | 7.288 | 1 | 7.288 | 2.355 | .130 | .035 |
|  | Error | 198.058 | 64 | 3.095 |  |  |  |
| 100 | Contrast | 3.399 | 1 | 3.399 | 1.098 | .299 | .017 |
|  | Error | 198.058 | 64 | 3.095 |  |  |  |
| 200 | Contrast | 10.839 | 1 | 10.839 | 3.503 | .066 | .052 |
|  | Error | 198.058 | 64 | 3.095 |  |  |  |
| 300 | Contrast | 2.931 | 1 | 2.931 | .947 | .334 | .015 |
|  | Error | 198.058 | 64 | 3.095 |  |  |  |
| Each F tests the simple effects of Light within each level combination of the other effects shown. These tests are based on the linearly independent pairwise comparisons among the estimated marginal means. | | | | | | | |

| Pairwise Comparisons | | | | | | | |
| --- | --- | --- | --- | --- | --- | --- | --- |
| Dependent Variable: MDA | | | | | | | |
| Light | (I) SNP  (µM) | (J) SNP  (µM) | Mean Difference (I-J) | Std. Error | Sig.^b^ | 95% Confidence Interval for Difference^b^ | |
|  |  |  |  |  |  | Lower Bound | Upper Bound |
| White light | 0 | 100 | 9.516^*^ | .718 | <.001 | 7.561 | 11.472 |
|  |  | 200 | 11.505^*^ | .718 | <.001 | 9.550 | 13.461 |
|  |  | 300 | .027 | .718 | 1.000 | -1.929 | 1.982 |
|  | 100 | 0 | -9.516^*^ | .718 | <.001 | -11.472 | -7.561 |
|  |  | 200 | 1.989^*^ | .718 | .044 | .034 | 3.945 |
|  |  | 300 | -9.489^*^ | .718 | <.001 | -11.445 | -7.534 |
|  | 200 | 0 | -11.505^*^ | .718 | <.001 | -13.461 | -9.550 |
|  |  | 100 | -1.989^*^ | .718 | .044 | -3.945 | -.034 |
|  |  | 300 | -11.478^*^ | .718 | <.001 | -13.434 | -9.523 |
|  | 300 | 0 | -.027 | .718 | 1.000 | -1.982 | 1.929 |
|  |  | 100 | 9.489^*^ | .718 | <.001 | 7.534 | 11.445 |
|  |  | 200 | 11.478^*^ | .718 | <.001 | 9.523 | 13.434 |
| High blue light | 0 | 100 | 9.167^*^ | .718 | <.001 | 7.211 | 11.122 |
|  |  | 200 | 11.747^*^ | .718 | <.001 | 9.792 | 13.703 |
|  |  | 300 | -.376 | .718 | 1.000 | -2.332 | 1.579 |
|  | 100 | 0 | -9.167^*^ | .718 | <.001 | -11.122 | -7.211 |
|  |  | 200 | 2.581^*^ | .718 | .004 | .625 | 4.536 |
|  |  | 300 | -9.543^*^ | .718 | <.001 | -11.498 | -7.588 |
|  | 200 | 0 | -11.747^*^ | .718 | <.001 | -13.703 | -9.792 |
|  |  | 100 | -2.581^*^ | .718 | .004 | -4.536 | -.625 |
|  |  | 300 | -12.124^*^ | .718 | <.001 | -14.079 | -10.168 |
|  | 300 | 0 | .376 | .718 | 1.000 | -1.579 | 2.332 |
|  |  | 100 | 9.543^*^ | .718 | <.001 | 7.588 | 11.498 |
|  |  | 200 | 12.124^*^ | .718 | <.001 | 10.168 | 14.079 |
| Based on estimated marginal means | | | | | | | |
| *. The mean difference is significant at the .05 level. | | | | | | | |
| b. Adjustment for multiple comparisons: Bonferroni. | | | | | | | |

| Univariate Tests | | | | | | | |
| --- | --- | --- | --- | --- | --- | --- | --- |
| Dependent Variable: MDA | | | | | | | |
| Light | | Sum of Squares | df | Mean Square | F | Sig. | Partial Eta Squared |
| White light | Contrast | 1346.070 | 3 | 448.690 | 144.989 | <.001 | .872 |
|  | Error | 198.058 | 64 | 3.095 |  |  |  |
| High blue light | Contrast | 1400.642 | 3 | 466.881 | 150.867 | <.001 | .876 |
|  | Error | 198.058 | 64 | 3.095 |  |  |  |
| Each F tests the simple effects of SNP within each level combination of the other effects shown. These tests are based on the linearly independent pairwise comparisons among the estimated marginal means. | | | | | | | |

**Light * Day**

| Pairwise Comparisons | | | | | | | |
| --- | --- | --- | --- | --- | --- | --- | --- |
| Dependent Variable: MDA | | | | | | | |
| Day | (I) Light | (J) Light | Mean Difference (I-J) | Std. Error | Sig.^b^ | 95% Confidence Interval for Difference^b^ | |
|  |  |  |  |  |  | Lower Bound | Upper Bound |
| Day 0 | White light | High blue light | -.323 | .718 | .655 | -1.757 | 1.112 |
|  | High blue light | White light | .323 | .718 | .655 | -1.112 | 1.757 |
| Day 3 | White light | High blue light | 3.495^*^ | .718 | <.001 | 2.060 | 4.929 |
|  | High blue light | White light | -3.495^*^ | .718 | <.001 | -4.929 | -2.060 |
| Day 6 | White light | High blue light | -.672 | .718 | .353 | -2.107 | .763 |
|  | High blue light | White light | .672 | .718 | .353 | -.763 | 2.107 |
| Day 9 | White light | High blue light | 1.398 | .718 | .056 | -.037 | 2.833 |
|  | High blue light | White light | -1.398 | .718 | .056 | -2.833 | .037 |
| Based on estimated marginal means | | | | | | | |
| *. The mean difference is significant at the .05 level. | | | | | | | |
| b. Adjustment for multiple comparisons: Bonferroni. | | | | | | | |

| Univariate Tests | | | | | | | |
| --- | --- | --- | --- | --- | --- | --- | --- |
| Dependent Variable: MDA | | | | | | | |
| Day | | Sum of Squares | df | Mean Square | F | Sig. | Partial Eta Squared |
| Day 0 | Contrast | .624 | 1 | .624 | .202 | .655 | .003 |
|  | Error | 198.058 | 64 | 3.095 |  |  |  |
| Day 3 | Contrast | 73.274 | 1 | 73.274 | 23.678 | <.001 | .270 |
|  | Error | 198.058 | 64 | 3.095 |  |  |  |
| Day 6 | Contrast | 2.710 | 1 | 2.710 | .876 | .353 | .013 |
|  | Error | 198.058 | 64 | 3.095 |  |  |  |
| Day 9 | Contrast | 11.724 | 1 | 11.724 | 3.788 | .056 | .056 |
|  | Error | 198.058 | 64 | 3.095 |  |  |  |
| Each F tests the simple effects of Light within each level combination of the other effects shown. These tests are based on the linearly independent pairwise comparisons among the estimated marginal means. | | | | | | | |

**Light * Day**

| Pairwise Comparisons | | | | | | | |
| --- | --- | --- | --- | --- | --- | --- | --- |
| Dependent Variable: MDA | | | | | | | |
| Light | (I) Day | (J) Day | Mean Difference (I-J) | Std. Error | Sig.^b^ | 95% Confidence Interval for Difference^b^ | |
|  |  |  |  |  |  | Lower Bound | Upper Bound |
| White light | Day 0 | Day 3 | -12.930^*^ | .718 | <.001 | -14.886 | -10.975 |
|  |  | Day 6 | -24.758^*^ | .718 | <.001 | -26.713 | -22.803 |
|  |  | Day 9 | -26.317^*^ | .718 | <.001 | -28.273 | -24.362 |
|  | Day 3 | Day 0 | 12.930^*^ | .718 | <.001 | 10.975 | 14.886 |
|  |  | Day 6 | -11.828^*^ | .718 | <.001 | -13.783 | -9.873 |
|  |  | Day 9 | -13.387^*^ | .718 | <.001 | -15.343 | -11.432 |
|  | Day 6 | Day 0 | 24.758^*^ | .718 | <.001 | 22.803 | 26.713 |
|  |  | Day 3 | 11.828^*^ | .718 | <.001 | 9.873 | 13.783 |
|  |  | Day 9 | -1.559 | .718 | .202 | -3.515 | .396 |
|  | Day 9 | Day 0 | 26.317^*^ | .718 | <.001 | 24.362 | 28.273 |
|  |  | Day 3 | 13.387^*^ | .718 | <.001 | 11.432 | 15.343 |
|  |  | Day 6 | 1.559 | .718 | .202 | -.396 | 3.515 |
| High blue light | Day 0 | Day 3 | -9.113^*^ | .718 | <.001 | -11.068 | -7.157 |
|  |  | Day 6 | -25.108^*^ | .718 | <.001 | -27.063 | -23.152 |
|  |  | Day 9 | -24.597^*^ | .718 | <.001 | -26.552 | -22.641 |
|  | Day 3 | Day 0 | 9.113^*^ | .718 | <.001 | 7.157 | 11.068 |
|  |  | Day 6 | -15.995^*^ | .718 | <.001 | -17.950 | -14.039 |
|  |  | Day 9 | -15.484^*^ | .718 | <.001 | -17.439 | -13.528 |
|  | Day 6 | Day 0 | 25.108^*^ | .718 | <.001 | 23.152 | 27.063 |
|  |  | Day 3 | 15.995^*^ | .718 | <.001 | 14.039 | 17.950 |
|  |  | Day 9 | .511 | .718 | 1.000 | -1.445 | 2.466 |
|  | Day 9 | Day 0 | 24.597^*^ | .718 | <.001 | 22.641 | 26.552 |
|  |  | Day 3 | 15.484^*^ | .718 | <.001 | 13.528 | 17.439 |
|  |  | Day 6 | -.511 | .718 | 1.000 | -2.466 | 1.445 |
| Based on estimated marginal means | | | | | | | |
| *. The mean difference is significant at the .05 level. | | | | | | | |
| b. Adjustment for multiple comparisons: Bonferroni. | | | | | | | |

| Univariate Tests | | | | | | | |
| --- | --- | --- | --- | --- | --- | --- | --- |
| Dependent Variable: MDA | | | | | | | |
| Light | | Sum of Squares | df | Mean Square | F | Sig. | Partial Eta Squared |
| White light | Contrast | 5382.872 | 3 | 1794.291 | 579.804 | <.001 | .965 |
|  | Error | 198.058 | 64 | 3.095 |  |  |  |
| High blue light | Contrast | 5442.820 | 3 | 1814.273 | 586.261 | <.001 | .965 |
|  | Error | 198.058 | 64 | 3.095 |  |  |  |
| Each F tests the simple effects of Day within each level combination of the other effects shown. These tests are based on the linearly independent pairwise comparisons among the estimated marginal means. | | | | | | | |

**SNP * Day**

| Pairwise Comparisons | | | | | | | |
| --- | --- | --- | --- | --- | --- | --- | --- |
| Dependent Variable: MDA | | | | | | | |
| Day | (I) SNP  (µM) | (J) SNP  (µM) | Mean Difference (I-J) | Std. Error | Sig.^b^ | 95% Confidence Interval for Difference^b^ | |
|  |  |  |  |  |  | Lower Bound | Upper Bound |
| Day 0 | 0 | 100 | -7.105E-15 | 1.016 | 1.000 | -2.765 | 2.765 |
|  |  | 200 | -7.105E-15 | 1.016 | 1.000 | -2.765 | 2.765 |
|  |  | 300 | -3.331E-15 | 1.016 | 1.000 | -2.765 | 2.765 |
|  | 100 | 0 | 7.105E-15 | 1.016 | 1.000 | -2.765 | 2.765 |
|  |  | 200 | 3.553E-15 | 1.016 | 1.000 | -2.765 | 2.765 |
|  |  | 300 | 3.553E-15 | 1.016 | 1.000 | -2.765 | 2.765 |
|  | 200 | 0 | 7.105E-15 | 1.016 | 1.000 | -2.765 | 2.765 |
|  |  | 100 | -3.553E-15 | 1.016 | 1.000 | -2.765 | 2.765 |
|  |  | 300 | 3.553E-15 | 1.016 | 1.000 | -2.765 | 2.765 |
|  | 300 | 0 | 3.331E-15 | 1.016 | 1.000 | -2.765 | 2.765 |
|  |  | 100 | -3.553E-15 | 1.016 | 1.000 | -2.765 | 2.765 |
|  |  | 200 | -3.553E-15 | 1.016 | 1.000 | -2.765 | 2.765 |
| Day 3 | 0 | 100 | -2.957^*^ | 1.016 | .030 | -5.722 | -.192 |
|  |  | 200 | -2.849^*^ | 1.016 | .040 | -5.615 | -.084 |
|  |  | 300 | -7.097^*^ | 1.016 | <.001 | -9.862 | -4.331 |
|  | 100 | 0 | 2.957^*^ | 1.016 | .030 | .192 | 5.722 |
|  |  | 200 | .108 | 1.016 | 1.000 | -2.658 | 2.873 |
|  |  | 300 | -4.140^*^ | 1.016 | <.001 | -6.905 | -1.374 |
|  | 200 | 0 | 2.849^*^ | 1.016 | .040 | .084 | 5.615 |
|  |  | 100 | -.108 | 1.016 | 1.000 | -2.873 | 2.658 |
|  |  | 300 | -4.247^*^ | 1.016 | <.001 | -7.013 | -1.482 |
|  | 300 | 0 | 7.097^*^ | 1.016 | <.001 | 4.331 | 9.862 |
|  |  | 100 | 4.140^*^ | 1.016 | <.001 | 1.374 | 6.905 |
|  |  | 200 | 4.247^*^ | 1.016 | <.001 | 1.482 | 7.013 |
| Day 6 | 0 | 100 | 22.312^*^ | 1.016 | <.001 | 19.546 | 25.077 |
|  |  | 200 | 24.785^*^ | 1.016 | <.001 | 22.020 | 27.550 |
|  |  | 300 | 6.075^*^ | 1.016 | <.001 | 3.310 | 8.841 |
|  | 100 | 0 | -22.312^*^ | 1.016 | <.001 | -25.077 | -19.546 |
|  |  | 200 | 2.473 | 1.016 | .106 | -.292 | 5.238 |
|  |  | 300 | -16.237^*^ | 1.016 | <.001 | -19.002 | -13.471 |
|  | 200 | 0 | -24.785^*^ | 1.016 | <.001 | -27.550 | -22.020 |
|  |  | 100 | -2.473 | 1.016 | .106 | -5.238 | .292 |
|  |  | 300 | -18.710^*^ | 1.016 | <.001 | -21.475 | -15.944 |
|  | 300 | 0 | -6.075^*^ | 1.016 | <.001 | -8.841 | -3.310 |
|  |  | 100 | 16.237^*^ | 1.016 | <.001 | 13.471 | 19.002 |
|  |  | 200 | 18.710^*^ | 1.016 | <.001 | 15.944 | 21.475 |
| Day 9 | 0 | 100 | 18.011^*^ | 1.016 | <.001 | 15.245 | 20.776 |
|  |  | 200 | 24.570^*^ | 1.016 | <.001 | 21.805 | 27.335 |
|  |  | 300 | .323 | 1.016 | 1.000 | -2.443 | 3.088 |
|  | 100 | 0 | -18.011^*^ | 1.016 | <.001 | -20.776 | -15.245 |
|  |  | 200 | 6.559^*^ | 1.016 | <.001 | 3.794 | 9.324 |
|  |  | 300 | -17.688^*^ | 1.016 | <.001 | -20.454 | -14.923 |
|  | 200 | 0 | -24.570^*^ | 1.016 | <.001 | -27.335 | -21.805 |
|  |  | 100 | -6.559^*^ | 1.016 | <.001 | -9.324 | -3.794 |
|  |  | 300 | -24.247^*^ | 1.016 | <.001 | -27.013 | -21.482 |
|  | 300 | 0 | -.323 | 1.016 | 1.000 | -3.088 | 2.443 |
|  |  | 100 | 17.688^*^ | 1.016 | <.001 | 14.923 | 20.454 |
|  |  | 200 | 24.247^*^ | 1.016 | <.001 | 21.482 | 27.013 |
| Based on estimated marginal means | | | | | | | |
| *. The mean difference is significant at the .05 level. | | | | | | | |
| b. Adjustment for multiple comparisons: Bonferroni. | | | | | | | |

| Univariate Tests | | | | | | | |
| --- | --- | --- | --- | --- | --- | --- | --- |
| Dependent Variable: MDA | | | | | | | |
| Day | | Sum of Squares | df | Mean Square | F | Sig. | Partial Eta Squared |
| Day 0 | Contrast | 2.108E-28 | 3 | 7.028E-29 | .000 | 1.000 | .000 |
|  | Error | 198.058 | 64 | 3.095 |  |  |  |
| Day 3 | Contrast | 153.625 | 3 | 51.208 | 16.547 | <.001 | .437 |
|  | Error | 198.058 | 64 | 3.095 |  |  |  |
| Day 6 | Contrast | 2653.221 | 3 | 884.407 | 285.786 | <.001 | .931 |
|  | Error | 198.058 | 64 | 3.095 |  |  |  |
| Day 9 | Contrast | 2807.995 | 3 | 935.998 | 302.457 | <.001 | .934 |
|  | Error | 198.058 | 64 | 3.095 |  |  |  |
| Each F tests the simple effects of SNP within each level combination of the other effects shown. These tests are based on the linearly independent pairwise comparisons among the estimated marginal means. | | | | | | | |

| Pairwise Comparisons | | | | | | | |
| --- | --- | --- | --- | --- | --- | --- | --- |
| Dependent Variable: MDA | | | | | | | |
| SNP (µM) | (I) Day | (J) Day | Mean Difference (I-J) | Std. Error | Sig.^b^ | 95% Confidence Interval for Difference^b^ | |
|  |  |  |  |  |  | Lower Bound | Upper Bound |
| 0 | Day 0 | Day 3 | -7.796^*^ | 1.016 | <.001 | -10.561 | -5.030 |
|  |  | Day 6 | -38.226^*^ | 1.016 | <.001 | -40.991 | -35.460 |
|  |  | Day 9 | -36.183^*^ | 1.016 | <.001 | -38.948 | -33.417 |
|  | Day 3 | Day 0 | 7.796^*^ | 1.016 | <.001 | 5.030 | 10.561 |
|  |  | Day 6 | -30.430^*^ | 1.016 | <.001 | -33.195 | -27.665 |
|  |  | Day 9 | -28.387^*^ | 1.016 | <.001 | -31.152 | -25.622 |
|  | Day 6 | Day 0 | 38.226^*^ | 1.016 | <.001 | 35.460 | 40.991 |
|  |  | Day 3 | 30.430^*^ | 1.016 | <.001 | 27.665 | 33.195 |
|  |  | Day 9 | 2.043 | 1.016 | .291 | -.722 | 4.808 |
|  | Day 9 | Day 0 | 36.183^*^ | 1.016 | <.001 | 33.417 | 38.948 |
|  |  | Day 3 | 28.387^*^ | 1.016 | <.001 | 25.622 | 31.152 |
|  |  | Day 6 | -2.043 | 1.016 | .291 | -4.808 | .722 |
| 100 | Day 0 | Day 3 | -10.753^*^ | 1.016 | <.001 | -13.518 | -7.987 |
|  |  | Day 6 | -15.914^*^ | 1.016 | <.001 | -18.679 | -13.149 |
|  |  | Day 9 | -18.172^*^ | 1.016 | <.001 | -20.937 | -15.407 |
|  | Day 3 | Day 0 | 10.753^*^ | 1.016 | <.001 | 7.987 | 13.518 |
|  |  | Day 6 | -5.161^*^ | 1.016 | <.001 | -7.927 | -2.396 |
|  |  | Day 9 | -7.419^*^ | 1.016 | <.001 | -10.185 | -4.654 |
|  | Day 6 | Day 0 | 15.914^*^ | 1.016 | <.001 | 13.149 | 18.679 |
|  |  | Day 3 | 5.161^*^ | 1.016 | <.001 | 2.396 | 7.927 |
|  |  | Day 9 | -2.258 | 1.016 | .178 | -5.023 | .507 |
|  | Day 9 | Day 0 | 18.172^*^ | 1.016 | <.001 | 15.407 | 20.937 |
|  |  | Day 3 | 7.419^*^ | 1.016 | <.001 | 4.654 | 10.185 |
|  |  | Day 6 | 2.258 | 1.016 | .178 | -.507 | 5.023 |
| 200 | Day 0 | Day 3 | -10.645^*^ | 1.016 | <.001 | -13.411 | -7.880 |
|  |  | Day 6 | -13.441^*^ | 1.016 | <.001 | -16.206 | -10.676 |
|  |  | Day 9 | -11.613^*^ | 1.016 | <.001 | -14.378 | -8.848 |
|  | Day 3 | Day 0 | 10.645^*^ | 1.016 | <.001 | 7.880 | 13.411 |
|  |  | Day 6 | -2.796^*^ | 1.016 | .046 | -5.561 | -.030 |
|  |  | Day 9 | -.968 | 1.016 | 1.000 | -3.733 | 1.798 |
|  | Day 6 | Day 0 | 13.441^*^ | 1.016 | <.001 | 10.676 | 16.206 |
|  |  | Day 3 | 2.796^*^ | 1.016 | .046 | .030 | 5.561 |
|  |  | Day 9 | 1.828 | 1.016 | .460 | -.937 | 4.593 |
|  | Day 9 | Day 0 | 11.613^*^ | 1.016 | <.001 | 8.848 | 14.378 |
|  |  | Day 3 | .968 | 1.016 | 1.000 | -1.798 | 3.733 |
|  |  | Day 6 | -1.828 | 1.016 | .460 | -4.593 | .937 |
| 300 | Day 0 | Day 3 | -14.892^*^ | 1.016 | <.001 | -17.658 | -12.127 |
|  |  | Day 6 | -32.151^*^ | 1.016 | <.001 | -34.916 | -29.385 |
|  |  | Day 9 | -35.860^*^ | 1.016 | <.001 | -38.626 | -33.095 |
|  | Day 3 | Day 0 | 14.892^*^ | 1.016 | <.001 | 12.127 | 17.658 |
|  |  | Day 6 | -17.258^*^ | 1.016 | <.001 | -20.023 | -14.493 |
|  |  | Day 9 | -20.968^*^ | 1.016 | <.001 | -23.733 | -18.202 |
|  | Day 6 | Day 0 | 32.151^*^ | 1.016 | <.001 | 29.385 | 34.916 |
|  |  | Day 3 | 17.258^*^ | 1.016 | <.001 | 14.493 | 20.023 |
|  |  | Day 9 | -3.710^*^ | 1.016 | .003 | -6.475 | -.944 |
|  | Day 9 | Day 0 | 35.860^*^ | 1.016 | <.001 | 33.095 | 38.626 |
|  |  | Day 3 | 20.968^*^ | 1.016 | <.001 | 18.202 | 23.733 |
|  |  | Day 6 | 3.710^*^ | 1.016 | .003 | .944 | 6.475 |
| Based on estimated marginal means | | | | | | | |
| *. The mean difference is significant at the .05 level. | | | | | | | |
| b. Adjustment for multiple comparisons: Bonferroni. | | | | | | | |

| Univariate Tests | | | | | | | |
| --- | --- | --- | --- | --- | --- | --- | --- |
| Dependent Variable: MDA | | | | | | | |
| SNP (µM) | | Sum of Squares | df | Mean Square | F | Sig. | Partial Eta Squared |
| 0 | Contrast | 6850.759 | 3 | 2283.586 | 737.914 | <.001 | .972 |
|  | Error | 198.058 | 64 | 3.095 |  |  |  |
| 100 | Contrast | 1178.824 | 3 | 392.941 | 126.974 | <.001 | .856 |
|  | Error | 198.058 | 64 | 3.095 |  |  |  |
| 200 | Contrast | 661.394 | 3 | 220.465 | 71.241 | <.001 | .770 |
|  | Error | 198.058 | 64 | 3.095 |  |  |  |
| 300 | Contrast | 4938.970 | 3 | 1646.323 | 531.990 | <.001 | .961 |
|  | Error | 198.058 | 64 | 3.095 |  |  |  |
| Each F tests the simple effects of Day within each level combination of the other effects shown. These tests are based on the linearly independent pairwise comparisons among the estimated marginal means. | | | | | | | |

**Light * SNP * Day**

| Pairwise Comparisons | | | | | | | | |
| --- | --- | --- | --- | --- | --- | --- | --- | --- |
| Dependent Variable: MDA | | | | | | | | |
| SNP (µM) | Day | (I) Light | (J) Light | Mean Difference (I-J) | Std. Error | Sig.^b^ | 95% Confidence Interval for Difference^b^ | |
|  |  |  |  |  |  |  | Lower Bound | Upper Bound |
| 0 | Day 0 | White light | High blue light | -.323 | 1.436 | .823 | -3.192 | 2.547 |
|  |  | High blue light | White light | .323 | 1.436 | .823 | -2.547 | 3.192 |
|  | Day 3 | White light | High blue light | 1.290 | 1.436 | .372 | -1.579 | 4.160 |
|  |  | High blue light | White light | -1.290 | 1.436 | .372 | -4.160 | 1.579 |
|  | Day 6 | White light | High blue light | -1.505 | 1.436 | .299 | -4.375 | 1.364 |
|  |  | High blue light | White light | 1.505 | 1.436 | .299 | -1.364 | 4.375 |
|  | Day 9 | White light | High blue light | 4.946^*^ | 1.436 | .001 | 2.077 | 7.816 |
|  |  | High blue light | White light | -4.946^*^ | 1.436 | .001 | -7.816 | -2.077 |
| 100 | Day 0 | White light | High blue light | -.323 | 1.436 | .823 | -3.192 | 2.547 |
|  |  | High blue light | White light | .323 | 1.436 | .823 | -2.547 | 3.192 |
|  | Day 3 | White light | High blue light | 6.774^*^ | 1.436 | <.001 | 3.905 | 9.644 |
|  |  | High blue light | White light | -6.774^*^ | 1.436 | <.001 | -9.644 | -3.905 |
|  | Day 6 | White light | High blue light | -1.613 | 1.436 | .266 | -4.482 | 1.257 |
|  |  | High blue light | White light | 1.613 | 1.436 | .266 | -1.257 | 4.482 |
|  | Day 9 | White light | High blue light | -1.828 | 1.436 | .208 | -4.697 | 1.041 |
|  |  | High blue light | White light | 1.828 | 1.436 | .208 | -1.041 | 4.697 |
| 200 | Day 0 | White light | High blue light | -.323 | 1.436 | .823 | -3.192 | 2.547 |
|  |  | High blue light | White light | .323 | 1.436 | .823 | -2.547 | 3.192 |
|  | Day 3 | White light | High blue light | 5.699^*^ | 1.436 | <.001 | 2.829 | 8.568 |
|  |  | High blue light | White light | -5.699^*^ | 1.436 | <.001 | -8.568 | -2.829 |
|  | Day 6 | White light | High blue light | -.753 | 1.436 | .602 | -3.622 | 2.117 |
|  |  | High blue light | White light | .753 | 1.436 | .602 | -2.117 | 3.622 |
|  | Day 9 | White light | High blue light | .753 | 1.436 | .602 | -2.117 | 3.622 |
|  |  | High blue light | White light | -.753 | 1.436 | .602 | -3.622 | 2.117 |
| 300 | Day 0 | White light | High blue light | -.323 | 1.436 | .823 | -3.192 | 2.547 |
|  |  | High blue light | White light | .323 | 1.436 | .823 | -2.547 | 3.192 |
|  | Day 3 | White light | High blue light | .215 | 1.436 | .881 | -2.654 | 3.084 |
|  |  | High blue light | White light | -.215 | 1.436 | .881 | -3.084 | 2.654 |
|  | Day 6 | White light | High blue light | 1.183 | 1.436 | .413 | -1.687 | 4.052 |
|  |  | High blue light | White light | -1.183 | 1.436 | .413 | -4.052 | 1.687 |
|  | Day 9 | White light | High blue light | 1.720 | 1.436 | .235 | -1.149 | 4.590 |
|  |  | High blue light | White light | -1.720 | 1.436 | .235 | -4.590 | 1.149 |
| Based on estimated marginal means | | | | | | | | |
| *. The mean difference is significant at the .05 level. | | | | | | | | |
| b. Adjustment for multiple comparisons: Bonferroni. | | | | | | | | |

| Univariate Tests | | | | | | | | |
| --- | --- | --- | --- | --- | --- | --- | --- | --- |
| Dependent Variable: MDA | | | | | | | | |
| SNP (µM) | Day | | Sum of Squares | df | Mean Square | F | Sig. | Partial Eta Squared |
| 0 | Day 0 | Contrast | .156 | 1 | .156 | .050 | .823 | .001 |
|  |  | Error | 198.058 | 64 | 3.095 |  |  |  |
|  | Day 3 | Contrast | 2.497 | 1 | 2.497 | .807 | .372 | .012 |
|  |  | Error | 198.058 | 64 | 3.095 |  |  |  |
|  | Day 6 | Contrast | 3.399 | 1 | 3.399 | 1.098 | .299 | .017 |
|  |  | Error | 198.058 | 64 | 3.095 |  |  |  |
|  | Day 9 | Contrast | 36.698 | 1 | 36.698 | 11.858 | .001 | .156 |
|  |  | Error | 198.058 | 64 | 3.095 |  |  |  |
| 100 | Day 0 | Contrast | .156 | 1 | .156 | .050 | .823 | .001 |
|  |  | Error | 198.058 | 64 | 3.095 |  |  |  |
|  | Day 3 | Contrast | 68.835 | 1 | 68.835 | 22.243 | <.001 | .258 |
|  |  | Error | 198.058 | 64 | 3.095 |  |  |  |
|  | Day 6 | Contrast | 3.902 | 1 | 3.902 | 1.261 | .266 | .019 |
|  |  | Error | 198.058 | 64 | 3.095 |  |  |  |
|  | Day 9 | Contrast | 5.012 | 1 | 5.012 | 1.620 | .208 | .025 |
|  |  | Error | 198.058 | 64 | 3.095 |  |  |  |
| 200 | Day 0 | Contrast | .156 | 1 | .156 | .050 | .823 | .001 |
|  |  | Error | 198.058 | 64 | 3.095 |  |  |  |
|  | Day 3 | Contrast | 48.717 | 1 | 48.717 | 15.742 | <.001 | .197 |
|  |  | Error | 198.058 | 64 | 3.095 |  |  |  |
|  | Day 6 | Contrast | .850 | 1 | .850 | .275 | .602 | .004 |
|  |  | Error | 198.058 | 64 | 3.095 |  |  |  |
|  | Day 9 | Contrast | .850 | 1 | .850 | .275 | .602 | .004 |
|  |  | Error | 198.058 | 64 | 3.095 |  |  |  |
| 300 | Day 0 | Contrast | .156 | 1 | .156 | .050 | .823 | .001 |
|  |  | Error | 198.058 | 64 | 3.095 |  |  |  |
|  | Day 3 | Contrast | .069 | 1 | .069 | .022 | .881 | .000 |
|  |  | Error | 198.058 | 64 | 3.095 |  |  |  |
|  | Day 6 | Contrast | 2.099 | 1 | 2.099 | .678 | .413 | .010 |
|  |  | Error | 198.058 | 64 | 3.095 |  |  |  |
|  | Day 9 | Contrast | 4.440 | 1 | 4.440 | 1.435 | .235 | .022 |
|  |  | Error | 198.058 | 64 | 3.095 |  |  |  |
| Each F tests the simple effects of Light within each level combination of the other effects shown. These tests are based on the linearly independent pairwise comparisons among the estimated marginal means. | | | | | | | | |

| Pairwise Comparisons | | | | | | | | |
| --- | --- | --- | --- | --- | --- | --- | --- | --- |
| Dependent Variable: MDA | | | | | | | | |
| Light | Day | (I) SNP  (µM) | (J) SNP (µM) | Mean Difference (I-J) | Std. Error | Sig.^b^ | 95% Confidence Interval for Difference^b^ | |
|  |  |  |  |  |  |  | Lower Bound | Upper Bound |
| White light | Day 0 | 0 | 100 | -7.105E-15 | 1.436 | 1.000 | -3.911 | 3.911 |
|  |  |  | 200 | -1.066E-14 | 1.436 | 1.000 | -3.911 | 3.911 |
|  |  |  | 300 | -3.997E-15 | 1.436 | 1.000 | -3.911 | 3.911 |
|  |  | 100 | 0 | 7.105E-15 | 1.436 | 1.000 | -3.911 | 3.911 |
|  |  |  | 200 | .000 | 1.436 | 1.000 | -3.911 | 3.911 |
|  |  |  | 300 | 3.553E-15 | 1.436 | 1.000 | -3.911 | 3.911 |
|  |  | 200 | 0 | 1.066E-14 | 1.436 | 1.000 | -3.911 | 3.911 |
|  |  |  | 100 | .000 | 1.436 | 1.000 | -3.911 | 3.911 |
|  |  |  | 300 | 3.553E-15 | 1.436 | 1.000 | -3.911 | 3.911 |
|  |  | 300 | 0 | 3.997E-15 | 1.436 | 1.000 | -3.911 | 3.911 |
|  |  |  | 100 | -3.553E-15 | 1.436 | 1.000 | -3.911 | 3.911 |
|  |  |  | 200 | -3.553E-15 | 1.436 | 1.000 | -3.911 | 3.911 |
|  | Day 3 | 0 | 100 | -5.699^*^ | 1.436 | .001 | -9.610 | -1.788 |
|  |  |  | 200 | -5.054^*^ | 1.436 | .005 | -8.965 | -1.143 |
|  |  |  | 300 | -6.559^*^ | 1.436 | <.001 | -10.470 | -2.648 |
|  |  | 100 | 0 | 5.699^*^ | 1.436 | .001 | 1.788 | 9.610 |
|  |  |  | 200 | .645 | 1.436 | 1.000 | -3.266 | 4.556 |
|  |  |  | 300 | -.860 | 1.436 | 1.000 | -4.771 | 3.051 |
|  |  | 200 | 0 | 5.054^*^ | 1.436 | .005 | 1.143 | 8.965 |
|  |  |  | 100 | -.645 | 1.436 | 1.000 | -4.556 | 3.266 |
|  |  |  | 300 | -1.505 | 1.436 | 1.000 | -5.416 | 2.405 |
|  |  | 300 | 0 | 6.559^*^ | 1.436 | <.001 | 2.648 | 10.470 |
|  |  |  | 100 | .860 | 1.436 | 1.000 | -3.051 | 4.771 |
|  |  |  | 200 | 1.505 | 1.436 | 1.000 | -2.405 | 5.416 |
|  | Day 6 | 0 | 100 | 22.366^*^ | 1.436 | <.001 | 18.455 | 26.276 |
|  |  |  | 200 | 24.409^*^ | 1.436 | <.001 | 20.498 | 28.319 |
|  |  |  | 300 | 4.731^*^ | 1.436 | .010 | .820 | 8.642 |
|  |  | 100 | 0 | -22.366^*^ | 1.436 | <.001 | -26.276 | -18.455 |
|  |  |  | 200 | 2.043 | 1.436 | .959 | -1.868 | 5.954 |
|  |  |  | 300 | -17.634^*^ | 1.436 | <.001 | -21.545 | -13.724 |
|  |  | 200 | 0 | -24.409^*^ | 1.436 | <.001 | -28.319 | -20.498 |
|  |  |  | 100 | -2.043 | 1.436 | .959 | -5.954 | 1.868 |
|  |  |  | 300 | -19.677^*^ | 1.436 | <.001 | -23.588 | -15.767 |
|  |  | 300 | 0 | -4.731^*^ | 1.436 | .010 | -8.642 | -.820 |
|  |  |  | 100 | 17.634^*^ | 1.436 | <.001 | 13.724 | 21.545 |
|  |  |  | 200 | 19.677^*^ | 1.436 | <.001 | 15.767 | 23.588 |
|  | Day 9 | 0 | 100 | 21.398^*^ | 1.436 | <.001 | 17.487 | 25.309 |
|  |  |  | 200 | 26.667^*^ | 1.436 | <.001 | 22.756 | 30.577 |
|  |  |  | 300 | 1.935 | 1.436 | 1.000 | -1.975 | 5.846 |
|  |  | 100 | 0 | -21.398^*^ | 1.436 | <.001 | -25.309 | -17.487 |
|  |  |  | 200 | 5.269^*^ | 1.436 | .003 | 1.358 | 9.180 |
|  |  |  | 300 | -19.462^*^ | 1.436 | <.001 | -23.373 | -15.552 |
|  |  | 200 | 0 | -26.667^*^ | 1.436 | <.001 | -30.577 | -22.756 |
|  |  |  | 100 | -5.269^*^ | 1.436 | .003 | -9.180 | -1.358 |
|  |  |  | 300 | -24.731^*^ | 1.436 | <.001 | -28.642 | -20.820 |
|  |  | 300 | 0 | -1.935 | 1.436 | 1.000 | -5.846 | 1.975 |
|  |  |  | 100 | 19.462^*^ | 1.436 | <.001 | 15.552 | 23.373 |
|  |  |  | 200 | 24.731^*^ | 1.436 | <.001 | 20.820 | 28.642 |
| High blue light | Day 0 | 0 | 100 | -7.105E-15 | 1.436 | 1.000 | -3.911 | 3.911 |
|  |  |  | 200 | -7.105E-15 | 1.436 | 1.000 | -3.911 | 3.911 |
|  |  |  | 300 | -2.887E-15 | 1.436 | 1.000 | -3.911 | 3.911 |
|  |  | 100 | 0 | 7.105E-15 | 1.436 | 1.000 | -3.911 | 3.911 |
|  |  |  | 200 | -1.776E-15 | 1.436 | 1.000 | -3.911 | 3.911 |
|  |  |  | 300 | 3.553E-15 | 1.436 | 1.000 | -3.911 | 3.911 |
|  |  | 200 | 0 | 7.105E-15 | 1.436 | 1.000 | -3.911 | 3.911 |
|  |  |  | 100 | 1.776E-15 | 1.436 | 1.000 | -3.911 | 3.911 |
|  |  |  | 300 | 3.553E-15 | 1.436 | 1.000 | -3.911 | 3.911 |
|  |  | 300 | 0 | 2.887E-15 | 1.436 | 1.000 | -3.911 | 3.911 |
|  |  |  | 100 | -3.553E-15 | 1.436 | 1.000 | -3.911 | 3.911 |
|  |  |  | 200 | -3.553E-15 | 1.436 | 1.000 | -3.911 | 3.911 |
|  | Day 3 | 0 | 100 | -.215 | 1.436 | 1.000 | -4.126 | 3.696 |
|  |  |  | 200 | -.645 | 1.436 | 1.000 | -4.556 | 3.266 |
|  |  |  | 300 | -7.634^*^ | 1.436 | <.001 | -11.545 | -3.724 |
|  |  | 100 | 0 | .215 | 1.436 | 1.000 | -3.696 | 4.126 |
|  |  |  | 200 | -.430 | 1.436 | 1.000 | -4.341 | 3.481 |
|  |  |  | 300 | -7.419^*^ | 1.436 | <.001 | -11.330 | -3.509 |
|  |  | 200 | 0 | .645 | 1.436 | 1.000 | -3.266 | 4.556 |
|  |  |  | 100 | .430 | 1.436 | 1.000 | -3.481 | 4.341 |
|  |  |  | 300 | -6.989^*^ | 1.436 | <.001 | -10.900 | -3.078 |
|  |  | 300 | 0 | 7.634^*^ | 1.436 | <.001 | 3.724 | 11.545 |
|  |  |  | 100 | 7.419^*^ | 1.436 | <.001 | 3.509 | 11.330 |
|  |  |  | 200 | 6.989^*^ | 1.436 | <.001 | 3.078 | 10.900 |
|  | Day 6 | 0 | 100 | 22.258^*^ | 1.436 | <.001 | 18.347 | 26.169 |
|  |  |  | 200 | 25.161^*^ | 1.436 | <.001 | 21.250 | 29.072 |
|  |  |  | 300 | 7.419^*^ | 1.436 | <.001 | 3.509 | 11.330 |
|  |  | 100 | 0 | -22.258^*^ | 1.436 | <.001 | -26.169 | -18.347 |
|  |  |  | 200 | 2.903 | 1.436 | .285 | -1.008 | 6.814 |
|  |  |  | 300 | -14.839^*^ | 1.436 | <.001 | -18.750 | -10.928 |
|  |  | 200 | 0 | -25.161^*^ | 1.436 | <.001 | -29.072 | -21.250 |
|  |  |  | 100 | -2.903 | 1.436 | .285 | -6.814 | 1.008 |
|  |  |  | 300 | -17.742^*^ | 1.436 | <.001 | -21.653 | -13.831 |
|  |  | 300 | 0 | -7.419^*^ | 1.436 | <.001 | -11.330 | -3.509 |
|  |  |  | 100 | 14.839^*^ | 1.436 | <.001 | 10.928 | 18.750 |
|  |  |  | 200 | 17.742^*^ | 1.436 | <.001 | 13.831 | 21.653 |
|  | Day 9 | 0 | 100 | 14.624^*^ | 1.436 | <.001 | 10.713 | 18.534 |
|  |  |  | 200 | 22.473^*^ | 1.436 | <.001 | 18.562 | 26.384 |
|  |  |  | 300 | -1.290 | 1.436 | 1.000 | -5.201 | 2.620 |
|  |  | 100 | 0 | -14.624^*^ | 1.436 | <.001 | -18.534 | -10.713 |
|  |  |  | 200 | 7.849^*^ | 1.436 | <.001 | 3.939 | 11.760 |
|  |  |  | 300 | -15.914^*^ | 1.436 | <.001 | -19.825 | -12.003 |
|  |  | 200 | 0 | -22.473^*^ | 1.436 | <.001 | -26.384 | -18.562 |
|  |  |  | 100 | -7.849^*^ | 1.436 | <.001 | -11.760 | -3.939 |
|  |  |  | 300 | -23.763^*^ | 1.436 | <.001 | -27.674 | -19.853 |
|  |  | 300 | 0 | 1.290 | 1.436 | 1.000 | -2.620 | 5.201 |
|  |  |  | 100 | 15.914^*^ | 1.436 | <.001 | 12.003 | 19.825 |
|  |  |  | 200 | 23.763^*^ | 1.436 | <.001 | 19.853 | 27.674 |
| Based on estimated marginal means | | | | | | | | |
| *. The mean difference is significant at the .05 level. | | | | | | | | |
| b. Adjustment for multiple comparisons: Bonferroni. | | | | | | | | |

| Univariate Tests | | | | | | | | |
| --- | --- | --- | --- | --- | --- | --- | --- | --- |
| Dependent Variable: MDA | | | | | | | | |
| Light | Day | | Sum of Squares | df | Mean Square | F | Sig. | Partial Eta Squared |
| White light | Day 0 | Contrast | 1.850E-28 | 3 | 6.168E-29 | .000 | 1.000 | .000 |
|  |  | Error | 198.058 | 64 | 3.095 |  |  |  |
|  | Day 3 | Contrast | 78.347 | 3 | 26.116 | 8.439 | <.001 | .283 |
|  |  | Error | 198.058 | 64 | 3.095 |  |  |  |
|  | Day 6 | Contrast | 1365.548 | 3 | 455.183 | 147.087 | <.001 | .873 |
|  |  | Error | 198.058 | 64 | 3.095 |  |  |  |
|  | Day 9 | Contrast | 1643.176 | 3 | 547.725 | 176.991 | <.001 | .892 |
|  |  | Error | 198.058 | 64 | 3.095 |  |  |  |
| High blue light | Day 0 | Contrast | 1.087E-28 | 3 | 3.623E-29 | .000 | 1.000 | .000 |
|  |  | Error | 198.058 | 64 | 3.095 |  |  |  |
|  | Day 3 | Contrast | 122.121 | 3 | 40.707 | 13.154 | <.001 | .381 |
|  |  | Error | 198.058 | 64 | 3.095 |  |  |  |
|  | Day 6 | Contrast | 1295.213 | 3 | 431.738 | 139.511 | <.001 | .867 |
|  |  | Error | 198.058 | 64 | 3.095 |  |  |  |
|  | Day 9 | Contrast | 1200.095 | 3 | 400.032 | 129.266 | <.001 | .858 |
|  |  | Error | 198.058 | 64 | 3.095 |  |  |  |
| Each F tests the simple effects of SNP within each level combination of the other effects shown. These tests are based on the linearly independent pairwise comparisons among the estimated marginal means. | | | | | | | | |

| Pairwise Comparisons | | | | | | | | |
| --- | --- | --- | --- | --- | --- | --- | --- | --- |
| Dependent Variable: MDA | | | | | | | | |
| Light | SNP  (µM) | (I) Day | (J) Day | Mean Difference (I-J) | Std. Error | Sig.^b^ | 95% Confidence Interval for Difference^b^ | |
|  |  |  |  |  |  |  | Lower Bound | Upper Bound |
| White light | 0 | Day 0 | Day 3 | -8.602^*^ | 1.436 | <.001 | -12.513 | -4.691 |
|  |  |  | Day 6 | -37.634^*^ | 1.436 | <.001 | -41.545 | -33.724 |
|  |  |  | Day 9 | -38.817^*^ | 1.436 | <.001 | -42.728 | -34.906 |
|  |  | Day 3 | Day 0 | 8.602^*^ | 1.436 | <.001 | 4.691 | 12.513 |
|  |  |  | Day 6 | -29.032^*^ | 1.436 | <.001 | -32.943 | -25.121 |
|  |  |  | Day 9 | -30.215^*^ | 1.436 | <.001 | -34.126 | -26.304 |
|  |  | Day 6 | Day 0 | 37.634^*^ | 1.436 | <.001 | 33.724 | 41.545 |
|  |  |  | Day 3 | 29.032^*^ | 1.436 | <.001 | 25.121 | 32.943 |
|  |  |  | Day 9 | -1.183 | 1.436 | 1.000 | -5.094 | 2.728 |
|  |  | Day 9 | Day 0 | 38.817^*^ | 1.436 | <.001 | 34.906 | 42.728 |
|  |  |  | Day 3 | 30.215^*^ | 1.436 | <.001 | 26.304 | 34.126 |
|  |  |  | Day 6 | 1.183 | 1.436 | 1.000 | -2.728 | 5.094 |
|  | 100 | Day 0 | Day 3 | -14.301^*^ | 1.436 | <.001 | -18.212 | -10.390 |
|  |  |  | Day 6 | -15.269^*^ | 1.436 | <.001 | -19.180 | -11.358 |
|  |  |  | Day 9 | -17.419^*^ | 1.436 | <.001 | -21.330 | -13.509 |
|  |  | Day 3 | Day 0 | 14.301^*^ | 1.436 | <.001 | 10.390 | 18.212 |
|  |  |  | Day 6 | -.968 | 1.436 | 1.000 | -4.879 | 2.943 |
|  |  |  | Day 9 | -3.118 | 1.436 | .202 | -7.029 | .793 |
|  |  | Day 6 | Day 0 | 15.269^*^ | 1.436 | <.001 | 11.358 | 19.180 |
|  |  |  | Day 3 | .968 | 1.436 | 1.000 | -2.943 | 4.879 |
|  |  |  | Day 9 | -2.151 | 1.436 | .836 | -6.061 | 1.760 |
|  |  | Day 9 | Day 0 | 17.419^*^ | 1.436 | <.001 | 13.509 | 21.330 |
|  |  |  | Day 3 | 3.118 | 1.436 | .202 | -.793 | 7.029 |
|  |  |  | Day 6 | 2.151 | 1.436 | .836 | -1.760 | 6.061 |
|  | 200 | Day 0 | Day 3 | -13.656^*^ | 1.436 | <.001 | -17.567 | -9.745 |
|  |  |  | Day 6 | -13.226^*^ | 1.436 | <.001 | -17.137 | -9.315 |
|  |  |  | Day 9 | -12.151^*^ | 1.436 | <.001 | -16.061 | -8.240 |
|  |  | Day 3 | Day 0 | 13.656^*^ | 1.436 | <.001 | 9.745 | 17.567 |
|  |  |  | Day 6 | .430 | 1.436 | 1.000 | -3.481 | 4.341 |
|  |  |  | Day 9 | 1.505 | 1.436 | 1.000 | -2.405 | 5.416 |
|  |  | Day 6 | Day 0 | 13.226^*^ | 1.436 | <.001 | 9.315 | 17.137 |
|  |  |  | Day 3 | -.430 | 1.436 | 1.000 | -4.341 | 3.481 |
|  |  |  | Day 9 | 1.075 | 1.436 | 1.000 | -2.836 | 4.986 |
|  |  | Day 9 | Day 0 | 12.151^*^ | 1.436 | <.001 | 8.240 | 16.061 |
|  |  |  | Day 3 | -1.505 | 1.436 | 1.000 | -5.416 | 2.405 |
|  |  |  | Day 6 | -1.075 | 1.436 | 1.000 | -4.986 | 2.836 |
|  | 300 | Day 0 | Day 3 | -15.161^*^ | 1.436 | <.001 | -19.072 | -11.250 |
|  |  |  | Day 6 | -32.903^*^ | 1.436 | <.001 | -36.814 | -28.992 |
|  |  |  | Day 9 | -36.882^*^ | 1.436 | <.001 | -40.793 | -32.971 |
|  |  | Day 3 | Day 0 | 15.161^*^ | 1.436 | <.001 | 11.250 | 19.072 |
|  |  |  | Day 6 | -17.742^*^ | 1.436 | <.001 | -21.653 | -13.831 |
|  |  |  | Day 9 | -21.720^*^ | 1.436 | <.001 | -25.631 | -17.810 |
|  |  | Day 6 | Day 0 | 32.903^*^ | 1.436 | <.001 | 28.992 | 36.814 |
|  |  |  | Day 3 | 17.742^*^ | 1.436 | <.001 | 13.831 | 21.653 |
|  |  |  | Day 9 | -3.978^*^ | 1.436 | .044 | -7.889 | -.068 |
|  |  | Day 9 | Day 0 | 36.882^*^ | 1.436 | <.001 | 32.971 | 40.793 |
|  |  |  | Day 3 | 21.720^*^ | 1.436 | <.001 | 17.810 | 25.631 |
|  |  |  | Day 6 | 3.978^*^ | 1.436 | .044 | .068 | 7.889 |
| High blue light | 0 | Day 0 | Day 3 | -6.989^*^ | 1.436 | <.001 | -10.900 | -3.078 |
|  |  |  | Day 6 | -38.817^*^ | 1.436 | <.001 | -42.728 | -34.906 |
|  |  |  | Day 9 | -33.548^*^ | 1.436 | <.001 | -37.459 | -29.638 |
|  |  | Day 3 | Day 0 | 6.989^*^ | 1.436 | <.001 | 3.078 | 10.900 |
|  |  |  | Day 6 | -31.828^*^ | 1.436 | <.001 | -35.739 | -27.917 |
|  |  |  | Day 9 | -26.559^*^ | 1.436 | <.001 | -30.470 | -22.648 |
|  |  | Day 6 | Day 0 | 38.817^*^ | 1.436 | <.001 | 34.906 | 42.728 |
|  |  |  | Day 3 | 31.828^*^ | 1.436 | <.001 | 27.917 | 35.739 |
|  |  |  | Day 9 | 5.269^*^ | 1.436 | .003 | 1.358 | 9.180 |
|  |  | Day 9 | Day 0 | 33.548^*^ | 1.436 | <.001 | 29.638 | 37.459 |
|  |  |  | Day 3 | 26.559^*^ | 1.436 | <.001 | 22.648 | 30.470 |
|  |  |  | Day 6 | -5.269^*^ | 1.436 | .003 | -9.180 | -1.358 |
|  | 100 | Day 0 | Day 3 | -7.204^*^ | 1.436 | <.001 | -11.115 | -3.293 |
|  |  |  | Day 6 | -16.559^*^ | 1.436 | <.001 | -20.470 | -12.648 |
|  |  |  | Day 9 | -18.925^*^ | 1.436 | <.001 | -22.836 | -15.014 |
|  |  | Day 3 | Day 0 | 7.204^*^ | 1.436 | <.001 | 3.293 | 11.115 |
|  |  |  | Day 6 | -9.355^*^ | 1.436 | <.001 | -13.266 | -5.444 |
|  |  |  | Day 9 | -11.720^*^ | 1.436 | <.001 | -15.631 | -7.810 |
|  |  | Day 6 | Day 0 | 16.559^*^ | 1.436 | <.001 | 12.648 | 20.470 |
|  |  |  | Day 3 | 9.355^*^ | 1.436 | <.001 | 5.444 | 13.266 |
|  |  |  | Day 9 | -2.366 | 1.436 | .627 | -6.276 | 1.545 |
|  |  | Day 9 | Day 0 | 18.925^*^ | 1.436 | <.001 | 15.014 | 22.836 |
|  |  |  | Day 3 | 11.720^*^ | 1.436 | <.001 | 7.810 | 15.631 |
|  |  |  | Day 6 | 2.366 | 1.436 | .627 | -1.545 | 6.276 |
|  | 200 | Day 0 | Day 3 | -7.634^*^ | 1.436 | <.001 | -11.545 | -3.724 |
|  |  |  | Day 6 | -13.656^*^ | 1.436 | <.001 | -17.567 | -9.745 |
|  |  |  | Day 9 | -11.075^*^ | 1.436 | <.001 | -14.986 | -7.164 |
|  |  | Day 3 | Day 0 | 7.634^*^ | 1.436 | <.001 | 3.724 | 11.545 |
|  |  |  | Day 6 | -6.022^*^ | 1.436 | <.001 | -9.932 | -2.111 |
|  |  |  | Day 9 | -3.441 | 1.436 | .117 | -7.352 | .470 |
|  |  | Day 6 | Day 0 | 13.656^*^ | 1.436 | <.001 | 9.745 | 17.567 |
|  |  |  | Day 3 | 6.022^*^ | 1.436 | <.001 | 2.111 | 9.932 |
|  |  |  | Day 9 | 2.581 | 1.436 | .463 | -1.330 | 6.491 |
|  |  | Day 9 | Day 0 | 11.075^*^ | 1.436 | <.001 | 7.164 | 14.986 |
|  |  |  | Day 3 | 3.441 | 1.436 | .117 | -.470 | 7.352 |
|  |  |  | Day 6 | -2.581 | 1.436 | .463 | -6.491 | 1.330 |
|  | 300 | Day 0 | Day 3 | -14.624^*^ | 1.436 | <.001 | -18.534 | -10.713 |
|  |  |  | Day 6 | -31.398^*^ | 1.436 | <.001 | -35.309 | -27.487 |
|  |  |  | Day 9 | -34.839^*^ | 1.436 | <.001 | -38.750 | -30.928 |
|  |  | Day 3 | Day 0 | 14.624^*^ | 1.436 | <.001 | 10.713 | 18.534 |
|  |  |  | Day 6 | -16.774^*^ | 1.436 | <.001 | -20.685 | -12.863 |
|  |  |  | Day 9 | -20.215^*^ | 1.436 | <.001 | -24.126 | -16.304 |
|  |  | Day 6 | Day 0 | 31.398^*^ | 1.436 | <.001 | 27.487 | 35.309 |
|  |  |  | Day 3 | 16.774^*^ | 1.436 | <.001 | 12.863 | 20.685 |
|  |  |  | Day 9 | -3.441 | 1.436 | .117 | -7.352 | .470 |
|  |  | Day 9 | Day 0 | 34.839^*^ | 1.436 | <.001 | 30.928 | 38.750 |
|  |  |  | Day 3 | 20.215^*^ | 1.436 | <.001 | 16.304 | 24.126 |
|  |  |  | Day 6 | 3.441 | 1.436 | .117 | -.470 | 7.352 |
| Based on estimated marginal means | | | | | | | | |
| *. The mean difference is significant at the .05 level. | | | | | | | | |
| b. Adjustment for multiple comparisons: Bonferroni. | | | | | | | | |

| Univariate Tests | | | | | | | | |
| --- | --- | --- | --- | --- | --- | --- | --- | --- |
| Dependent Variable: MDA | | | | | | | | |
| Light | SNP (µM) | | Sum of Squares | df | Mean Square | F | Sig. | Partial Eta Squared |
| White light | 0 | Contrast | 3565.756 | 3 | 1188.585 | 384.078 | <.001 | .947 |
|  |  | Error | 198.058 | 64 | 3.095 |  |  |  |
|  | 100 | Contrast | 567.282 | 3 | 189.094 | 61.104 | <.001 | .741 |
|  |  | Error | 198.058 | 64 | 3.095 |  |  |  |
|  | 200 | Contrast | 384.487 | 3 | 128.162 | 41.414 | <.001 | .660 |
|  |  | Error | 198.058 | 64 | 3.095 |  |  |  |
|  | 300 | Contrast | 2606.348 | 3 | 868.783 | 280.737 | <.001 | .929 |
|  |  | Error | 198.058 | 64 | 3.095 |  |  |  |
| High blue light | 0 | Contrast | 3320.465 | 3 | 1106.822 | 357.657 | <.001 | .944 |
|  |  | Error | 198.058 | 64 | 3.095 |  |  |  |
|  | 100 | Contrast | 686.048 | 3 | 228.683 | 73.896 | <.001 | .776 |
|  |  | Error | 198.058 | 64 | 3.095 |  |  |  |
|  | 200 | Contrast | 316.641 | 3 | 105.547 | 34.106 | <.001 | .615 |
|  |  | Error | 198.058 | 64 | 3.095 |  |  |  |
|  | 300 | Contrast | 2336.455 | 3 | 778.818 | 251.666 | <.001 | .922 |
|  |  | Error | 198.058 | 64 | 3.095 |  |  |  |
| Each F tests the simple effects of Day within each level combination of the other effects shown. These tests are based on the linearly independent pairwise comparisons among the estimated marginal means. | | | | | | | | |

**Post Hoc Tests**

| **MDA** | | | | |
| --- | --- | --- | --- | --- |
| Duncan^a,b^ | | | | |
| SNP | N | Subset | | |
|  |  | 1 | 2 | 3 |
| 200 uM | 24 | 29.8387 |  |  |
| 100 uM | 24 |  | 32.1237 |  |
| 0 uM | 24 |  |  | 41.4651 |
| 300 uM | 24 |  |  | 41.6398 |
| Sig. |  | 1.000 | 1.000 | .732 |
| Means for groups in homogeneous subsets are displayed.  Based on observed means.  The error term is Mean Square(Error) = 3.095. | | | | |
| a. Uses Harmonic Mean Sample Size = 24.000. | | | | |
| b. Alpha = .05. | | | | |

| **MDA** | | | | |
| --- | --- | --- | --- | --- |
| Duncan^a,b^ | | | | |
| Day | N | Subset | | |
|  |  | 1 | 2 | 3 |
| Day 0 | 24 | 20.9140 |  |  |
| Day 3 | 24 |  | 31.9355 |  |
| Day 6 | 24 |  |  | 45.8468 |
| Day 9 | 24 |  |  | 46.3710 |
| Sig. |  | 1.000 | 1.000 | .306 |
| Means for groups in homogeneous subsets are displayed.  Based on observed means.  The error term is Mean Square(Error) = 3.095. | | | | |
| a. Uses Harmonic Mean Sample Size = 24.000. | | | | |
| b. Alpha = .05. | | | | |

**DPPH**

| Tests of Between-Subjects Effects | | | | | | |
| --- | --- | --- | --- | --- | --- | --- |
| Dependent Variable: DPPH | | | | | | |
| Source | Type III Sum of Squares | df | Mean Square | F | Sig. | Partial Eta Squared |
| Corrected Model | 15276.745^a^ | 31 | 492.798 | 56.024 | <.001 | .964 |
| Intercept | 415052.004 | 1 | 415052.004 | 47185.029 | <.001 | .999 |
| Light | 3010.952 | 1 | 3010.952 | 342.299 | <.001 | .842 |
| SNP | 215.056 | 3 | 71.685 | 8.150 | <.001 | .276 |
| Day | 10670.440 | 3 | 3556.813 | 404.355 | <.001 | .950 |
| Light * SNP | 131.863 | 3 | 43.954 | 4.997 | .004 | .190 |
| Light * Day | 713.502 | 3 | 237.834 | 27.038 | <.001 | .559 |
| SNP * Day | 337.951 | 9 | 37.550 | 4.269 | <.001 | .375 |
| Light * SNP * Day | 196.981 | 9 | 21.887 | 2.488 | .017 | .259 |
| Error | 562.961 | 64 | 8.796 |  |  |  |
| Total | 430891.710 | 96 |  |  |  |  |
| Corrected Total | 15839.706 | 95 |  |  |  |  |
| a. R Squared = .964 (Adjusted R Squared = .947) | | | | | | |

**Light * SNP**

| Pairwise Comparisons | | | | | | | |
| --- | --- | --- | --- | --- | --- | --- | --- |
| Dependent Variable: DPPH | | | | | | | |
| SNP (µM) | (I) Light | (J) Light | Mean Difference (I-J) | Std. Error | Sig.^b^ | 95% Confidence Interval for Difference^b^ | |
|  |  |  |  |  |  | Lower Bound | Upper Bound |
| 0 | White light | High blue light | -11.457^*^ | 1.211 | <.001 | -13.876 | -9.038 |
|  | High blue light | White light | 11.457^*^ | 1.211 | <.001 | 9.038 | 13.876 |
| 100 | White light | High blue light | -7.619^*^ | 1.211 | <.001 | -10.038 | -5.201 |
|  | High blue light | White light | 7.619^*^ | 1.211 | <.001 | 5.201 | 10.038 |
| 200 | White light | High blue light | -11.530^*^ | 1.211 | <.001 | -13.948 | -9.111 |
|  | High blue light | White light | 11.530^*^ | 1.211 | <.001 | 9.111 | 13.948 |
| 300 | White light | High blue light | -14.197^*^ | 1.211 | <.001 | -16.616 | -11.778 |
|  | High blue light | White light | 14.197^*^ | 1.211 | <.001 | 11.778 | 16.616 |
| Based on estimated marginal means | | | | | | | |
| *. The mean difference is significant at the .05 level. | | | | | | | |
| b. Adjustment for multiple comparisons: Bonferroni. | | | | | | | |

| Univariate Tests | | | | | | | |
| --- | --- | --- | --- | --- | --- | --- | --- |
| Dependent Variable: DPPH | | | | | | | |
| SNP (µM) | | Sum of Squares | df | Mean Square | F | Sig. | Partial Eta Squared |
| 0 | Contrast | 787.548 | 1 | 787.548 | 89.532 | <.001 | .583 |
|  | Error | 562.961 | 64 | 8.796 |  |  |  |
| 100 | Contrast | 348.337 | 1 | 348.337 | 39.601 | <.001 | .382 |
|  | Error | 562.961 | 64 | 8.796 |  |  |  |
| 200 | Contrast | 797.594 | 1 | 797.594 | 90.674 | <.001 | .586 |
|  | Error | 562.961 | 64 | 8.796 |  |  |  |
| 300 | Contrast | 1209.336 | 1 | 1209.336 | 137.483 | <.001 | .682 |
|  | Error | 562.961 | 64 | 8.796 |  |  |  |
| Each F tests the simple effects of Light within each level combination of the other effects shown. These tests are based on the linearly independent pairwise comparisons among the estimated marginal means. | | | | | | | |

| Pairwise Comparisons | | | | | | | |
| --- | --- | --- | --- | --- | --- | --- | --- |
| Dependent Variable: DPPH | | | | | | | |
| Light | (I) SNP (µM) | (J) SNP (µM) | Mean Difference (I-J) | Std. Error | Sig.^b^ | 95% Confidence Interval for Difference^b^ | |
|  |  |  |  |  |  | Lower Bound | Upper Bound |
| White light | 0 | 100 | -2.850 | 1.211 | .130 | -6.146 | .447 |
|  |  | 200 | -2.455 | 1.211 | .281 | -5.751 | .842 |
|  |  | 300 | -2.550 | 1.211 | .235 | -5.846 | .747 |
|  | 100 | 0 | 2.850 | 1.211 | .130 | -.447 | 6.146 |
|  |  | 200 | .395 | 1.211 | 1.000 | -2.902 | 3.692 |
|  |  | 300 | .300 | 1.211 | 1.000 | -2.997 | 3.597 |
|  | 200 | 0 | 2.455 | 1.211 | .281 | -.842 | 5.751 |
|  |  | 100 | -.395 | 1.211 | 1.000 | -3.692 | 2.902 |
|  |  | 300 | -.095 | 1.211 | 1.000 | -3.391 | 3.202 |
|  | 300 | 0 | 2.550 | 1.211 | .235 | -.747 | 5.846 |
|  |  | 100 | -.300 | 1.211 | 1.000 | -3.597 | 2.997 |
|  |  | 200 | .095 | 1.211 | 1.000 | -3.202 | 3.391 |
| High blue light | 0 | 100 | .988 | 1.211 | 1.000 | -2.309 | 4.284 |
|  |  | 200 | -2.528 | 1.211 | .245 | -5.824 | .769 |
|  |  | 300 | -5.290^*^ | 1.211 | <.001 | -8.586 | -1.993 |
|  | 100 | 0 | -.988 | 1.211 | 1.000 | -4.284 | 2.309 |
|  |  | 200 | -3.515^*^ | 1.211 | .030 | -6.812 | -.219 |
|  |  | 300 | -6.277^*^ | 1.211 | <.001 | -9.574 | -2.981 |
|  | 200 | 0 | 2.528 | 1.211 | .245 | -.769 | 5.824 |
|  |  | 100 | 3.515^*^ | 1.211 | .030 | .219 | 6.812 |
|  |  | 300 | -2.762 | 1.211 | .155 | -6.059 | .535 |
|  | 300 | 0 | 5.290^*^ | 1.211 | <.001 | 1.993 | 8.586 |
|  |  | 100 | 6.277^*^ | 1.211 | <.001 | 2.981 | 9.574 |
|  |  | 200 | 2.762 | 1.211 | .155 | -.535 | 6.059 |
| Based on estimated marginal means | | | | | | | |
| *. The mean difference is significant at the .05 level. | | | | | | | |
| b. Adjustment for multiple comparisons: Bonferroni. | | | | | | | |

| Univariate Tests | | | | | | | |
| --- | --- | --- | --- | --- | --- | --- | --- |
| Dependent Variable: DPPH | | | | | | | |
| Light | | Sum of Squares | df | Mean Square | F | Sig. | Partial Eta Squared |
| White light | Contrast | 62.704 | 3 | 20.901 | 2.376 | .078 | .100 |
|  | Error | 562.961 | 64 | 8.796 |  |  |  |
| High blue light | Contrast | 284.214 | 3 | 94.738 | 10.770 | <.001 | .335 |
|  | Error | 562.961 | 64 | 8.796 |  |  |  |
| Each F tests the simple effects of SNP within each level combination of the other effects shown. These tests are based on the linearly independent pairwise comparisons among the estimated marginal means. | | | | | | | |

**Light * Day**

| Pairwise Comparisons | | | | | | | |
| --- | --- | --- | --- | --- | --- | --- | --- |
| Dependent Variable: DPPH | | | | | | | |
| Day | (I) Light | (J) Light | Mean Difference (I-J) | Std. Error | Sig.^b^ | 95% Confidence Interval for Difference^b^ | |
|  |  |  |  |  |  | Lower Bound | Upper Bound |
| Day 0 | White light | High blue light | -18.844^*^ | 1.211 | <.001 | -21.263 | -16.425 |
|  | High blue light | White light | 18.844^*^ | 1.211 | <.001 | 16.425 | 21.263 |
| Day 3 | White light | High blue light | -4.617^*^ | 1.211 | <.001 | -7.035 | -2.198 |
|  | High blue light | White light | 4.617^*^ | 1.211 | <.001 | 2.198 | 7.035 |
| Day 6 | White light | High blue light | -13.551^*^ | 1.211 | <.001 | -15.970 | -11.132 |
|  | High blue light | White light | 13.551^*^ | 1.211 | <.001 | 11.132 | 15.970 |
| Day 9 | White light | High blue light | -7.791^*^ | 1.211 | <.001 | -10.210 | -5.373 |
|  | High blue light | White light | 7.791^*^ | 1.211 | <.001 | 5.373 | 10.210 |
| Based on estimated marginal means | | | | | | | |
| *. The mean difference is significant at the .05 level. | | | | | | | |
| b. Adjustment for multiple comparisons: Bonferroni. | | | | | | | |

| Univariate Tests | | | | | | | |
| --- | --- | --- | --- | --- | --- | --- | --- |
| Dependent Variable: DPPH | | | | | | | |
| Day | | Sum of Squares | df | Mean Square | F | Sig. | Partial Eta Squared |
| Day 0 | Contrast | 2130.540 | 1 | 2130.540 | 242.210 | <.001 | .791 |
|  | Error | 562.961 | 64 | 8.796 |  |  |  |
| Day 3 | Contrast | 127.872 | 1 | 127.872 | 14.537 | <.001 | .185 |
|  | Error | 562.961 | 64 | 8.796 |  |  |  |
| Day 6 | Contrast | 1101.805 | 1 | 1101.805 | 125.258 | <.001 | .662 |
|  | Error | 562.961 | 64 | 8.796 |  |  |  |
| Day 9 | Contrast | 364.237 | 1 | 364.237 | 41.408 | <.001 | .393 |
|  | Error | 562.961 | 64 | 8.796 |  |  |  |
| Each F tests the simple effects of Light within each level combination of the other effects shown. These tests are based on the linearly independent pairwise comparisons among the estimated marginal means. | | | | | | | |

| Pairwise Comparisons | | | | | | | |
| --- | --- | --- | --- | --- | --- | --- | --- |
| Dependent Variable: DPPH | | | | | | | |
| Light | (I) Day | (J) Day | Mean Difference (I-J) | Std. Error | Sig.^b^ | 95% Confidence Interval for Difference^b^ | |
|  |  |  |  |  |  | Lower Bound | Upper Bound |
| White light | Day 0 | Day 3 | 7.119^*^ | 1.211 | <.001 | 3.822 | 10.416 |
|  |  | Day 6 | 16.603^*^ | 1.211 | <.001 | 13.307 | 19.900 |
|  |  | Day 9 | 23.715^*^ | 1.211 | <.001 | 20.418 | 27.012 |
|  | Day 3 | Day 0 | -7.119^*^ | 1.211 | <.001 | -10.416 | -3.822 |
|  |  | Day 6 | 9.484^*^ | 1.211 | <.001 | 6.188 | 12.781 |
|  |  | Day 9 | 16.596^*^ | 1.211 | <.001 | 13.299 | 19.892 |
|  | Day 6 | Day 0 | -16.603^*^ | 1.211 | <.001 | -19.900 | -13.307 |
|  |  | Day 3 | -9.484^*^ | 1.211 | <.001 | -12.781 | -6.188 |
|  |  | Day 9 | 7.111^*^ | 1.211 | <.001 | 3.815 | 10.408 |
|  | Day 9 | Day 0 | -23.715^*^ | 1.211 | <.001 | -27.012 | -20.418 |
|  |  | Day 3 | -16.596^*^ | 1.211 | <.001 | -19.892 | -13.299 |
|  |  | Day 6 | -7.111^*^ | 1.211 | <.001 | -10.408 | -3.815 |
| High blue light | Day 0 | Day 3 | 21.347^*^ | 1.211 | <.001 | 18.050 | 24.643 |
|  |  | Day 6 | 21.896^*^ | 1.211 | <.001 | 18.599 | 25.193 |
|  |  | Day 9 | 34.767^*^ | 1.211 | <.001 | 31.471 | 38.064 |
|  | Day 3 | Day 0 | -21.347^*^ | 1.211 | <.001 | -24.643 | -18.050 |
|  |  | Day 6 | .550 | 1.211 | 1.000 | -2.747 | 3.846 |
|  |  | Day 9 | 13.421^*^ | 1.211 | <.001 | 10.124 | 16.717 |
|  | Day 6 | Day 0 | -21.896^*^ | 1.211 | <.001 | -25.193 | -18.599 |
|  |  | Day 3 | -.550 | 1.211 | 1.000 | -3.846 | 2.747 |
|  |  | Day 9 | 12.871^*^ | 1.211 | <.001 | 9.574 | 16.168 |
|  | Day 9 | Day 0 | -34.767^*^ | 1.211 | <.001 | -38.064 | -31.471 |
|  |  | Day 3 | -13.421^*^ | 1.211 | <.001 | -16.717 | -10.124 |
|  |  | Day 6 | -12.871^*^ | 1.211 | <.001 | -16.168 | -9.574 |
| Based on estimated marginal means | | | | | | | |
| *. The mean difference is significant at the .05 level. | | | | | | | |
| b. Adjustment for multiple comparisons: Bonferroni. | | | | | | | |

| Univariate Tests | | | | | | | |
| --- | --- | --- | --- | --- | --- | --- | --- |
| Dependent Variable: DPPH | | | | | | | |
| Light | | Sum of Squares | df | Mean Square | F | Sig. | Partial Eta Squared |
| White light | Contrast | 3914.066 | 3 | 1304.689 | 148.323 | <.001 | .874 |
|  | Error | 562.961 | 64 | 8.796 |  |  |  |
| High blue light | Contrast | 7469.876 | 3 | 2489.959 | 283.070 | <.001 | .930 |
|  | Error | 562.961 | 64 | 8.796 |  |  |  |
| Each F tests the simple effects of Day within each level combination of the other effects shown. These tests are based on the linearly independent pairwise comparisons among the estimated marginal means. | | | | | | | |

**SNP * Day**

| Pairwise Comparisons | | | | | | | |
| --- | --- | --- | --- | --- | --- | --- | --- |
| Dependent Variable: DPPH | | | | | | | |
| Day | (I) SNP (µM) | (J) SNP (µM) | Mean Difference (I-J) | Std. Error | Sig.^b^ | 95% Confidence Interval for Difference^b^ | |
|  |  |  |  |  |  | Lower Bound | Upper Bound |
| Day 0 | 0 | 100 | .000 | 1.712 | 1.000 | -4.662 | 4.662 |
|  |  | 200 | -4.441E-16 | 1.712 | 1.000 | -4.662 | 4.662 |
|  |  | 300 | -7.594E-14 | 1.712 | 1.000 | -4.662 | 4.662 |
|  | 100 | 0 | .000 | 1.712 | 1.000 | -4.662 | 4.662 |
|  |  | 200 | -4.441E-16 | 1.712 | 1.000 | -4.662 | 4.662 |
|  |  | 300 | -7.594E-14 | 1.712 | 1.000 | -4.662 | 4.662 |
|  | 200 | 0 | 4.441E-16 | 1.712 | 1.000 | -4.662 | 4.662 |
|  |  | 100 | 4.441E-16 | 1.712 | 1.000 | -4.662 | 4.662 |
|  |  | 300 | -7.550E-14 | 1.712 | 1.000 | -4.662 | 4.662 |
|  | 300 | 0 | 7.594E-14 | 1.712 | 1.000 | -4.662 | 4.662 |
|  |  | 100 | 7.594E-14 | 1.712 | 1.000 | -4.662 | 4.662 |
|  |  | 200 | 7.550E-14 | 1.712 | 1.000 | -4.662 | 4.662 |
| Day 3 | 0 | 100 | 1.298 | 1.712 | 1.000 | -3.364 | 5.961 |
|  |  | 200 | -4.261 | 1.712 | .093 | -8.923 | .402 |
|  |  | 300 | -9.235^*^ | 1.712 | <.001 | -13.897 | -4.572 |
|  | 100 | 0 | -1.298 | 1.712 | 1.000 | -5.961 | 3.364 |
|  |  | 200 | -5.559^*^ | 1.712 | .011 | -10.221 | -.897 |
|  |  | 300 | -10.533^*^ | 1.712 | <.001 | -15.195 | -5.871 |
|  | 200 | 0 | 4.261 | 1.712 | .093 | -.402 | 8.923 |
|  |  | 100 | 5.559^*^ | 1.712 | .011 | .897 | 10.221 |
|  |  | 300 | -4.974^*^ | 1.712 | .030 | -9.636 | -.312 |
|  | 300 | 0 | 9.235^*^ | 1.712 | <.001 | 4.572 | 13.897 |
|  |  | 100 | 10.533^*^ | 1.712 | <.001 | 5.871 | 15.195 |
|  |  | 200 | 4.974^*^ | 1.712 | .030 | .312 | 9.636 |
| Day 6 | 0 | 100 | -4.628 | 1.712 | .053 | -9.291 | .034 |
|  |  | 200 | -6.117^*^ | 1.712 | .004 | -10.779 | -1.455 |
|  |  | 300 | -5.581^*^ | 1.712 | .011 | -10.244 | -.919 |
|  | 100 | 0 | 4.628 | 1.712 | .053 | -.034 | 9.291 |
|  |  | 200 | -1.488 | 1.712 | 1.000 | -6.151 | 3.174 |
|  |  | 300 | -.953 | 1.712 | 1.000 | -5.615 | 3.709 |
|  | 200 | 0 | 6.117^*^ | 1.712 | .004 | 1.455 | 10.779 |
|  |  | 100 | 1.488 | 1.712 | 1.000 | -3.174 | 6.151 |
|  |  | 300 | .536 | 1.712 | 1.000 | -4.127 | 5.198 |
|  | 300 | 0 | 5.581^*^ | 1.712 | .011 | .919 | 10.244 |
|  |  | 100 | .953 | 1.712 | 1.000 | -3.709 | 5.615 |
|  |  | 200 | -.536 | 1.712 | 1.000 | -5.198 | 4.127 |
| Day 9 | 0 | 100 | -.394 | 1.712 | 1.000 | -5.056 | 4.268 |
|  |  | 200 | .413 | 1.712 | 1.000 | -4.249 | 5.075 |
|  |  | 300 | -.863 | 1.712 | 1.000 | -5.525 | 3.800 |
|  | 100 | 0 | .394 | 1.712 | 1.000 | -4.268 | 5.056 |
|  |  | 200 | .807 | 1.712 | 1.000 | -3.856 | 5.469 |
|  |  | 300 | -.469 | 1.712 | 1.000 | -5.131 | 4.194 |
|  | 200 | 0 | -.413 | 1.712 | 1.000 | -5.075 | 4.249 |
|  |  | 100 | -.807 | 1.712 | 1.000 | -5.469 | 3.856 |
|  |  | 300 | -1.275 | 1.712 | 1.000 | -5.938 | 3.387 |
|  | 300 | 0 | .863 | 1.712 | 1.000 | -3.800 | 5.525 |
|  |  | 100 | .469 | 1.712 | 1.000 | -4.194 | 5.131 |
|  |  | 200 | 1.275 | 1.712 | 1.000 | -3.387 | 5.938 |
| Based on estimated marginal means | | | | | | | |
| *. The mean difference is significant at the .05 level. | | | | | | | |
| b. Adjustment for multiple comparisons: Bonferroni. | | | | | | | |

| Univariate Tests | | | | | | | |
| --- | --- | --- | --- | --- | --- | --- | --- |
| Dependent Variable: DPPH | | | | | | | |
| Day | | Sum of Squares | df | Mean Square | F | Sig. | Partial Eta Squared |
| Day 0 | Contrast | 2.585E-26 | 3 | 8.617E-27 | .000 | 1.000 | .000 |
|  | Error | 562.961 | 64 | 8.796 |  |  |  |
| Day 3 | Contrast | 407.558 | 3 | 135.853 | 15.444 | <.001 | .420 |
|  | Error | 562.961 | 64 | 8.796 |  |  |  |
| Day 6 | Contrast | 140.100 | 3 | 46.700 | 5.309 | .002 | .199 |
|  | Error | 562.961 | 64 | 8.796 |  |  |  |
| Day 9 | Contrast | 5.349 | 3 | 1.783 | .203 | .894 | .009 |
|  | Error | 562.961 | 64 | 8.796 |  |  |  |
| Each F tests the simple effects of SNP within each level combination of the other effects shown. These tests are based on the linearly independent pairwise comparisons among the estimated marginal means. | | | | | | | |

| Pairwise Comparisons | | | | | | | |
| --- | --- | --- | --- | --- | --- | --- | --- |
| Dependent Variable: DPPH | | | | | | | |
| SNP (µM) | (I) Day | (J) Day | Mean Difference (I-J) | Std. Error | Sig.^b^ | 95% Confidence Interval for Difference^b^ | |
|  |  |  |  |  |  | Lower Bound | Upper Bound |
| 0 | Day 0 | Day 3 | 17.282^*^ | 1.712 | <.001 | 12.620 | 21.944 |
|  |  | Day 6 | 23.331^*^ | 1.712 | <.001 | 18.669 | 27.994 |
|  |  | Day 9 | 29.452^*^ | 1.712 | <.001 | 24.790 | 34.114 |
|  | Day 3 | Day 0 | -17.282^*^ | 1.712 | <.001 | -21.944 | -12.620 |
|  |  | Day 6 | 6.049^*^ | 1.712 | .005 | 1.387 | 10.712 |
|  |  | Day 9 | 12.170^*^ | 1.712 | <.001 | 7.508 | 16.832 |
|  | Day 6 | Day 0 | -23.331^*^ | 1.712 | <.001 | -27.994 | -18.669 |
|  |  | Day 3 | -6.049^*^ | 1.712 | .005 | -10.712 | -1.387 |
|  |  | Day 9 | 6.121^*^ | 1.712 | .004 | 1.458 | 10.783 |
|  | Day 9 | Day 0 | -29.452^*^ | 1.712 | <.001 | -34.114 | -24.790 |
|  |  | Day 3 | -12.170^*^ | 1.712 | <.001 | -16.832 | -7.508 |
|  |  | Day 6 | -6.121^*^ | 1.712 | .004 | -10.783 | -1.458 |
| 100 | Day 0 | Day 3 | 18.580^*^ | 1.712 | <.001 | 13.918 | 23.243 |
|  |  | Day 6 | 18.703^*^ | 1.712 | <.001 | 14.041 | 23.365 |
|  |  | Day 9 | 29.058^*^ | 1.712 | <.001 | 24.396 | 33.720 |
|  | Day 3 | Day 0 | -18.580^*^ | 1.712 | <.001 | -23.243 | -13.918 |
|  |  | Day 6 | .122 | 1.712 | 1.000 | -4.540 | 4.785 |
|  |  | Day 9 | 10.478^*^ | 1.712 | <.001 | 5.815 | 15.140 |
|  | Day 6 | Day 0 | -18.703^*^ | 1.712 | <.001 | -23.365 | -14.041 |
|  |  | Day 3 | -.122 | 1.712 | 1.000 | -4.785 | 4.540 |
|  |  | Day 9 | 10.355^*^ | 1.712 | <.001 | 5.693 | 15.017 |
|  | Day 9 | Day 0 | -29.058^*^ | 1.712 | <.001 | -33.720 | -24.396 |
|  |  | Day 3 | -10.478^*^ | 1.712 | <.001 | -15.140 | -5.815 |
|  |  | Day 6 | -10.355^*^ | 1.712 | <.001 | -15.017 | -5.693 |
| 200 | Day 0 | Day 3 | 13.021^*^ | 1.712 | <.001 | 8.359 | 17.684 |
|  |  | Day 6 | 17.215^*^ | 1.712 | <.001 | 12.552 | 21.877 |
|  |  | Day 9 | 29.865^*^ | 1.712 | <.001 | 25.203 | 34.527 |
|  | Day 3 | Day 0 | -13.021^*^ | 1.712 | <.001 | -17.684 | -8.359 |
|  |  | Day 6 | 4.193 | 1.712 | .102 | -.469 | 8.855 |
|  |  | Day 9 | 16.843^*^ | 1.712 | <.001 | 12.181 | 21.506 |
|  | Day 6 | Day 0 | -17.215^*^ | 1.712 | <.001 | -21.877 | -12.552 |
|  |  | Day 3 | -4.193 | 1.712 | .102 | -8.855 | .469 |
|  |  | Day 9 | 12.650^*^ | 1.712 | <.001 | 7.988 | 17.312 |
|  | Day 9 | Day 0 | -29.865^*^ | 1.712 | <.001 | -34.527 | -25.203 |
|  |  | Day 3 | -16.843^*^ | 1.712 | <.001 | -21.506 | -12.181 |
|  |  | Day 6 | -12.650^*^ | 1.712 | <.001 | -17.312 | -7.988 |
| 300 | Day 0 | Day 3 | 8.047^*^ | 1.712 | <.001 | 3.385 | 12.710 |
|  |  | Day 6 | 17.750^*^ | 1.712 | <.001 | 13.088 | 22.412 |
|  |  | Day 9 | 28.589^*^ | 1.712 | <.001 | 23.927 | 33.252 |
|  | Day 3 | Day 0 | -8.047^*^ | 1.712 | <.001 | -12.710 | -3.385 |
|  |  | Day 6 | 9.703^*^ | 1.712 | <.001 | 5.040 | 14.365 |
|  |  | Day 9 | 20.542^*^ | 1.712 | <.001 | 15.880 | 25.204 |
|  | Day 6 | Day 0 | -17.750^*^ | 1.712 | <.001 | -22.412 | -13.088 |
|  |  | Day 3 | -9.703^*^ | 1.712 | <.001 | -14.365 | -5.040 |
|  |  | Day 9 | 10.839^*^ | 1.712 | <.001 | 6.177 | 15.502 |
|  | Day 9 | Day 0 | -28.589^*^ | 1.712 | <.001 | -33.252 | -23.927 |
|  |  | Day 3 | -20.542^*^ | 1.712 | <.001 | -25.204 | -15.880 |
|  |  | Day 6 | -10.839^*^ | 1.712 | <.001 | -15.502 | -6.177 |
| Based on estimated marginal means | | | | | | | |
| *. The mean difference is significant at the .05 level. | | | | | | | |
| b. Adjustment for multiple comparisons: Bonferroni. | | | | | | | |

| Univariate Tests | | | | | | | |
| --- | --- | --- | --- | --- | --- | --- | --- |
| Dependent Variable: DPPH | | | | | | | |
| SNP (µM) | | Sum of Squares | df | Mean Square | F | Sig. | Partial Eta Squared |
| 0 | Contrast | 2898.901 | 3 | 966.300 | 109.853 | <.001 | .837 |
|  | Error | 562.961 | 64 | 8.796 |  |  |  |
| 100 | Contrast | 2634.644 | 3 | 878.215 | 99.839 | <.001 | .824 |
|  | Error | 562.961 | 64 | 8.796 |  |  |  |
| 200 | Contrast | 2728.665 | 3 | 909.555 | 103.402 | <.001 | .829 |
|  | Error | 562.961 | 64 | 8.796 |  |  |  |
| 300 | Contrast | 2746.182 | 3 | 915.394 | 104.066 | <.001 | .830 |
|  | Error | 562.961 | 64 | 8.796 |  |  |  |
| Each F tests the simple effects of Day within each level combination of the other effects shown. These tests are based on the linearly independent pairwise comparisons among the estimated marginal means. | | | | | | | |

**Light * SNP * Day**

| Pairwise Comparisons | | | | | | | | |
| --- | --- | --- | --- | --- | --- | --- | --- | --- |
| Dependent Variable: DPPH | | | | | | | | |
| SNP (µM) | Day | (I) Light | (J) Light | Mean Difference (I-J) | Std. Error | Sig.^b^ | 95% Confidence Interval for Difference^b^ | |
|  |  |  |  |  |  |  | Lower Bound | Upper Bound |
| 0 | Day 0 | White light | High blue light | -18.844^*^ | 2.422 | <.001 | -23.682 | -14.006 |
|  |  | High blue light | White light | 18.844^*^ | 2.422 | <.001 | 14.006 | 23.682 |
|  | Day 3 | White light | High blue light | .045 | 2.422 | .985 | -4.793 | 4.883 |
|  |  | High blue light | White light | -.045 | 2.422 | .985 | -4.883 | 4.793 |
|  | Day 6 | White light | High blue light | -18.320^*^ | 2.422 | <.001 | -23.157 | -13.482 |
|  |  | High blue light | White light | 18.320^*^ | 2.422 | <.001 | 13.482 | 23.157 |
|  | Day 9 | White light | High blue light | -8.709^*^ | 2.422 | <.001 | -13.546 | -3.871 |
|  |  | High blue light | White light | 8.709^*^ | 2.422 | <.001 | 3.871 | 13.546 |
| 100 | Day 0 | White light | High blue light | -18.844^*^ | 2.422 | <.001 | -23.682 | -14.006 |
|  |  | High blue light | White light | 18.844^*^ | 2.422 | <.001 | 14.006 | 23.682 |
|  | Day 3 | White light | High blue light | 2.651 | 2.422 | .278 | -2.187 | 7.489 |
|  |  | High blue light | White light | -2.651 | 2.422 | .278 | -7.489 | 2.187 |
|  | Day 6 | White light | High blue light | -9.385^*^ | 2.422 | <.001 | -14.222 | -4.547 |
|  |  | High blue light | White light | 9.385^*^ | 2.422 | <.001 | 4.547 | 14.222 |
|  | Day 9 | White light | High blue light | -4.900^*^ | 2.422 | .047 | -9.738 | -.063 |
|  |  | High blue light | White light | 4.900^*^ | 2.422 | .047 | .063 | 9.738 |
| 200 | Day 0 | White light | High blue light | -18.844^*^ | 2.422 | <.001 | -23.682 | -14.006 |
|  |  | High blue light | White light | 18.844^*^ | 2.422 | <.001 | 14.006 | 23.682 |
|  | Day 3 | White light | High blue light | -9.632^*^ | 2.422 | <.001 | -14.470 | -4.795 |
|  |  | High blue light | White light | 9.632^*^ | 2.422 | <.001 | 4.795 | 14.470 |
|  | Day 6 | White light | High blue light | -10.541^*^ | 2.422 | <.001 | -15.378 | -5.703 |
|  |  | High blue light | White light | 10.541^*^ | 2.422 | <.001 | 5.703 | 15.378 |
|  | Day 9 | White light | High blue light | -7.102^*^ | 2.422 | .005 | -11.939 | -2.264 |
|  |  | High blue light | White light | 7.102^*^ | 2.422 | .005 | 2.264 | 11.939 |
| 300 | Day 0 | White light | High blue light | -18.844^*^ | 2.422 | <.001 | -23.682 | -14.006 |
|  |  | High blue light | White light | 18.844^*^ | 2.422 | <.001 | 14.006 | 23.682 |
|  | Day 3 | White light | High blue light | -11.530^*^ | 2.422 | <.001 | -16.367 | -6.692 |
|  |  | High blue light | White light | 11.530^*^ | 2.422 | <.001 | 6.692 | 16.367 |
|  | Day 6 | White light | High blue light | -15.960^*^ | 2.422 | <.001 | -20.797 | -11.122 |
|  |  | High blue light | White light | 15.960^*^ | 2.422 | <.001 | 11.122 | 20.797 |
|  | Day 9 | White light | High blue light | -10.455^*^ | 2.422 | <.001 | -15.293 | -5.617 |
|  |  | High blue light | White light | 10.455^*^ | 2.422 | <.001 | 5.617 | 15.293 |
| Based on estimated marginal means | | | | | | | | |
| *. The mean difference is significant at the .05 level. | | | | | | | | |
| b. Adjustment for multiple comparisons: Bonferroni. | | | | | | | | |

| Univariate Tests | | | | | | | | |
| --- | --- | --- | --- | --- | --- | --- | --- | --- |
| Dependent Variable: DPPH | | | | | | | | |
| SNP (µM) | Day | | Sum of Squares | df | Mean Square | F | Sig. | Partial Eta Squared |
| 0 | Day 0 | Contrast | 532.635 | 1 | 532.635 | 60.552 | <.001 | .486 |
|  |  | Error | 562.961 | 64 | 8.796 |  |  |  |
|  | Day 3 | Contrast | .003 | 1 | .003 | .000 | .985 | .000 |
|  |  | Error | 562.961 | 64 | 8.796 |  |  |  |
|  | Day 6 | Contrast | 503.415 | 1 | 503.415 | 57.231 | <.001 | .472 |
|  |  | Error | 562.961 | 64 | 8.796 |  |  |  |
|  | Day 9 | Contrast | 113.761 | 1 | 113.761 | 12.933 | <.001 | .168 |
|  |  | Error | 562.961 | 64 | 8.796 |  |  |  |
| 100 | Day 0 | Contrast | 532.635 | 1 | 532.635 | 60.552 | <.001 | .486 |
|  |  | Error | 562.961 | 64 | 8.796 |  |  |  |
|  | Day 3 | Contrast | 10.542 | 1 | 10.542 | 1.198 | .278 | .018 |
|  |  | Error | 562.961 | 64 | 8.796 |  |  |  |
|  | Day 6 | Contrast | 132.108 | 1 | 132.108 | 15.019 | <.001 | .190 |
|  |  | Error | 562.961 | 64 | 8.796 |  |  |  |
|  | Day 9 | Contrast | 36.020 | 1 | 36.020 | 4.095 | .047 | .060 |
|  |  | Error | 562.961 | 64 | 8.796 |  |  |  |
| 200 | Day 0 | Contrast | 532.635 | 1 | 532.635 | 60.552 | <.001 | .486 |
|  |  | Error | 562.961 | 64 | 8.796 |  |  |  |
|  | Day 3 | Contrast | 139.173 | 1 | 139.173 | 15.822 | <.001 | .198 |
|  |  | Error | 562.961 | 64 | 8.796 |  |  |  |
|  | Day 6 | Contrast | 166.658 | 1 | 166.658 | 18.947 | <.001 | .228 |
|  |  | Error | 562.961 | 64 | 8.796 |  |  |  |
|  | Day 9 | Contrast | 75.651 | 1 | 75.651 | 8.600 | .005 | .118 |
|  |  | Error | 562.961 | 64 | 8.796 |  |  |  |
| 300 | Day 0 | Contrast | 532.635 | 1 | 532.635 | 60.552 | <.001 | .486 |
|  |  | Error | 562.961 | 64 | 8.796 |  |  |  |
|  | Day 3 | Contrast | 199.400 | 1 | 199.400 | 22.669 | <.001 | .262 |
|  |  | Error | 562.961 | 64 | 8.796 |  |  |  |
|  | Day 6 | Contrast | 382.066 | 1 | 382.066 | 43.435 | <.001 | .404 |
|  |  | Error | 562.961 | 64 | 8.796 |  |  |  |
|  | Day 9 | Contrast | 163.961 | 1 | 163.961 | 18.640 | <.001 | .226 |
|  |  | Error | 562.961 | 64 | 8.796 |  |  |  |
| Each F tests the simple effects of Light within each level combination of the other effects shown. These tests are based on the linearly independent pairwise comparisons among the estimated marginal means. | | | | | | | | |

| Pairwise Comparisons | | | | | | | | |
| --- | --- | --- | --- | --- | --- | --- | --- | --- |
| Dependent Variable: DPPH | | | | | | | | |
| Light | Day | (I) SNP (µM) | (J) SNP (µM) | Mean Difference (I-J) | Std. Error | Sig.^b^ | 95% Confidence Interval for Difference^b^ | |
|  |  |  |  |  |  |  | Lower Bound | Upper Bound |
| White light | Day 0 | 0 | 100 | .000 | 2.422 | 1.000 | -6.593 | 6.593 |
|  |  |  | 200 | -8.882E-16 | 2.422 | 1.000 | -6.593 | 6.593 |
|  |  |  | 300 | -3.797E-14 | 2.422 | 1.000 | -6.593 | 6.593 |
|  |  | 100 | 0 | .000 | 2.422 | 1.000 | -6.593 | 6.593 |
|  |  |  | 200 | -4.441E-16 | 2.422 | 1.000 | -6.593 | 6.593 |
|  |  |  | 300 | -3.775E-14 | 2.422 | 1.000 | -6.593 | 6.593 |
|  |  | 200 | 0 | 8.882E-16 | 2.422 | 1.000 | -6.593 | 6.593 |
|  |  |  | 100 | 4.441E-16 | 2.422 | 1.000 | -6.593 | 6.593 |
|  |  |  | 300 | -3.730E-14 | 2.422 | 1.000 | -6.593 | 6.593 |
|  |  | 300 | 0 | 3.797E-14 | 2.422 | 1.000 | -6.593 | 6.593 |
|  |  |  | 100 | 3.775E-14 | 2.422 | 1.000 | -6.593 | 6.593 |
|  |  |  | 200 | 3.730E-14 | 2.422 | 1.000 | -6.593 | 6.593 |
|  | Day 3 | 0 | 100 | -.005 | 2.422 | 1.000 | -6.598 | 6.589 |
|  |  |  | 200 | .578 | 2.422 | 1.000 | -6.015 | 7.171 |
|  |  |  | 300 | -3.447 | 2.422 | .957 | -10.041 | 3.146 |
|  |  | 100 | 0 | .005 | 2.422 | 1.000 | -6.589 | 6.598 |
|  |  |  | 200 | .583 | 2.422 | 1.000 | -6.011 | 7.176 |
|  |  |  | 300 | -3.443 | 2.422 | .960 | -10.036 | 3.151 |
|  |  | 200 | 0 | -.578 | 2.422 | 1.000 | -7.171 | 6.015 |
|  |  |  | 100 | -.583 | 2.422 | 1.000 | -7.176 | 6.011 |
|  |  |  | 300 | -4.025 | 2.422 | .608 | -10.619 | 2.568 |
|  |  | 300 | 0 | 3.447 | 2.422 | .957 | -3.146 | 10.041 |
|  |  |  | 100 | 3.443 | 2.422 | .960 | -3.151 | 10.036 |
|  |  |  | 200 | 4.025 | 2.422 | .608 | -2.568 | 10.619 |
|  | Day 6 | 0 | 100 | -9.096^*^ | 2.422 | .002 | -15.689 | -2.503 |
|  |  |  | 200 | -10.006^*^ | 2.422 | <.001 | -16.600 | -3.413 |
|  |  |  | 300 | -6.761^*^ | 2.422 | .041 | -13.355 | -.168 |
|  |  | 100 | 0 | 9.096^*^ | 2.422 | .002 | 2.503 | 15.689 |
|  |  |  | 200 | -.910 | 2.422 | 1.000 | -7.504 | 5.683 |
|  |  |  | 300 | 2.335 | 2.422 | 1.000 | -4.259 | 8.928 |
|  |  | 200 | 0 | 10.006^*^ | 2.422 | <.001 | 3.413 | 16.600 |
|  |  |  | 100 | .910 | 2.422 | 1.000 | -5.683 | 7.504 |
|  |  |  | 300 | 3.245 | 2.422 | 1.000 | -3.348 | 9.838 |
|  |  | 300 | 0 | 6.761^*^ | 2.422 | .041 | .168 | 13.355 |
|  |  |  | 100 | -2.335 | 2.422 | 1.000 | -8.928 | 4.259 |
|  |  |  | 200 | -3.245 | 2.422 | 1.000 | -9.838 | 3.348 |
|  | Day 9 | 0 | 100 | -2.298 | 2.422 | 1.000 | -8.891 | 4.295 |
|  |  |  | 200 | -.391 | 2.422 | 1.000 | -6.984 | 6.203 |
|  |  |  | 300 | .011 | 2.422 | 1.000 | -6.583 | 6.604 |
|  |  | 100 | 0 | 2.298 | 2.422 | 1.000 | -4.295 | 8.891 |
|  |  |  | 200 | 1.907 | 2.422 | 1.000 | -4.686 | 8.501 |
|  |  |  | 300 | 2.309 | 2.422 | 1.000 | -4.285 | 8.902 |
|  |  | 200 | 0 | .391 | 2.422 | 1.000 | -6.203 | 6.984 |
|  |  |  | 100 | -1.907 | 2.422 | 1.000 | -8.501 | 4.686 |
|  |  |  | 300 | .401 | 2.422 | 1.000 | -6.192 | 6.995 |
|  |  | 300 | 0 | -.011 | 2.422 | 1.000 | -6.604 | 6.583 |
|  |  |  | 100 | -2.309 | 2.422 | 1.000 | -8.902 | 4.285 |
|  |  |  | 200 | -.401 | 2.422 | 1.000 | -6.995 | 6.192 |
| High blue light | Day 0 | 0 | 100 | .000 | 2.422 | 1.000 | -6.593 | 6.593 |
|  |  |  | 200 | .000 | 2.422 | 1.000 | -6.593 | 6.593 |
|  |  |  | 300 | -1.139E-13 | 2.422 | 1.000 | -6.593 | 6.593 |
|  |  | 100 | 0 | .000 | 2.422 | 1.000 | -6.593 | 6.593 |
|  |  |  | 200 | -8.882E-16 | 2.422 | 1.000 | -6.593 | 6.593 |
|  |  |  | 300 | -1.141E-13 | 2.422 | 1.000 | -6.593 | 6.593 |
|  |  | 200 | 0 | .000 | 2.422 | 1.000 | -6.593 | 6.593 |
|  |  |  | 100 | 8.882E-16 | 2.422 | 1.000 | -6.593 | 6.593 |
|  |  |  | 300 | -1.137E-13 | 2.422 | 1.000 | -6.593 | 6.593 |
|  |  | 300 | 0 | 1.139E-13 | 2.422 | 1.000 | -6.593 | 6.593 |
|  |  |  | 100 | 1.141E-13 | 2.422 | 1.000 | -6.593 | 6.593 |
|  |  |  | 200 | 1.137E-13 | 2.422 | 1.000 | -6.593 | 6.593 |
|  | Day 3 | 0 | 100 | 2.601 | 2.422 | 1.000 | -3.992 | 9.195 |
|  |  |  | 200 | -9.099^*^ | 2.422 | .002 | -15.693 | -2.506 |
|  |  |  | 300 | -15.022^*^ | 2.422 | <.001 | -21.615 | -8.429 |
|  |  | 100 | 0 | -2.601 | 2.422 | 1.000 | -9.195 | 3.992 |
|  |  |  | 200 | -11.701^*^ | 2.422 | <.001 | -18.294 | -5.107 |
|  |  |  | 300 | -17.623^*^ | 2.422 | <.001 | -24.217 | -11.030 |
|  |  | 200 | 0 | 9.099^*^ | 2.422 | .002 | 2.506 | 15.693 |
|  |  |  | 100 | 11.701^*^ | 2.422 | <.001 | 5.107 | 18.294 |
|  |  |  | 300 | -5.923 | 2.422 | .103 | -12.516 | .671 |
|  |  | 300 | 0 | 15.022^*^ | 2.422 | <.001 | 8.429 | 21.615 |
|  |  |  | 100 | 17.623^*^ | 2.422 | <.001 | 11.030 | 24.217 |
|  |  |  | 200 | 5.923 | 2.422 | .103 | -.671 | 12.516 |
|  | Day 6 | 0 uM | 100 | -.161 | 2.422 | 1.000 | -6.754 | 6.432 |
|  |  |  | 200 | -2.227 | 2.422 | 1.000 | -8.821 | 4.366 |
|  |  |  | 300 | -4.401 | 2.422 | .443 | -10.995 | 2.192 |
|  |  | 100 | 0 | .161 | 2.422 | 1.000 | -6.432 | 6.754 |
|  |  |  | 200 | -2.066 | 2.422 | 1.000 | -8.660 | 4.527 |
|  |  |  | 300 | -4.240 | 2.422 | .508 | -10.834 | 2.353 |
|  |  | 200 | 0 | 2.227 | 2.422 | 1.000 | -4.366 | 8.821 |
|  |  |  | 100 | 2.066 | 2.422 | 1.000 | -4.527 | 8.660 |
|  |  |  | 300 | -2.174 | 2.422 | 1.000 | -8.767 | 4.419 |
|  |  | 300 | 0 | 4.401 | 2.422 | .443 | -2.192 | 10.995 |
|  |  |  | 100 | 4.240 | 2.422 | .508 | -2.353 | 10.834 |
|  |  |  | 200 | 2.174 | 2.422 | 1.000 | -4.419 | 8.767 |
|  | Day 9 | 0 | 100 | 1.510 | 2.422 | 1.000 | -5.083 | 8.104 |
|  |  |  | 200 | 1.216 | 2.422 | 1.000 | -5.377 | 7.810 |
|  |  |  | 300 | -1.736 | 2.422 | 1.000 | -8.329 | 4.858 |
|  |  | 100 | 0 | -1.510 | 2.422 | 1.000 | -8.104 | 5.083 |
|  |  |  | 200 | -.294 | 2.422 | 1.000 | -6.887 | 6.299 |
|  |  |  | 300 | -3.246 | 2.422 | 1.000 | -9.839 | 3.347 |
|  |  | 200 | 0 | -1.216 | 2.422 | 1.000 | -7.810 | 5.377 |
|  |  |  | 100 | .294 | 2.422 | 1.000 | -6.299 | 6.887 |
|  |  |  | 300 | -2.952 | 2.422 | 1.000 | -9.545 | 3.641 |
|  |  | 300 | 0 | 1.736 | 2.422 | 1.000 | -4.858 | 8.329 |
|  |  |  | 100 | 3.246 | 2.422 | 1.000 | -3.347 | 9.839 |
|  |  |  | 200 | 2.952 | 2.422 | 1.000 | -3.641 | 9.545 |
| Based on estimated marginal means | | | | | | | | |
| *. The mean difference is significant at the .05 level. | | | | | | | | |
| b. Adjustment for multiple comparisons: Bonferroni. | | | | | | | | |

| Univariate Tests | | | | | | | | |
| --- | --- | --- | --- | --- | --- | --- | --- | --- |
| Dependent Variable: DPPH | | | | | | | | |
| Light | Day | | Sum of Squares | df | Mean Square | F | Sig. | Partial Eta Squared |
| White light | Day 0 | Contrast | 3.195E-27 | 3 | 1.065E-27 | .000 | 1.000 | .000 |
|  |  | Error | 562.961 | 64 | 8.796 |  |  |  |
|  | Day 3 | Contrast | 30.460 | 3 | 10.153 | 1.154 | .334 | .051 |
|  |  | Error | 562.961 | 64 | 8.796 |  |  |  |
|  | Day 6 | Contrast | 184.042 | 3 | 61.347 | 6.974 | <.001 | .246 |
|  |  | Error | 562.961 | 64 | 8.796 |  |  |  |
|  | Day 9 | Contrast | 10.922 | 3 | 3.641 | .414 | .744 | .019 |
|  |  | Error | 562.961 | 64 | 8.796 |  |  |  |
| High blue light | Day 0 | Contrast | 2.919E-26 | 3 | 9.731E-27 | .000 | 1.000 | .000 |
|  |  | Error | 562.961 | 64 | 8.796 |  |  |  |
|  | Day 3 | Contrast | 598.343 | 3 | 199.448 | 22.674 | <.001 | .515 |
|  |  | Error | 562.961 | 64 | 8.796 |  |  |  |
|  | Day 6 | Contrast | 38.501 | 3 | 12.834 | 1.459 | .234 | .064 |
|  |  | Error | 562.961 | 64 | 8.796 |  |  |  |
|  | Day 9 | Contrast | 19.583 | 3 | 6.528 | .742 | .531 | .034 |
|  |  | Error | 562.961 | 64 | 8.796 |  |  |  |
| Each F tests the simple effects of SNP within each level combination of the other effects shown. These tests are based on the linearly independent pairwise comparisons among the estimated marginal means. | | | | | | | | |

| Pairwise Comparisons | | | | | | | | |
| --- | --- | --- | --- | --- | --- | --- | --- | --- |
| Dependent Variable: DPPH | | | | | | | | |
| Light | SNP (µM) | (I) Day | (J) Day | Mean Difference (I-J) | Std. Error | Sig.^b^ | 95% Confidence Interval for Difference^b^ | |
|  |  |  |  |  |  |  | Lower Bound | Upper Bound |
| White light | 0 | Day 0 | Day 3 | 7.838^*^ | 2.422 | .012 | 1.244 | 14.431 |
|  |  |  | Day 6 | 23.069^*^ | 2.422 | <.001 | 16.476 | 29.663 |
|  |  |  | Day 9 | 24.384^*^ | 2.422 | <.001 | 17.791 | 30.978 |
|  |  | Day 3 | Day 0 | -7.838^*^ | 2.422 | .012 | -14.431 | -1.244 |
|  |  |  | Day 6 | 15.232^*^ | 2.422 | <.001 | 8.638 | 21.825 |
|  |  |  | Day 9 | 16.547^*^ | 2.422 | <.001 | 9.953 | 23.140 |
|  |  | Day 6 | Day 0 | -23.069^*^ | 2.422 | <.001 | -29.663 | -16.476 |
|  |  |  | Day 3 | -15.232^*^ | 2.422 | <.001 | -21.825 | -8.638 |
|  |  |  | Day 9 | 1.315 | 2.422 | 1.000 | -5.278 | 7.908 |
|  |  | Day 9 | Day 0 | -24.384^*^ | 2.422 | <.001 | -30.978 | -17.791 |
|  |  |  | Day 3 | -16.547^*^ | 2.422 | <.001 | -23.140 | -9.953 |
|  |  |  | Day 6 | -1.315 | 2.422 | 1.000 | -7.908 | 5.278 |
|  | 100 | Day 0 | Day 3 | 7.833^*^ | 2.422 | .012 | 1.240 | 14.426 |
|  |  |  | Day 6 | 13.973^*^ | 2.422 | <.001 | 7.380 | 20.567 |
|  |  |  | Day 9 | 22.086^*^ | 2.422 | <.001 | 15.493 | 28.680 |
|  |  | Day 3 | Day 0 | -7.833^*^ | 2.422 | .012 | -14.426 | -1.240 |
|  |  |  | Day 6 | 6.140 | 2.422 | .082 | -.453 | 12.734 |
|  |  |  | Day 9 | 14.253^*^ | 2.422 | <.001 | 7.660 | 20.847 |
|  |  | Day 6 | Day 0 | -13.973^*^ | 2.422 | <.001 | -20.567 | -7.380 |
|  |  |  | Day 3 | -6.140 | 2.422 | .082 | -12.734 | .453 |
|  |  |  | Day 9 | 8.113^*^ | 2.422 | .008 | 1.520 | 14.706 |
|  |  | Day 9 | Day 0 | -22.086^*^ | 2.422 | <.001 | -28.680 | -15.493 |
|  |  |  | Day 3 | -14.253^*^ | 2.422 | <.001 | -20.847 | -7.660 |
|  |  |  | Day 6 | -8.113^*^ | 2.422 | .008 | -14.706 | -1.520 |
|  | 200 | Day 0 | Day 3 | 8.416^*^ | 2.422 | .006 | 1.822 | 15.009 |
|  |  |  | Day 6 | 13.063^*^ | 2.422 | <.001 | 6.470 | 19.656 |
|  |  |  | Day 9 | 23.994^*^ | 2.422 | <.001 | 17.400 | 30.587 |
|  |  | Day 3 | Day 0 | -8.416^*^ | 2.422 | .006 | -15.009 | -1.822 |
|  |  |  | Day 6 | 4.647 | 2.422 | .357 | -1.946 | 11.241 |
|  |  |  | Day 9 | 15.578^*^ | 2.422 | <.001 | 8.985 | 22.171 |
|  |  | Day 6 | Day 0 | -13.063^*^ | 2.422 | <.001 | -19.656 | -6.470 |
|  |  |  | Day 3 | -4.647 | 2.422 | .357 | -11.241 | 1.946 |
|  |  |  | Day 9 | 10.931^*^ | 2.422 | <.001 | 4.337 | 17.524 |
|  |  | Day 9 | Day 0 | -23.994^*^ | 2.422 | <.001 | -30.587 | -17.400 |
|  |  |  | Day 3 | -15.578^*^ | 2.422 | <.001 | -22.171 | -8.985 |
|  |  |  | Day 6 | -10.931^*^ | 2.422 | <.001 | -17.524 | -4.337 |
|  | 300 | Day 0 | Day 3 | 4.390 | 2.422 | .447 | -2.203 | 10.984 |
|  |  |  | Day 6 | 16.308^*^ | 2.422 | <.001 | 9.715 | 22.901 |
|  |  |  | Day 9 | 24.395^*^ | 2.422 | <.001 | 17.802 | 30.988 |
|  |  | Day 3 | Day 0 | -4.390 | 2.422 | .447 | -10.984 | 2.203 |
|  |  |  | Day 6 | 11.918^*^ | 2.422 | <.001 | 5.324 | 18.511 |
|  |  |  | Day 9 | 20.005^*^ | 2.422 | <.001 | 13.411 | 26.598 |
|  |  | Day 6 | Day 0 | -16.308^*^ | 2.422 | <.001 | -22.901 | -9.715 |
|  |  |  | Day 3 | -11.918^*^ | 2.422 | <.001 | -18.511 | -5.324 |
|  |  |  | Day 9 | 8.087^*^ | 2.422 | .008 | 1.494 | 14.680 |
|  |  | Day 9 | Day 0 | -24.395^*^ | 2.422 | <.001 | -30.988 | -17.802 |
|  |  |  | Day 3 | -20.005^*^ | 2.422 | <.001 | -26.598 | -13.411 |
|  |  |  | Day 6 | -8.087^*^ | 2.422 | .008 | -14.680 | -1.494 |
| High blue light | 0 | Day 0 | Day 3 | 26.727^*^ | 2.422 | <.001 | 20.133 | 33.320 |
|  |  |  | Day 6 | 23.593^*^ | 2.422 | <.001 | 17.000 | 30.187 |
|  |  |  | Day 9 | 34.520^*^ | 2.422 | <.001 | 27.926 | 41.113 |
|  |  | Day 3 | Day 0 | -26.727^*^ | 2.422 | <.001 | -33.320 | -20.133 |
|  |  |  | Day 6 | -3.133 | 2.422 | 1.000 | -9.726 | 3.460 |
|  |  |  | Day 9 | 7.793^*^ | 2.422 | .012 | 1.200 | 14.386 |
|  |  | Day 6 | Day 0 | -23.593^*^ | 2.422 | <.001 | -30.187 | -17.000 |
|  |  |  | Day 3 | 3.133 | 2.422 | 1.000 | -3.460 | 9.726 |
|  |  |  | Day 9 | 10.926^*^ | 2.422 | <.001 | 4.333 | 17.519 |
|  |  | Day 9 | Day 0 | -34.520^*^ | 2.422 | <.001 | -41.113 | -27.926 |
|  |  |  | Day 3 | -7.793^*^ | 2.422 | .012 | -14.386 | -1.200 |
|  |  |  | Day 6 | -10.926^*^ | 2.422 | <.001 | -17.519 | -4.333 |
|  | 100 | Day 0 | Day 3 | 29.328^*^ | 2.422 | <.001 | 22.734 | 35.921 |
|  |  |  | Day 6 | 23.432^*^ | 2.422 | <.001 | 16.839 | 30.026 |
|  |  |  | Day 9 | 36.030^*^ | 2.422 | <.001 | 29.436 | 42.623 |
|  |  | Day 3 | Day 0 | -29.328^*^ | 2.422 | <.001 | -35.921 | -22.734 |
|  |  |  | Day 6 | -5.895 | 2.422 | .106 | -12.489 | .698 |
|  |  |  | Day 9 | 6.702^*^ | 2.422 | .044 | .109 | 13.295 |
|  |  | Day 6 | Day 0 | -23.432^*^ | 2.422 | <.001 | -30.026 | -16.839 |
|  |  |  | Day 3 | 5.895 | 2.422 | .106 | -.698 | 12.489 |
|  |  |  | Day 9 | 12.597^*^ | 2.422 | <.001 | 6.004 | 19.191 |
|  |  | Day 9 | Day 0 | -36.030^*^ | 2.422 | <.001 | -42.623 | -29.436 |
|  |  |  | Day 3 | -6.702^*^ | 2.422 | .044 | -13.295 | -.109 |
|  |  |  | Day 6 | -12.597^*^ | 2.422 | <.001 | -19.191 | -6.004 |
|  | 200 | Day 0 | Day 3 | 17.627^*^ | 2.422 | <.001 | 11.034 | 24.221 |
|  |  |  | Day 6 | 21.366^*^ | 2.422 | <.001 | 14.773 | 27.960 |
|  |  |  | Day 9 | 35.736^*^ | 2.422 | <.001 | 29.142 | 42.329 |
|  |  | Day 3 | Day 0 | -17.627^*^ | 2.422 | <.001 | -24.221 | -11.034 |
|  |  |  | Day 6 | 3.739 | 2.422 | .765 | -2.854 | 10.332 |
|  |  |  | Day 9 | 18.109^*^ | 2.422 | <.001 | 11.515 | 24.702 |
|  |  | Day 6 | Day 0 | -21.366^*^ | 2.422 | <.001 | -27.960 | -14.773 |
|  |  |  | Day 3 | -3.739 | 2.422 | .765 | -10.332 | 2.854 |
|  |  |  | Day 9 | 14.370^*^ | 2.422 | <.001 | 7.776 | 20.963 |
|  |  | Day 9 | Day 0 | -35.736^*^ | 2.422 | <.001 | -42.329 | -29.142 |
|  |  |  | Day 3 | -18.109^*^ | 2.422 | <.001 | -24.702 | -11.515 |
|  |  |  | Day 6 | -14.370^*^ | 2.422 | <.001 | -20.963 | -7.776 |
|  | 300 | Day 0 | Day 3 | 11.705^*^ | 2.422 | <.001 | 5.111 | 18.298 |
|  |  |  | Day 6 | 19.192^*^ | 2.422 | <.001 | 12.599 | 25.786 |
|  |  |  | Day 9 | 32.784^*^ | 2.422 | <.001 | 26.190 | 39.377 |
|  |  | Day 3 | Day 0 | -11.705^*^ | 2.422 | <.001 | -18.298 | -5.111 |
|  |  |  | Day 6 | 7.488^*^ | 2.422 | .018 | .894 | 14.081 |
|  |  |  | Day 9 | 21.079^*^ | 2.422 | <.001 | 14.486 | 27.673 |
|  |  | Day 6 | Day 0 | -19.192^*^ | 2.422 | <.001 | -25.786 | -12.599 |
|  |  |  | Day 3 | -7.488^*^ | 2.422 | .018 | -14.081 | -.894 |
|  |  |  | Day 9 | 13.592^*^ | 2.422 | <.001 | 6.998 | 20.185 |
|  |  | Day 9 | Day 0 | -32.784^*^ | 2.422 | <.001 | -39.377 | -26.190 |
|  |  |  | Day 3 | -21.079^*^ | 2.422 | <.001 | -27.673 | -14.486 |
|  |  |  | Day 6 | -13.592^*^ | 2.422 | <.001 | -20.185 | -6.998 |
| Based on estimated marginal means | | | | | | | | |
| *. The mean difference is significant at the .05 level. | | | | | | | | |
| b. Adjustment for multiple comparisons: Bonferroni. | | | | | | | | |

| Univariate Tests | | | | | | | | |
| --- | --- | --- | --- | --- | --- | --- | --- | --- |
| Dependent Variable: DPPH | | | | | | | | |
| Light | SNP (µM) | | Sum of Squares | df | Mean Square | F | Sig. | Partial Eta Squared |
| White light | 0 | Contrast | 1271.808 | 3 | 423.936 | 48.195 | <.001 | .693 |
|  |  | Error | 562.961 | 64 | 8.796 |  |  |  |
|  | 100 | Contrast | 788.324 | 3 | 262.775 | 29.873 | <.001 | .583 |
|  |  | Error | 562.961 | 64 | 8.796 |  |  |  |
|  | 200 | Contrast | 900.685 | 3 | 300.228 | 34.131 | <.001 | .615 |
|  |  | Error | 562.961 | 64 | 8.796 |  |  |  |
|  | 300 | Contrast | 1115.969 | 3 | 371.990 | 42.290 | <.001 | .665 |
|  |  | Error | 562.961 | 64 | 8.796 |  |  |  |
| High blue light | 0 | Contrast | 1989.359 | 3 | 663.120 | 75.387 | <.001 | .779 |
|  |  | Error | 562.961 | 64 | 8.796 |  |  |  |
|  | 100 | Contrast | 2209.288 | 3 | 736.429 | 83.721 | <.001 | .797 |
|  |  | Error | 562.961 | 64 | 8.796 |  |  |  |
|  | 200 | Contrast | 1944.503 | 3 | 648.168 | 73.687 | <.001 | .775 |
|  |  | Error | 562.961 | 64 | 8.796 |  |  |  |
|  | 300 | Contrast | 1698.938 | 3 | 566.313 | 64.381 | <.001 | .751 |
|  |  | Error | 562.961 | 64 | 8.796 |  |  |  |
| Each F tests the simple effects of Day within each level combination of the other effects shown. These tests are based on the linearly independent pairwise comparisons among the estimated marginal means. | | | | | | | | |

**Post Hoc Tests**

| DPPH | | | | |
| --- | --- | --- | --- | --- |
| Duncan^a,b^ | | | | |
| SNP | N | Subset | | |
|  |  | 1 | 2 | 3 |
| 0 uM | 24 | 63.9176 |  |  |
| 100 uM | 24 | 64.8486 | 64.8486 |  |
| 200 uM | 24 |  | 66.4087 | 66.4087 |
| 300 uM | 24 |  |  | 67.8372 |
| Sig. |  | .281 | .073 | .100 |
| Means for groups in homogeneous subsets are displayed.  Based on observed means.  The error term is Mean Square(Error) = 8.796. | | | | |
| a. Uses Harmonic Mean Sample Size = 24.000. | | | | |
| b. Alpha = .05. | | | | |

| DPPH | | | | | |
| --- | --- | --- | --- | --- | --- |
| Duncan^a,b^ | | | | | |
| Day | N | Subset | | | |
|  |  | 1 | 2 | 3 | 4 |
| Day 9 | 24 | 52.1929 |  |  |  |
| Day 6 | 24 |  | 62.1842 |  |  |
| Day 3 | 24 |  |  | 67.2011 |  |
| Day 0 | 24 |  |  |  | 81.4339 |
| Sig. |  | 1.000 | 1.000 | 1.000 | 1.000 |
| Means for groups in homogeneous subsets are displayed.  Based on observed means.  The error term is Mean Square(Error) = 8.796. | | | | | |
| a. Uses Harmonic Mean Sample Size = 24.000. | | | | | |
| b. Alpha = .05. | | | | | |

**The results of the statistical analysis using a two-way ANOVA**

| Tests of Between-Subjects Effects | | | | | | |
| --- | --- | --- | --- | --- | --- | --- |
| Dependent Variable: Linalool | | | | | | |
| Source | Type III Sum of Squares | df | Mean Square | F | Sig. | Partial Eta Squared |
| Corrected Model | 3.658E-7^a^ | 7 | 5.226E-8 | 3.539 | .017 | .608 |
| Intercept | 9.174E-6 | 1 | 9.174E-6 | 621.308 | <.001 | .975 |
| SNP | 2.470E-7 | 3 | 8.233E-8 | 5.575 | .008 | .511 |
| Light | 1.449E-9 | 1 | 1.449E-9 | .098 | .758 | .006 |
| SNP * Light | 1.174E-7 | 3 | 3.914E-8 | 2.651 | .084 | .332 |
| Error | 2.363E-7 | 16 | 1.477E-8 |  |  |  |
| Total | 9.776E-6 | 24 |  |  |  |  |
| Corrected Total | 6.021E-7 | 23 |  |  |  |  |
| a. R Squared = .608 (Adjusted R Squared = .436) | | | | | | |

| Tests of Between-Subjects Effects | | | | | | |
| --- | --- | --- | --- | --- | --- | --- |
| Dependent Variable: Beta_caryophyllene | | | | | | |
| Source | Type III Sum of Squares | df | Mean Square | F | Sig. | Partial Eta Squared |
| Corrected Model | .003^a^ | 7 | .000 | 4.804 | .004 | .678 |
| Intercept | .022 | 1 | .022 | 250.665 | <.001 | .940 |
| SNP | .002 | 3 | .001 | 6.022 | .006 | .530 |
| Light | .001 | 1 | .001 | 8.496 | .010 | .347 |
| SNP * Light | .001 | 3 | .000 | 2.356 | .110 | .306 |
| Error | .001 | 16 | 8.916E-5 |  |  |  |
| Total | .027 | 24 |  |  |  |  |
| Corrected Total | .004 | 23 |  |  |  |  |
| a. R Squared = .678 (Adjusted R Squared = .537) | | | | | | |

| Tests of Between-Subjects Effects | | | | | | |
| --- | --- | --- | --- | --- | --- | --- |
| Dependent Variable: Humulene | | | | | | |
| Source | Type III Sum of Squares | df | Mean Square | F | Sig. | Partial Eta Squared |
| Corrected Model | 5.232E-6^a^ | 7 | 7.475E-7 | 5.284 | .003 | .698 |
| Intercept | .000 | 1 | .000 | 853.680 | <.001 | .982 |
| SNP | 3.743E-6 | 3 | 1.248E-6 | 8.819 | .001 | .623 |
| Light | 6.069E-7 | 1 | 6.069E-7 | 4.290 | .055 | .211 |
| SNP * Light | 8.830E-7 | 3 | 2.943E-7 | 2.081 | .143 | .281 |
| Error | 2.263E-6 | 16 | 1.415E-7 |  |  |  |
| Total | .000 | 24 |  |  |  |  |
| Corrected Total | 7.496E-6 | 23 |  |  |  |  |
| a. R Squared = .698 (Adjusted R Squared = .566) | | | | | | |

| Tests of Between-Subjects Effects | | | | | | |
| --- | --- | --- | --- | --- | --- | --- |
| Dependent Variable: Methyl_eugenol | | | | | | |
| Source | Type III Sum of Squares | df | Mean Square | F | Sig. | Partial Eta Squared |
| Corrected Model | .004^a^ | 7 | .001 | 1.342 | .294 | .370 |
| Intercept | .201 | 1 | .201 | 452.563 | <.001 | .966 |
| SNP | .003 | 3 | .001 | 2.342 | .112 | .305 |
| Light | .000 | 1 | .000 | .932 | .349 | .055 |
| SNP * Light | .001 | 3 | .000 | .480 | .701 | .083 |
| Error | .007 | 16 | .000 |  |  |  |
| Total | .212 | 24 |  |  |  |  |
| Corrected Total | .011 | 23 |  |  |  |  |
| a. R Squared = .370 (Adjusted R Squared = .094) | | | | | | |

| Tests of Between-Subjects Effects | | | | | | |
| --- | --- | --- | --- | --- | --- | --- |
| Dependent Variable: Eugenol | | | | | | |
| Source | Type III Sum of Squares | df | Mean Square | F | Sig. | Partial Eta Squared |
| Corrected Model | .002^a^ | 7 | .000 | 2.460 | .064 | .518 |
| Intercept | .006 | 1 | .006 | 60.440 | <.001 | .791 |
| SNP | .000 | 3 | .000 | 1.039 | .402 | .163 |
| Light | 3.434E-5 | 1 | 3.434E-5 | .344 | .566 | .021 |
| SNP * Light | .001 | 3 | .000 | 4.588 | .017 | .462 |
| Error | .002 | 16 | 9.981E-5 |  |  |  |
| Total | .009 | 24 |  |  |  |  |
| Corrected Total | .003 | 23 |  |  |  |  |
| a. R Squared = .518 (Adjusted R Squared = .308) | | | | | | |

**Supplementary Document S3:**

**Pre-harvest blue light exposure and postharvest sodium nitroprusside application enhance chilling tolerance and improve storage quality of holy basil**

Thanaboon Plakunmonthon, Panita Chutimanukul, Kenji Matsui, and Kanogwan Seraypheap^*^

*Corresponding author: Kanogwan Seraypheap

Department of Botany, Faculty of Science, Tel.: +66-2-218-5495, Fax: +66-2-252-8979

E-mail: [kanogwan.k@chula.ac.th](mailto:kanogwan.k@chula.ac.th)


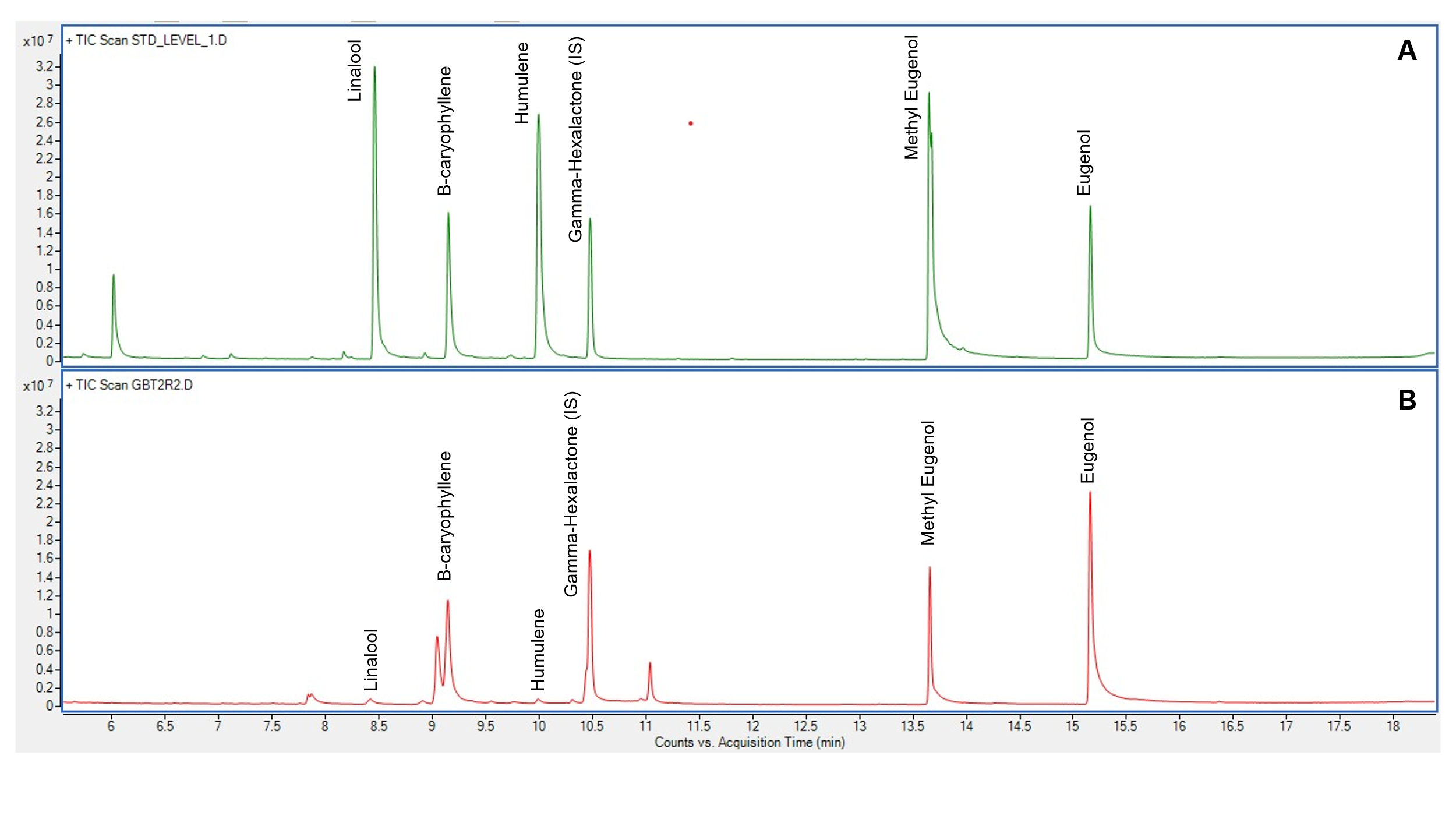


**Figure S3.** GC–QTOF chromatograms of authentic standard compounds (A) and the holy basil

sample (B).

**Table S3**. Coefficients of determination (R²) for the calibration curves.

| Compound | Coefficient of Determination (R²) |
| --- | --- |
| Linalool | 0.9999 |
| Beta-caryophyllene | 0.9970 |
| Humulene (alpha-caryophyllene) | 0.9991 |
| Methyl eugenol | 0.9967 |
| Eugenol | 0.9999 |
